# Supplementary material for: Design, Synthesis, and Bioactivity Evaluation of New Thiochromanone Derivatives Containing a Carboxamide Moiety
Source: Molecules. 2021 Jul 21;26(15):4391. doi: 10.3390/molecules26154391 (PMC8348251; doi:10.3390/molecules26154391)
Supplement: Supplementary file 1 [file molecules-26-04391-s001.zip › molecules-1293168-supplementary.pdf]

molecules

MDPI

Supplementary

Design, Synthesis, and Bioactivity Evaluation of New Thiochromanone Derivatives Containing a Carboxamide Moiety

Lingling Xiao [1,†], Lu Yu [1,†], Pei Li [1,2,*], Jiyan Chi [1], Zhangfei Tang [1], Jie Li [1], Shuming Tan [1,*] and Xiaodan Wang [1,3]

[1] School of Liquor and Food Engineering, Guizhou University, Guiyang 550025, China; an1378386891@163.com (L.X.), lyu1@gzu.edu.cn (L.Y.), qq1401064120@126.com (J.C.), tzf18885247153@163.com (Z.T.), sushilee0120@163.com (J.L.), wangxiaodan0516@126.com (X.W.)

[2] Qiandongnan Engineering and Technology Research Center for Comprehensive Utilization of National Medicine/Key Laboratory for Modernization of Qiandongnan Miao & Dong Medicine, Kaili University, Kaili 556011, China

[3] Guizhou Provincial Key Laboratory of Fermentation Engineering and Biological Pharmacy, Guizhou University, Guiyang 550025, China

* Correspondence: lipei@kluniv.edu.cn (P.L.), smtan@gzu.edu.cn (S.T.); Tel.: +86(0851)8559466

† Lingling Xiao and Lu Yu contributed equally to this work.

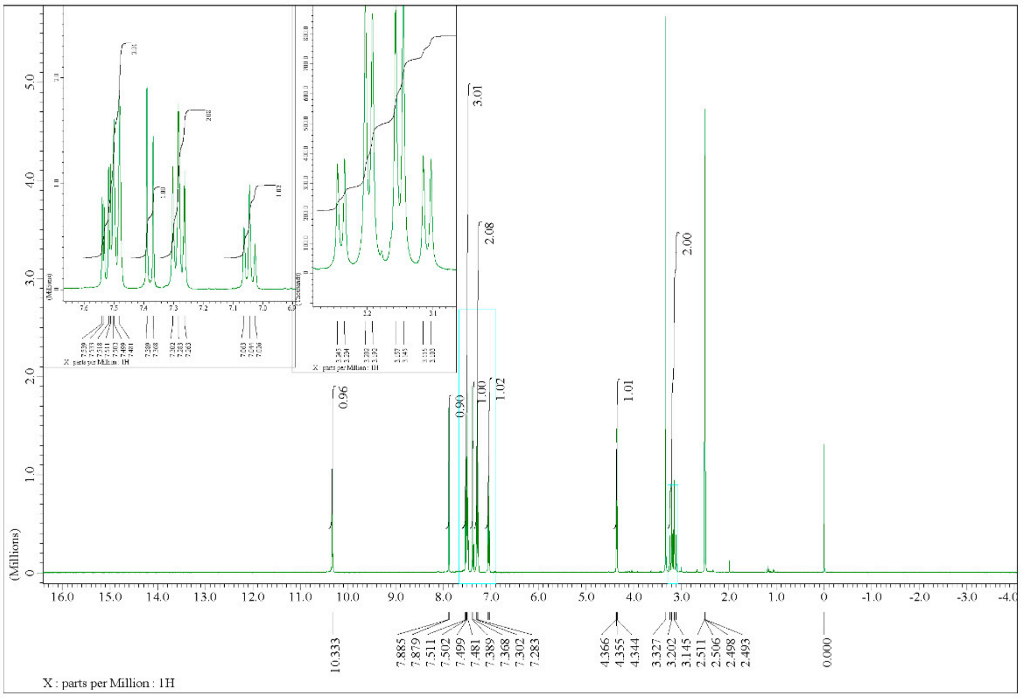

^{1}H NMR of compound 3a

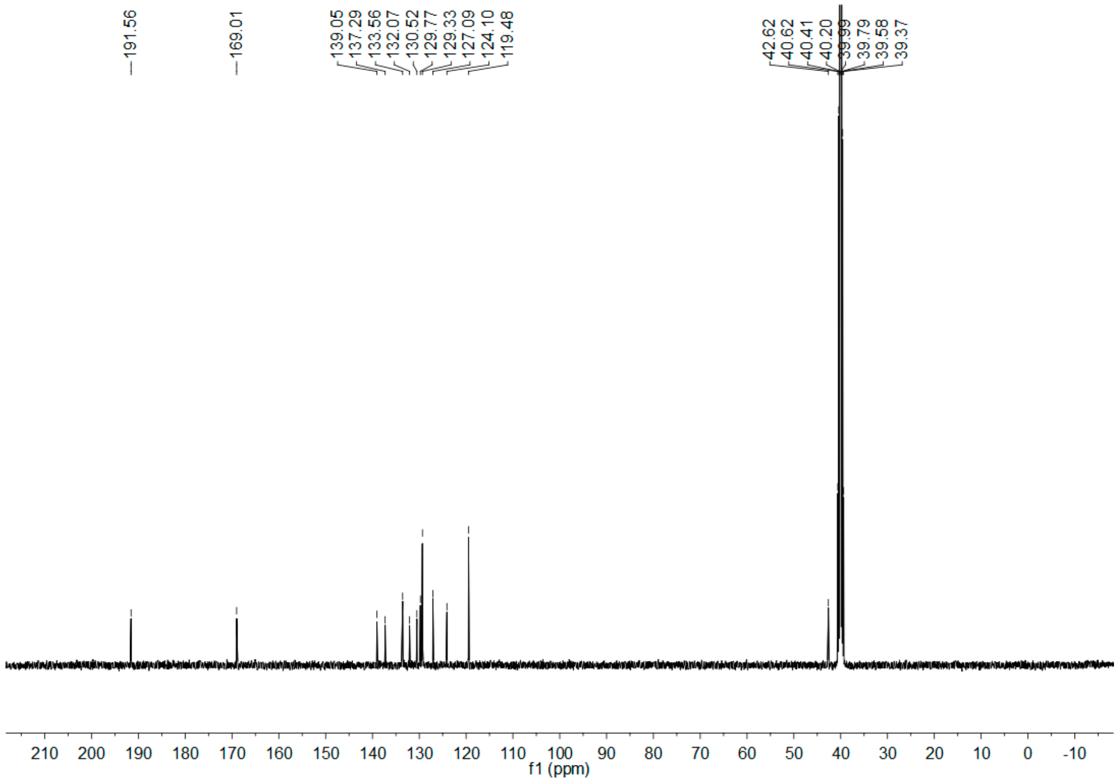

^{13}C NMR of compound 3a

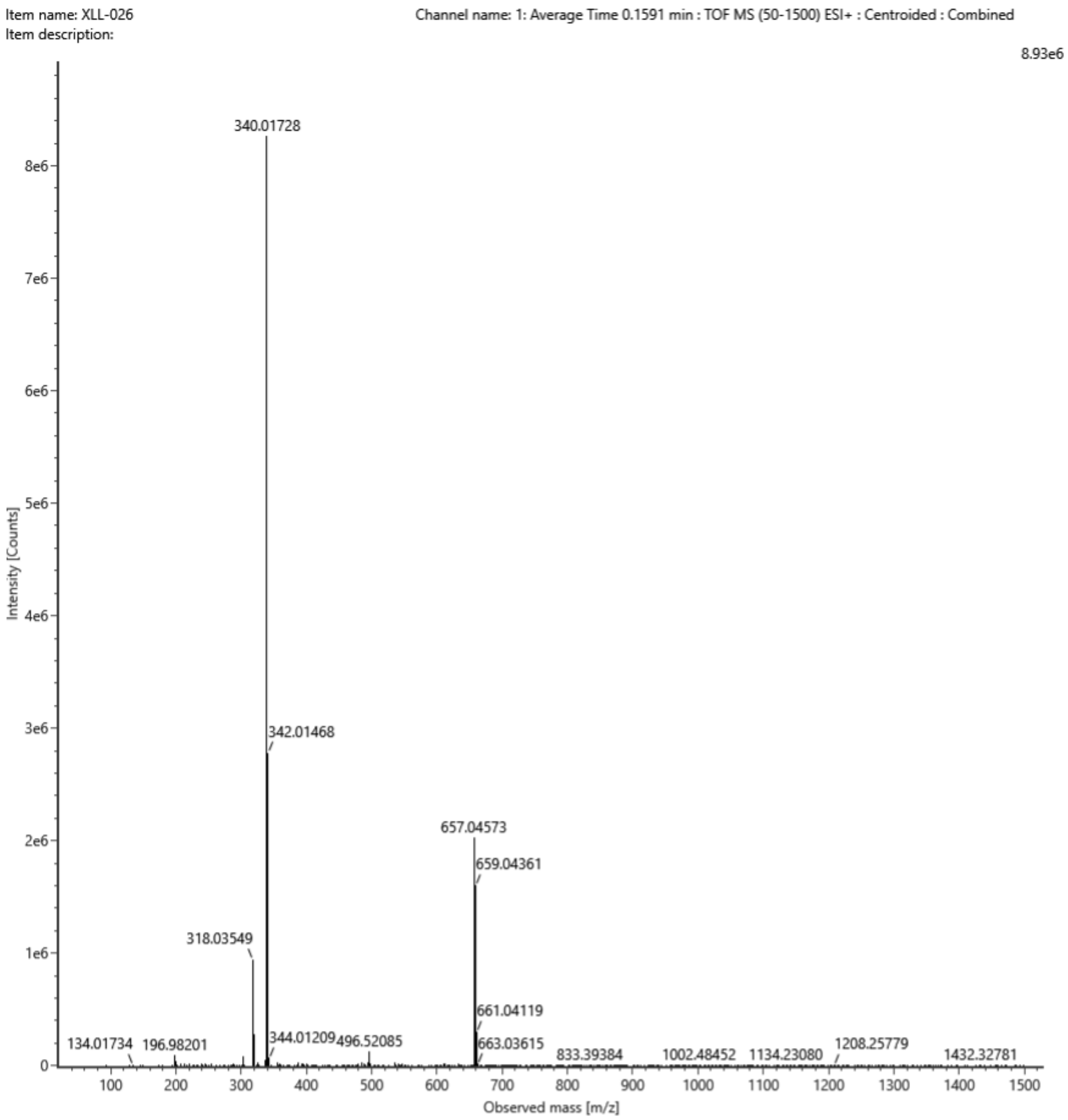

HRMS of compound 3a

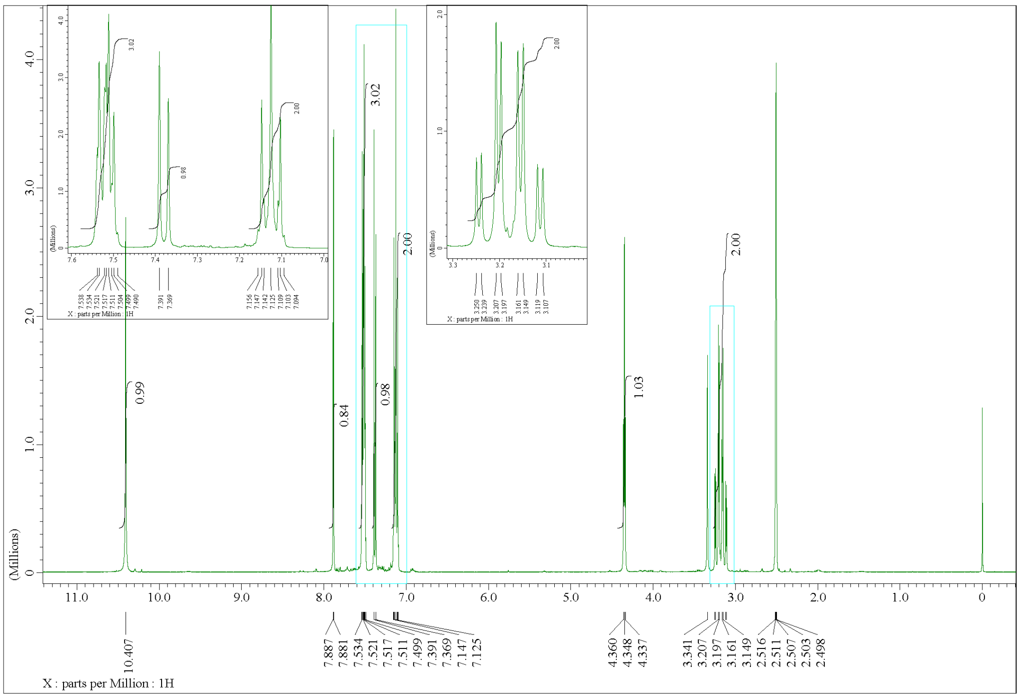

^{1}H NMR of compound 3b

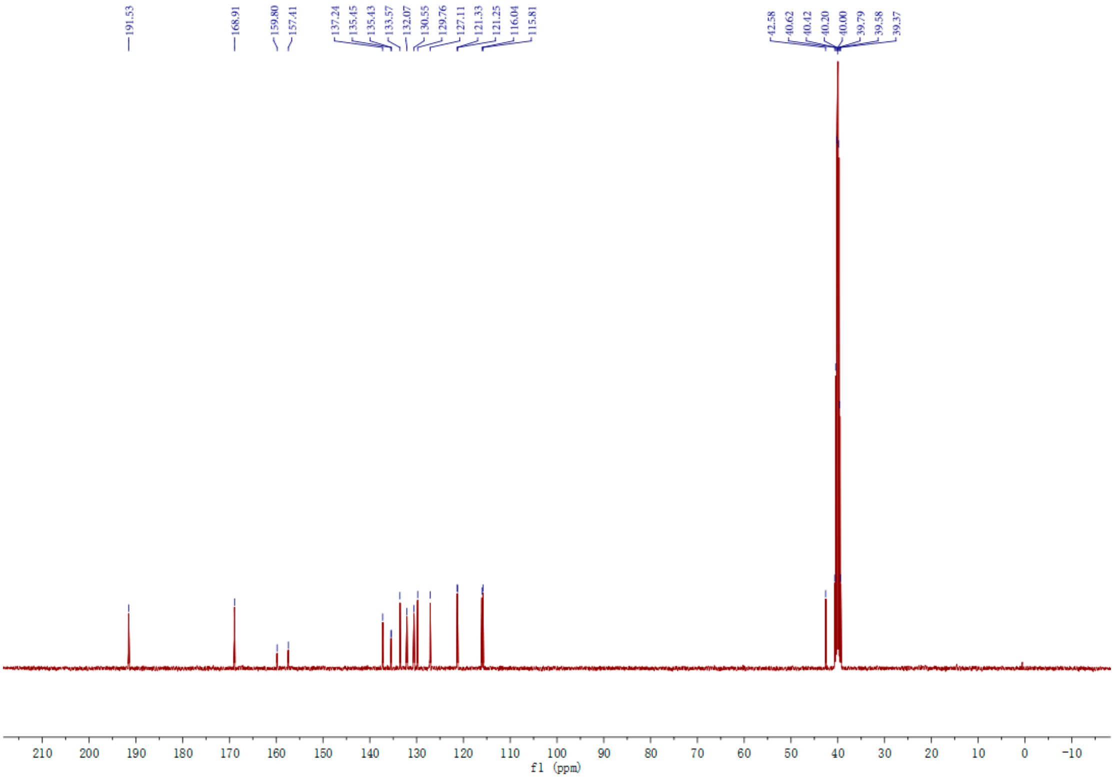

^{13}C NMR of compound 3b

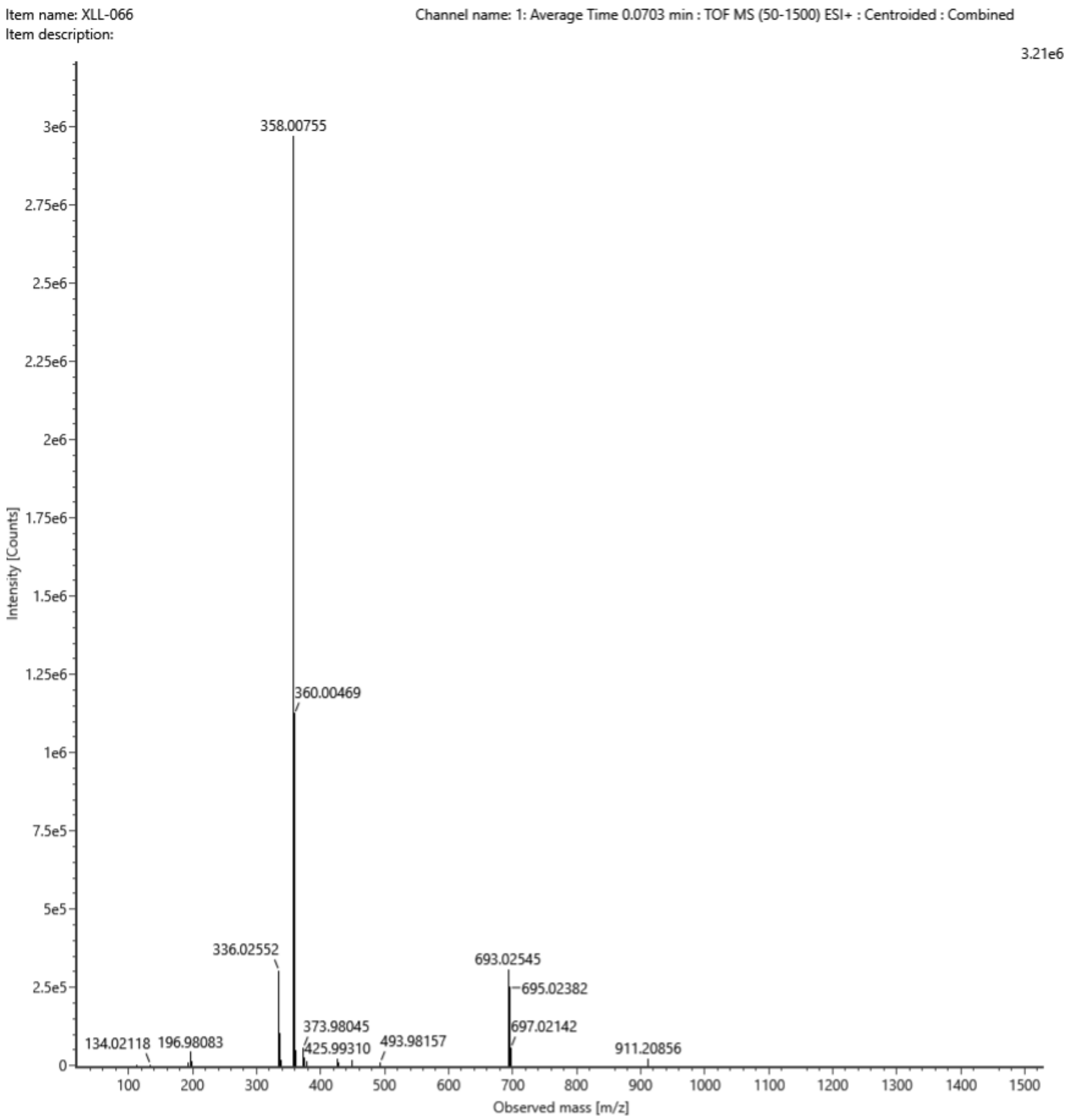

HRMS of compound 3b

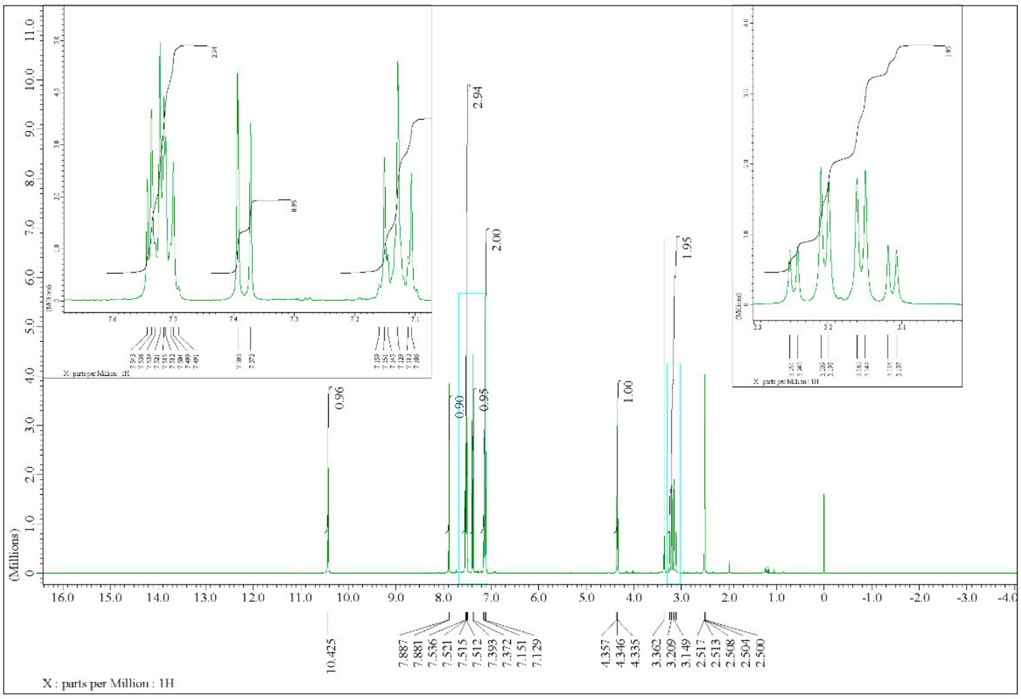

^{1}H NMR of compound 3c

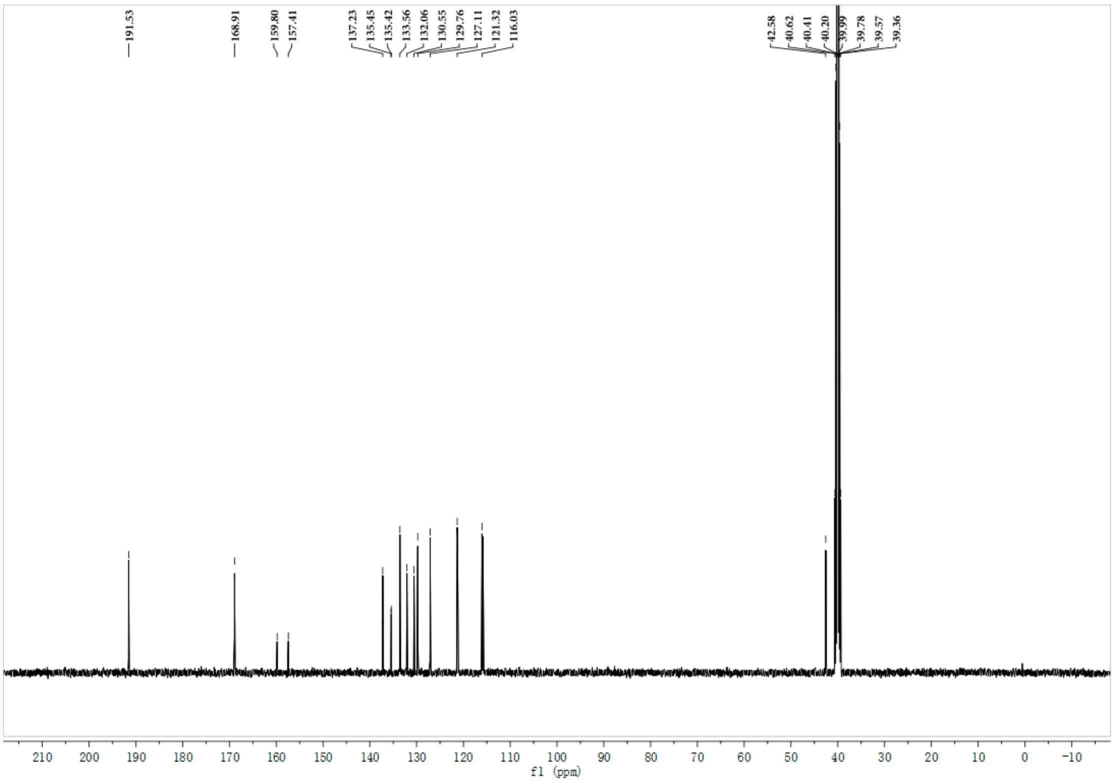

^{13}C NMR of compound 3c

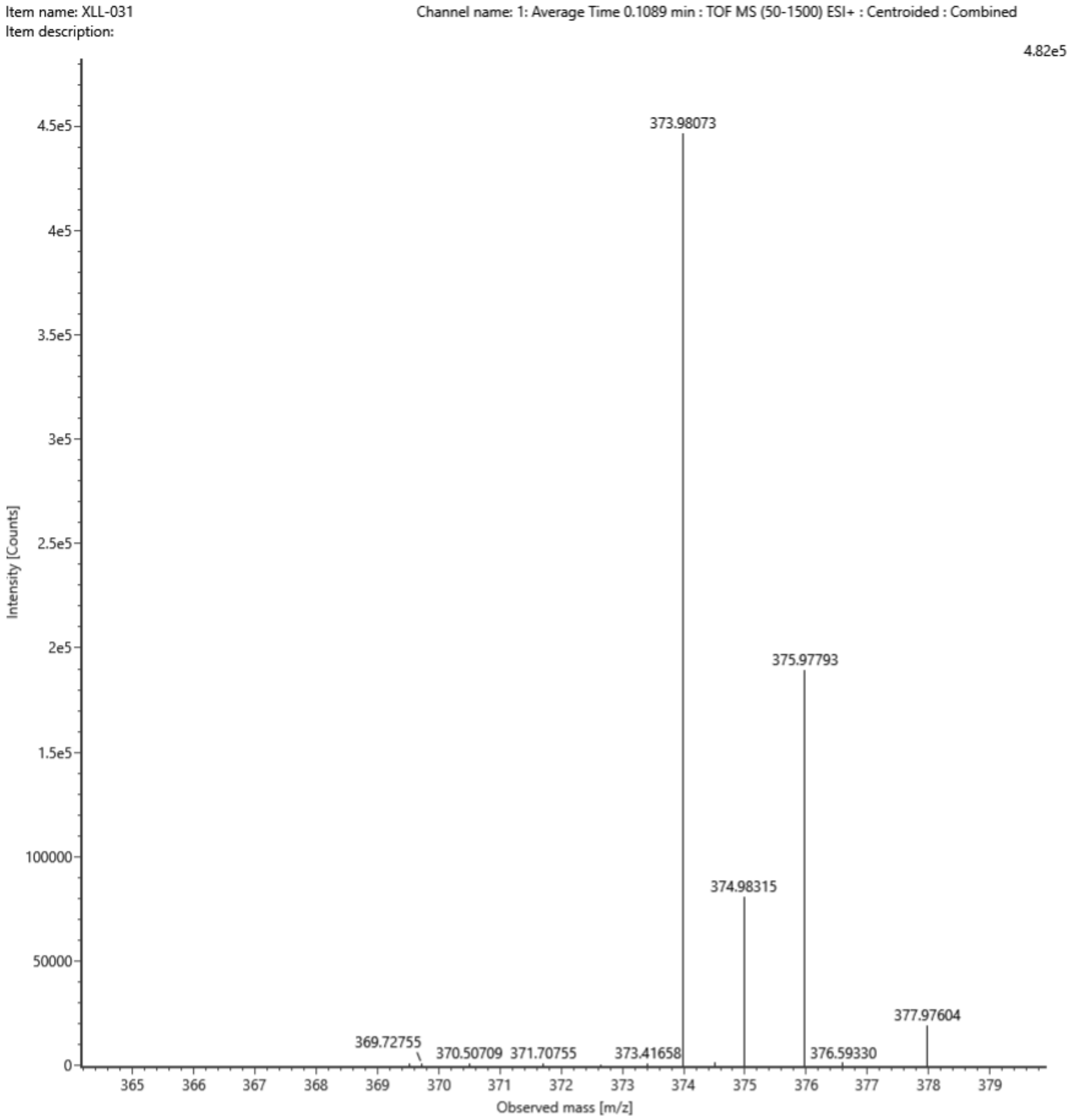

HRMS of compound 3c

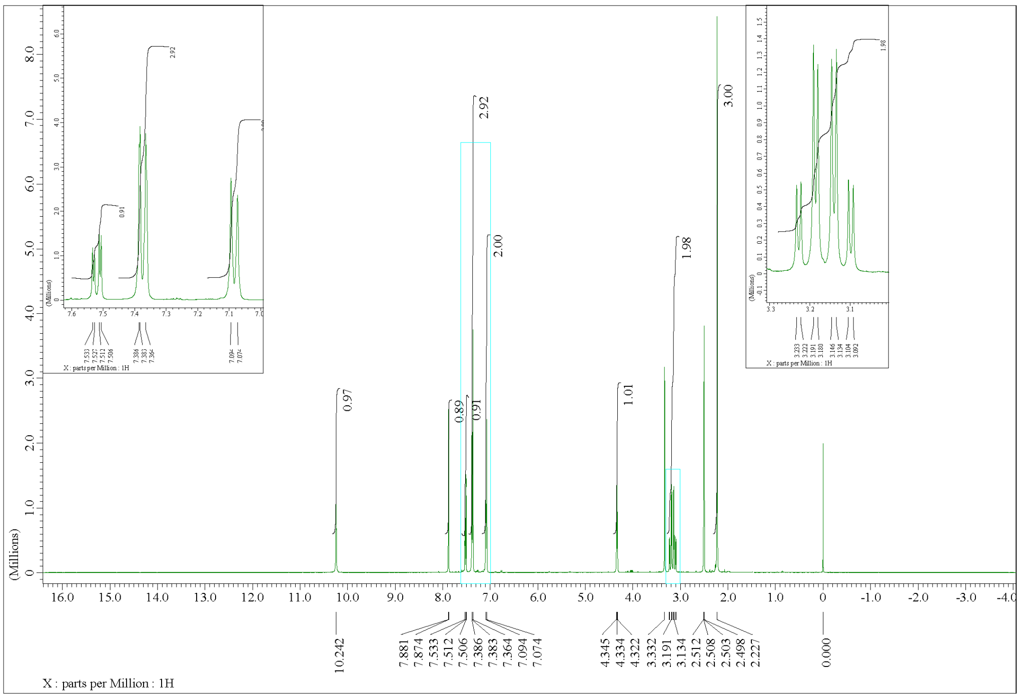

^{1}H NMR of compound 3d

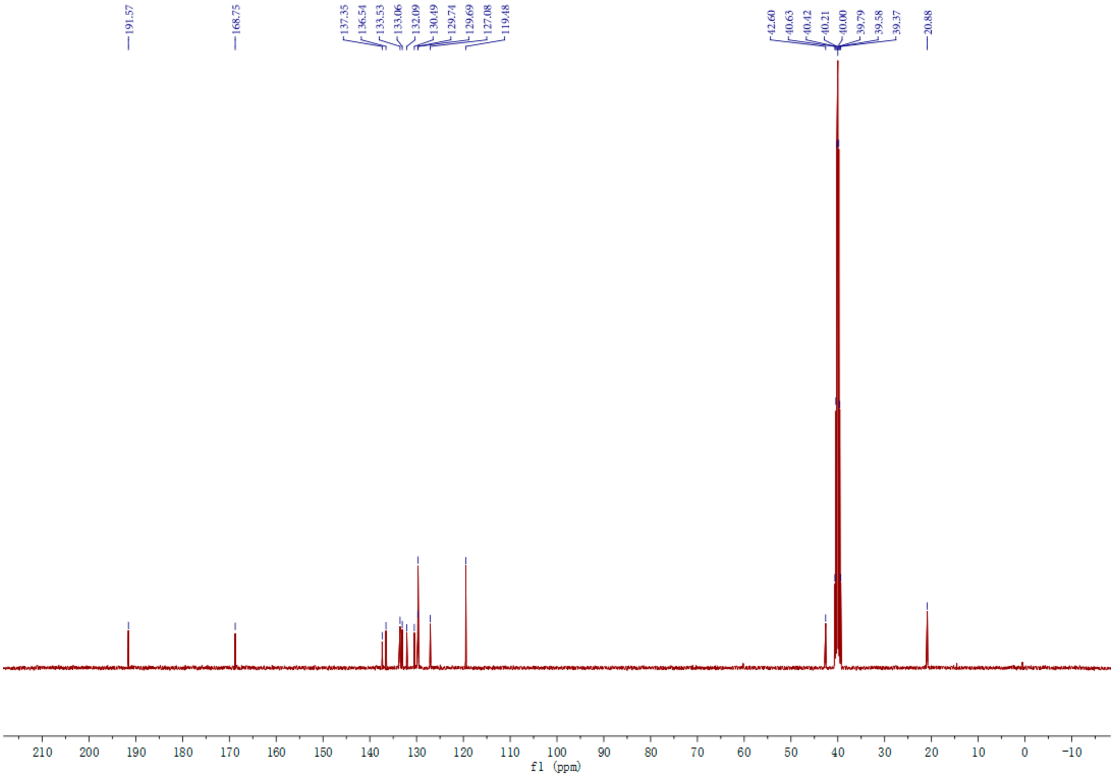

^{13}C NMR of compound 3d

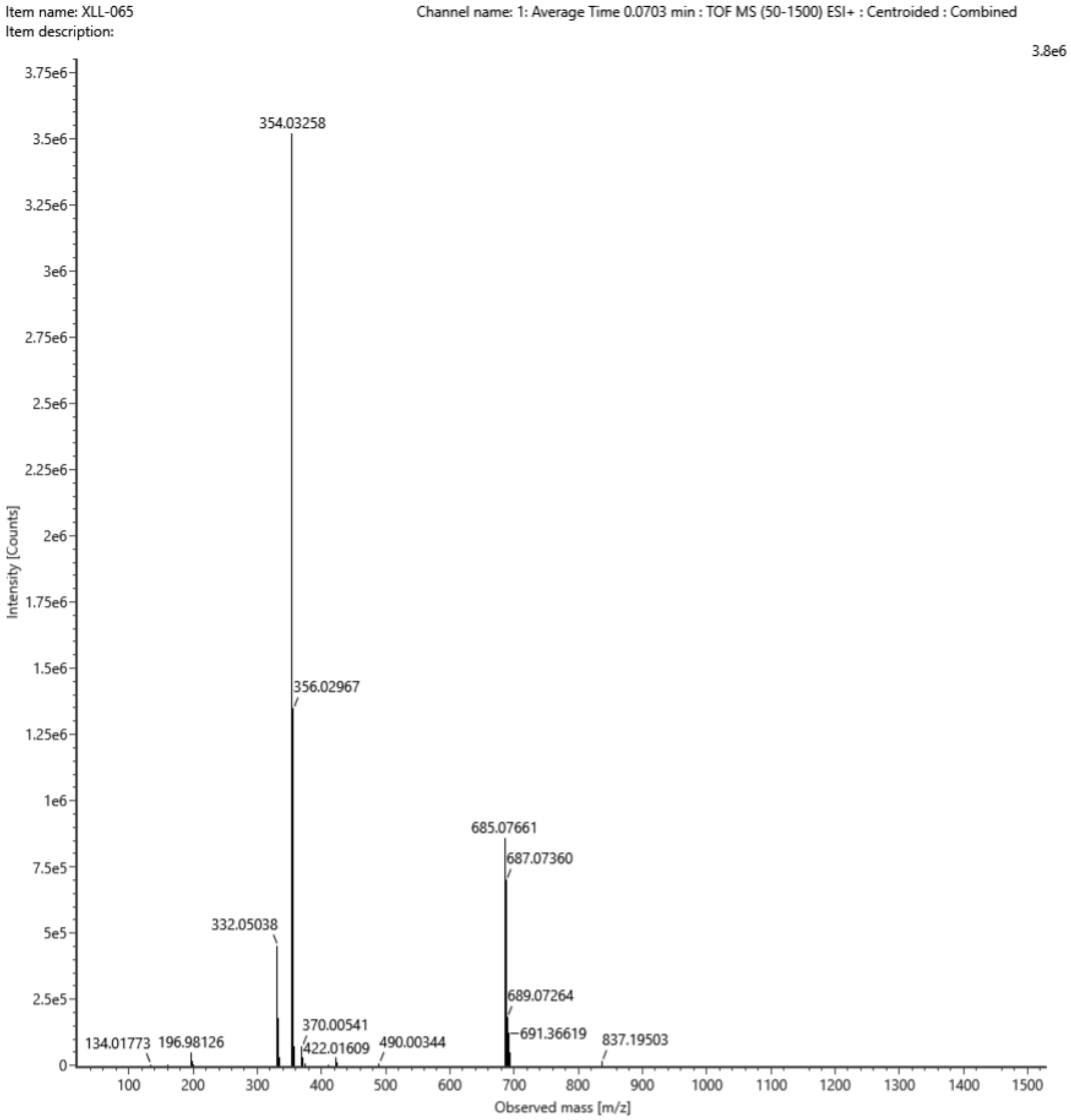

HRMS of compound 3d

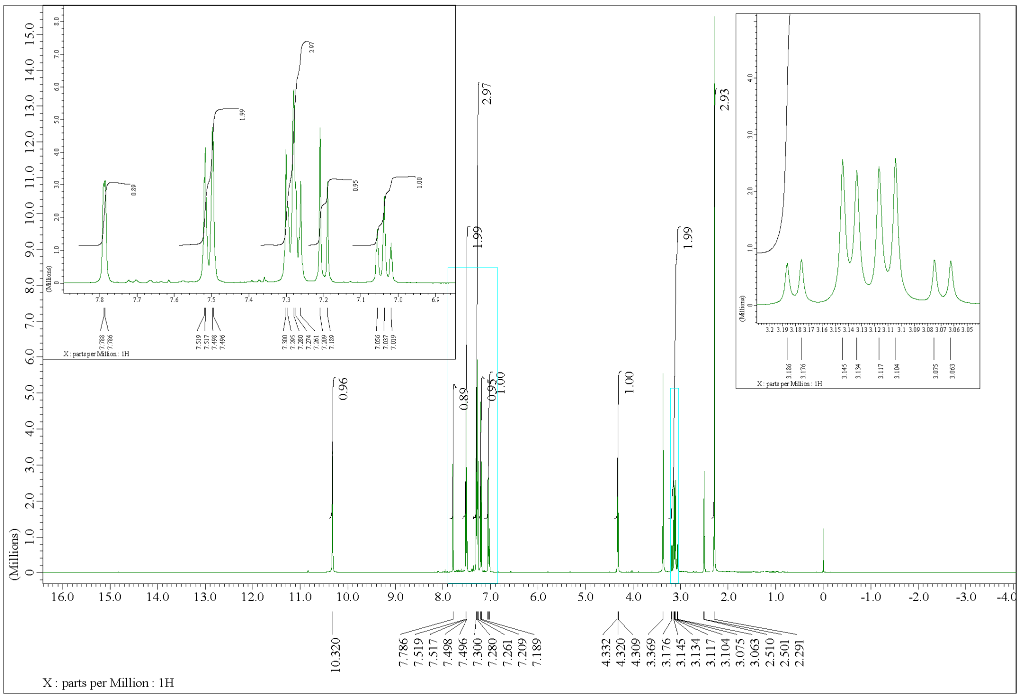

^{1}H NMR of compound 3e

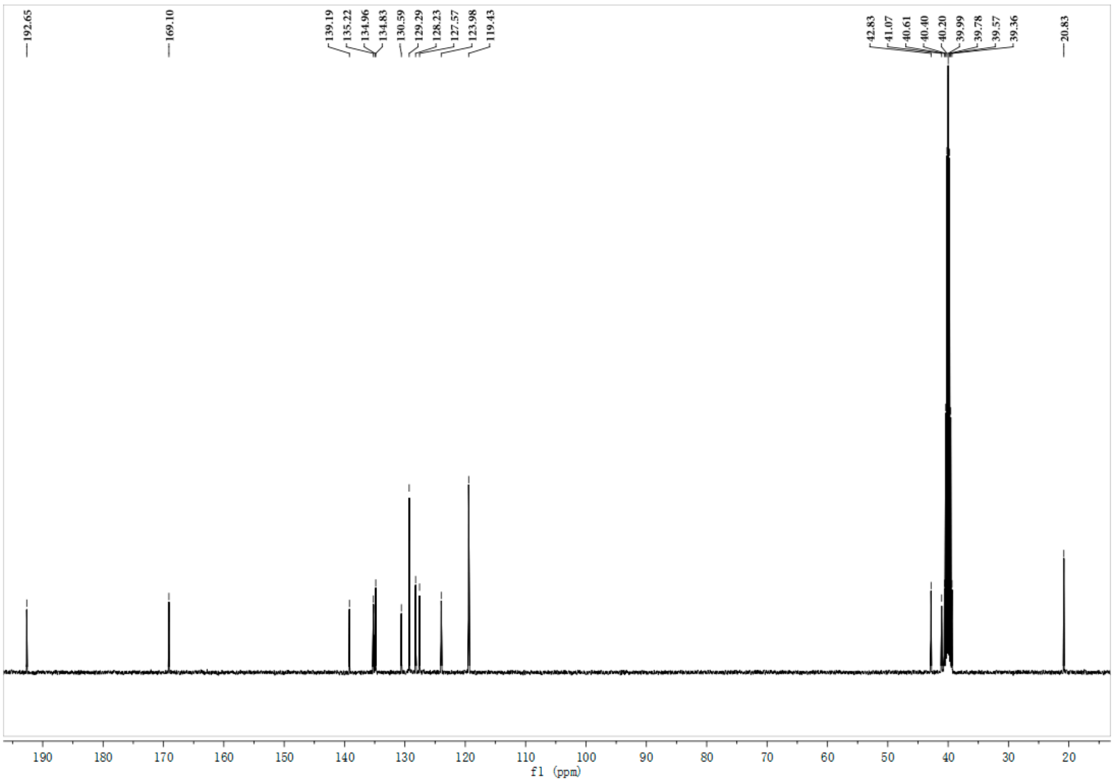

^{13}C NMR of compound 3e

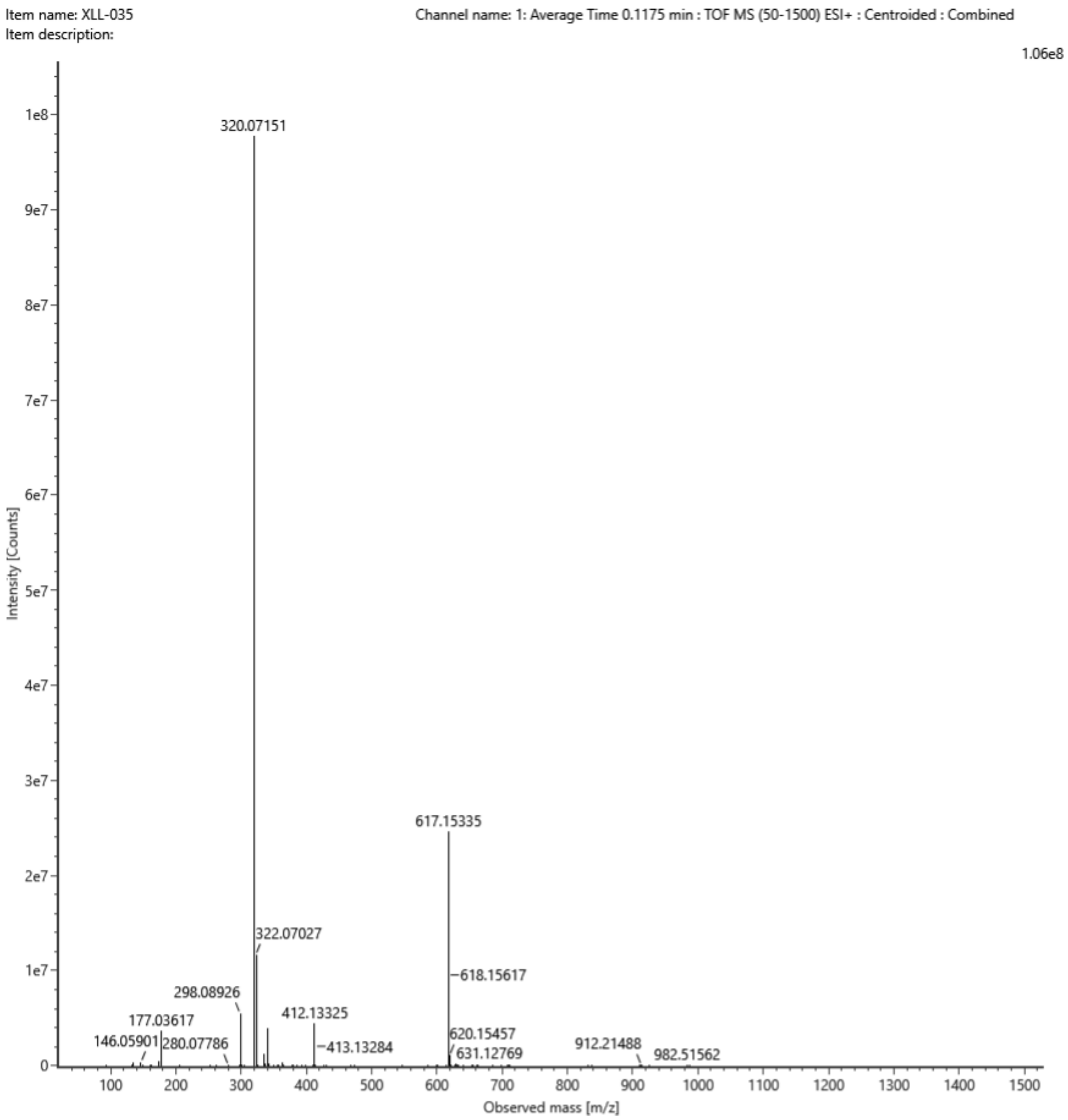

HRMS of compound 3e

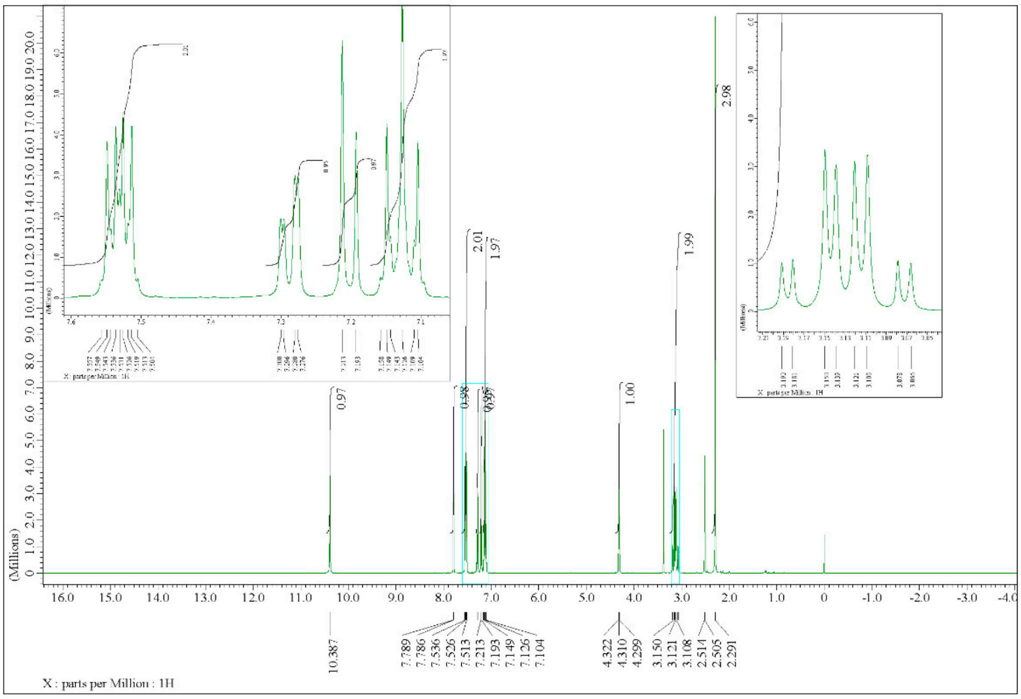

^{1}H NMR of compound 3f

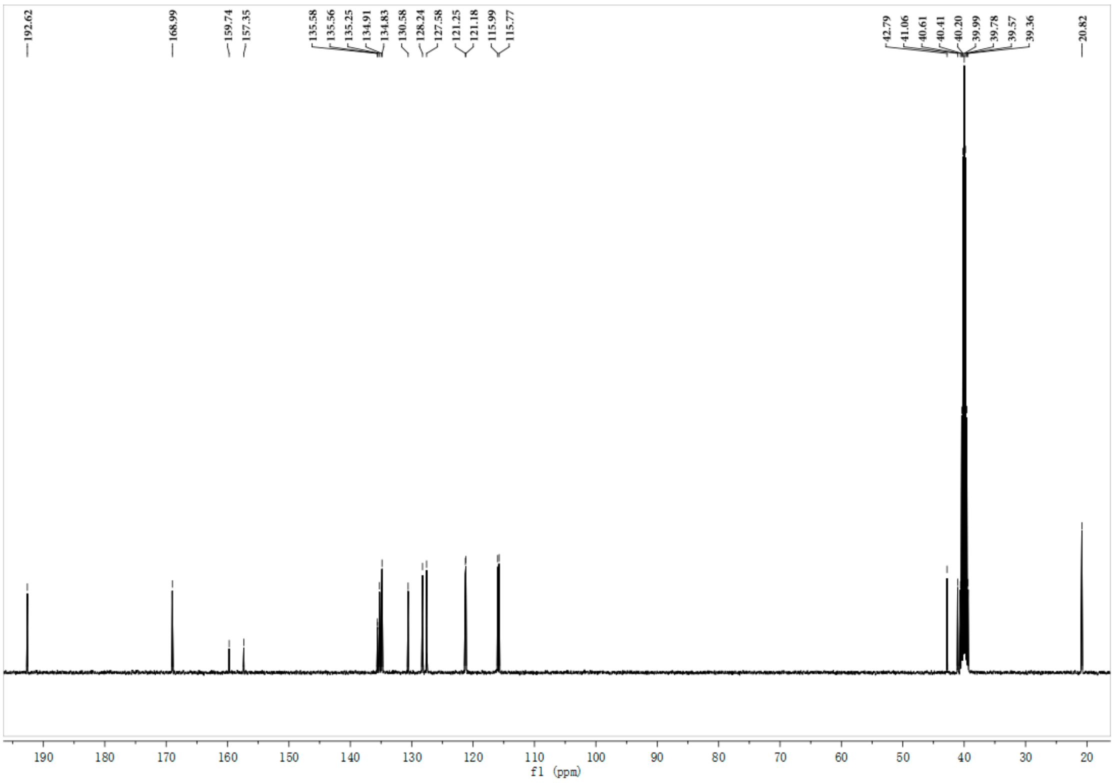

^{13}C NMR of compound 3f

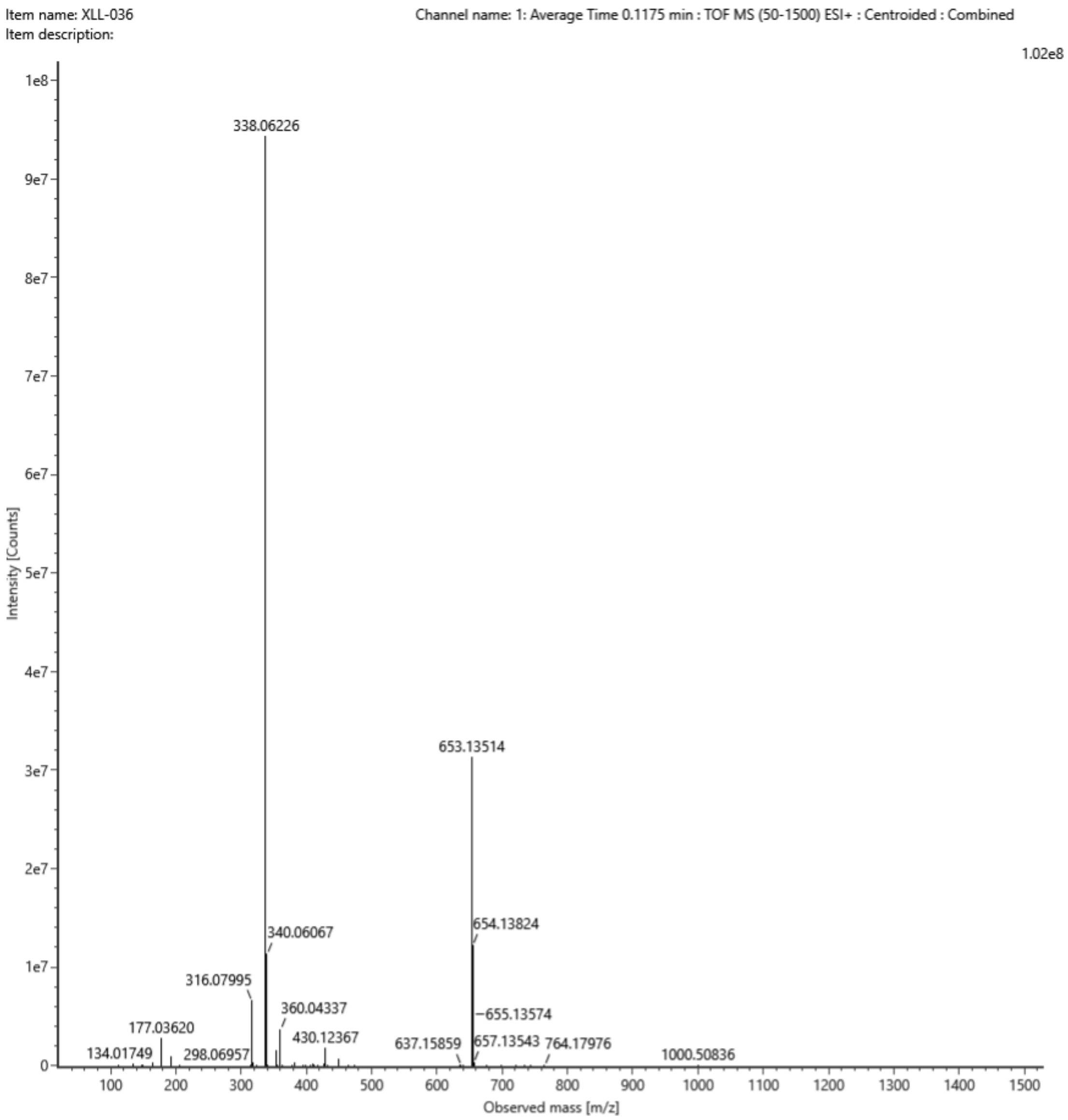

HRMS of compound 3f

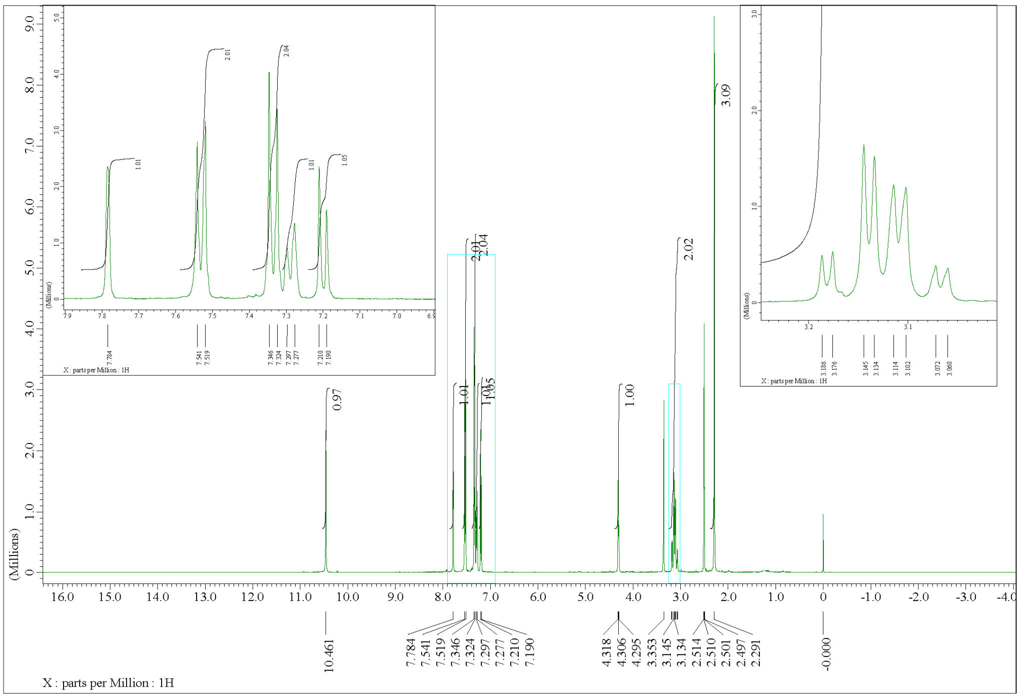

^{1}H NMR of compound 3g

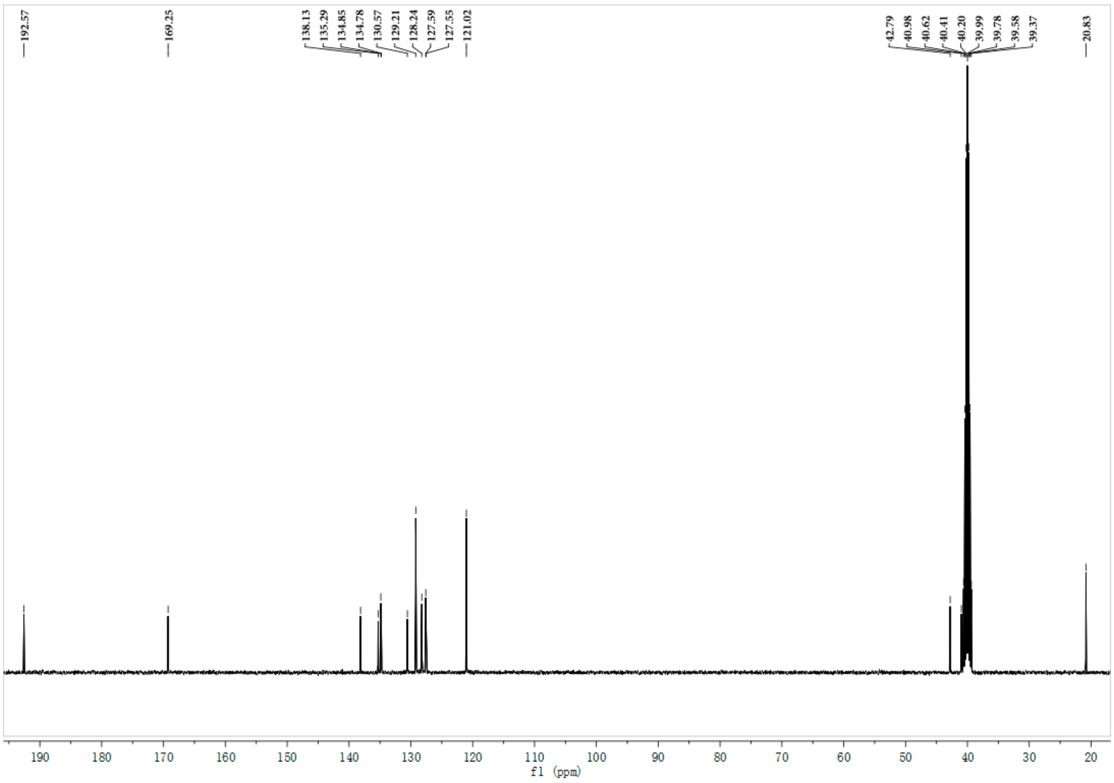

^{13}C NMR of compound 3g

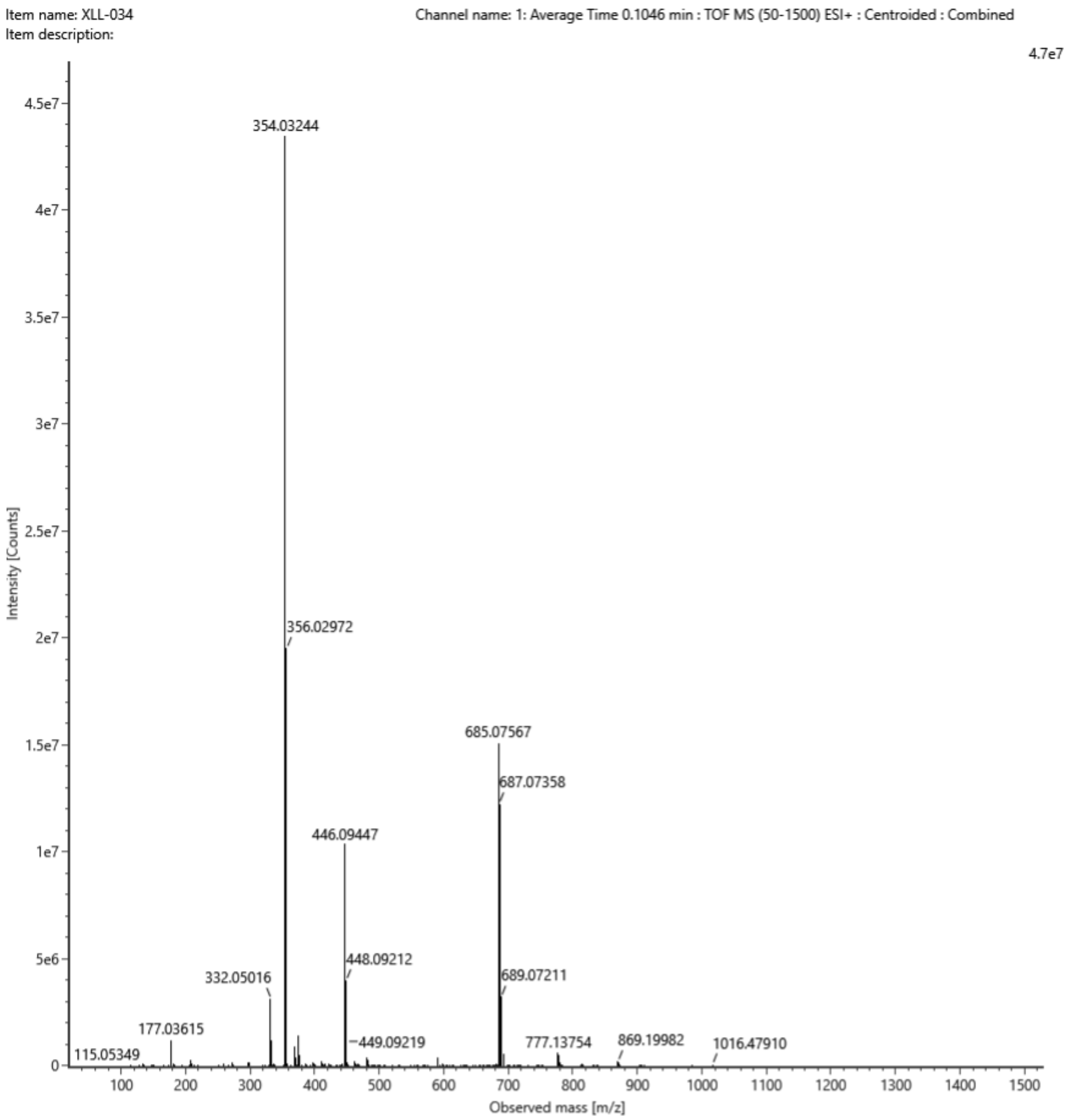

HRMS of compound 3g

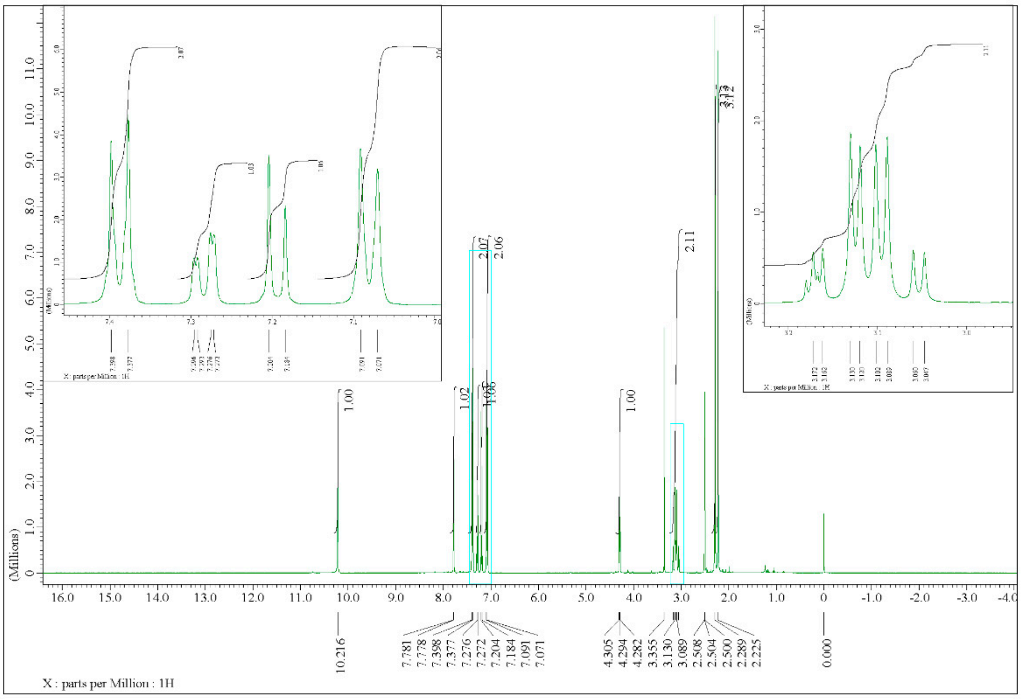

^{1}H NMR of compound 3h

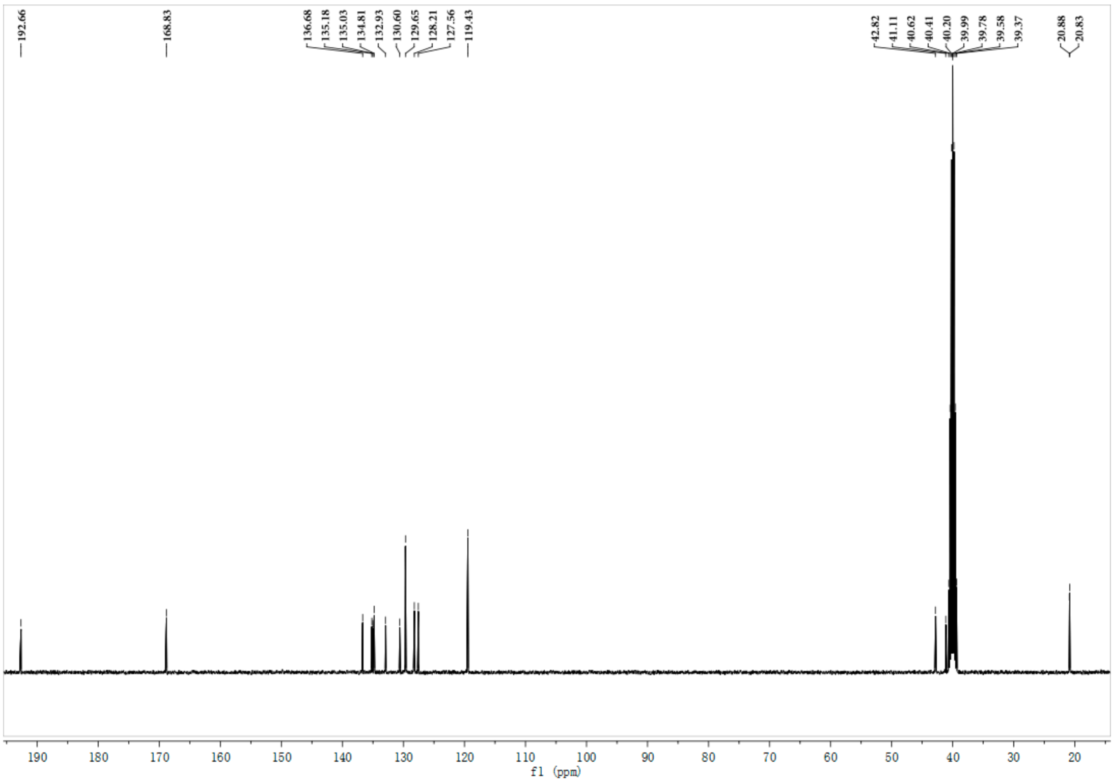

^{13}C NMR of compound 3h

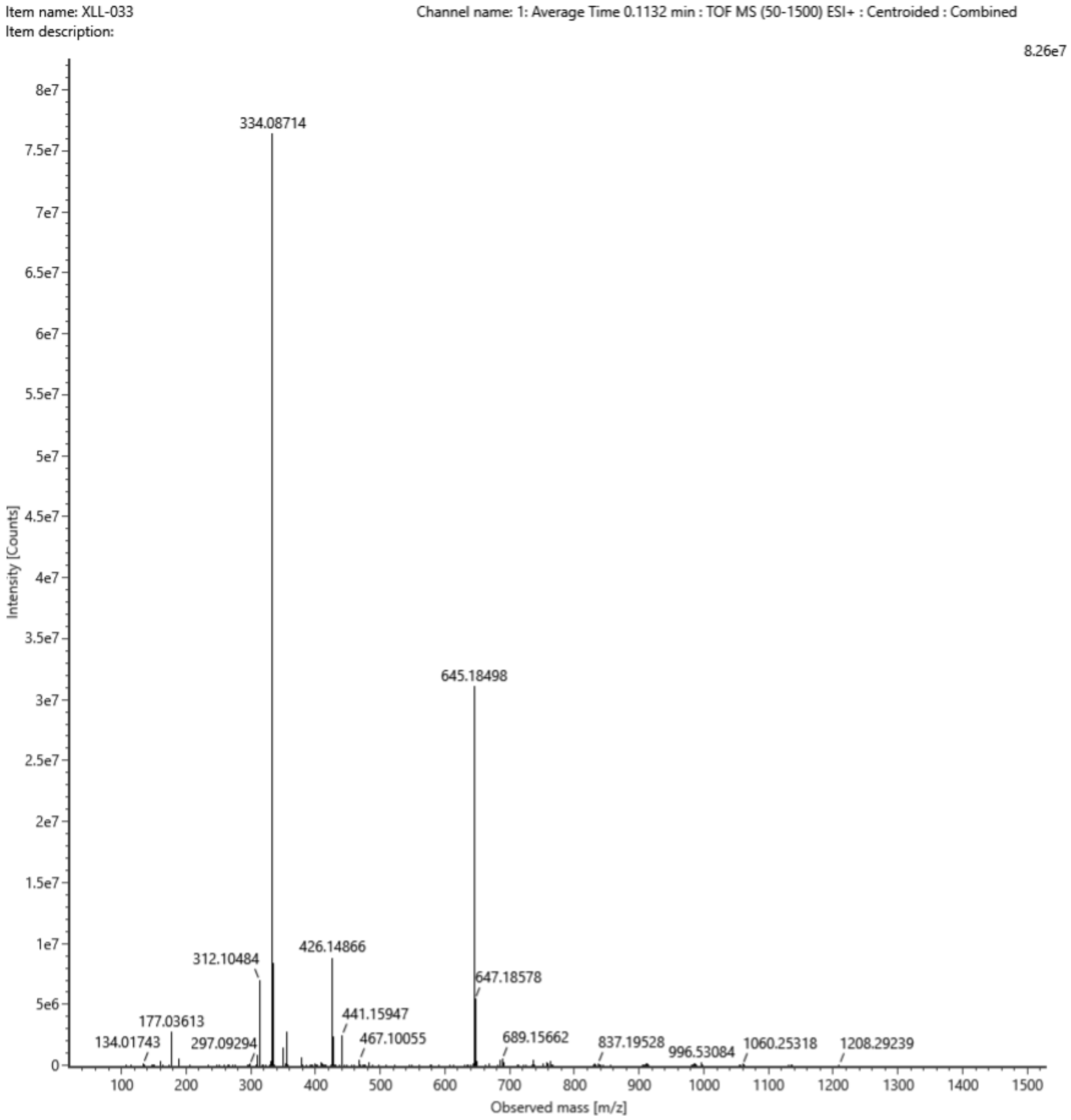

HRMS of compound 3h

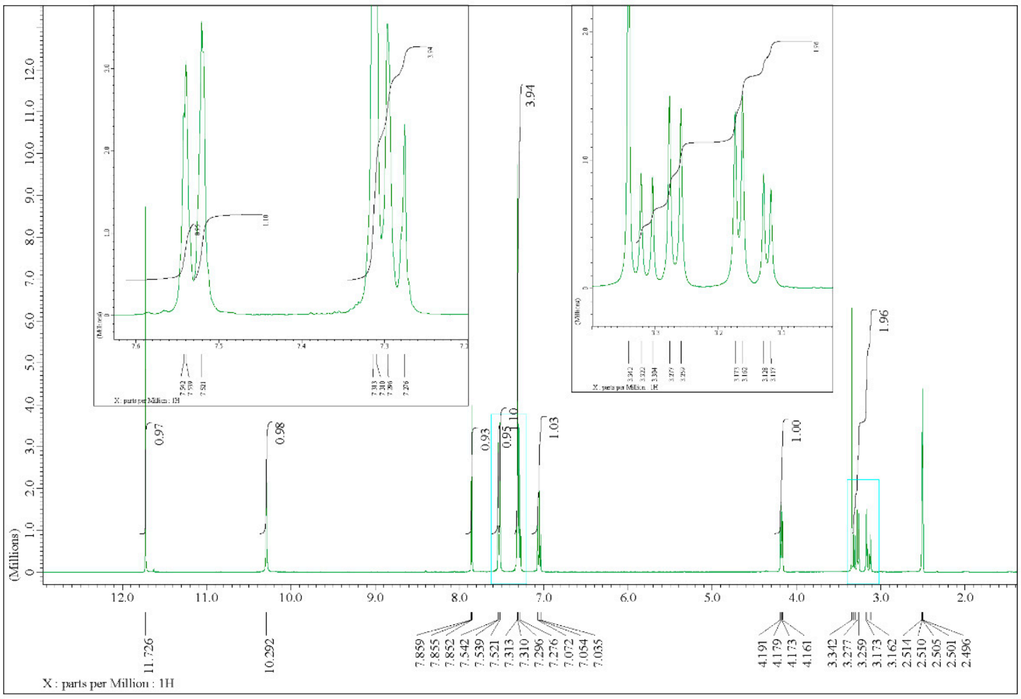

^{1}H NMR of compound 4a

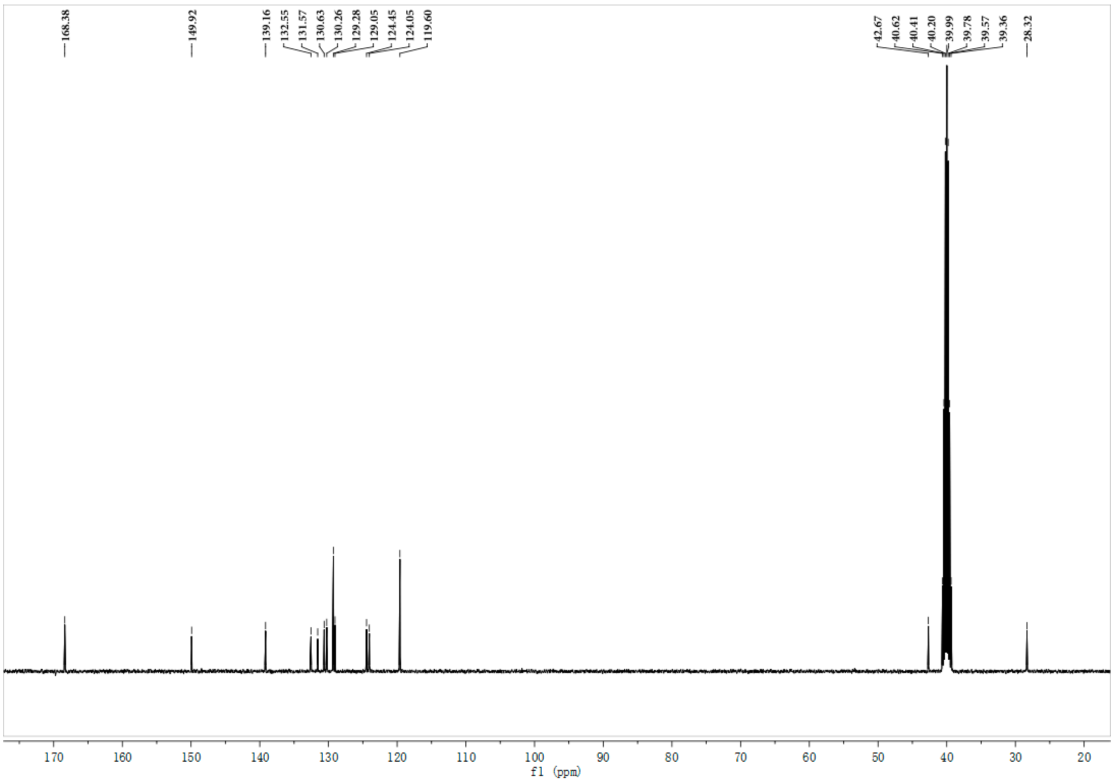

^{13}C NMR of compound 4a

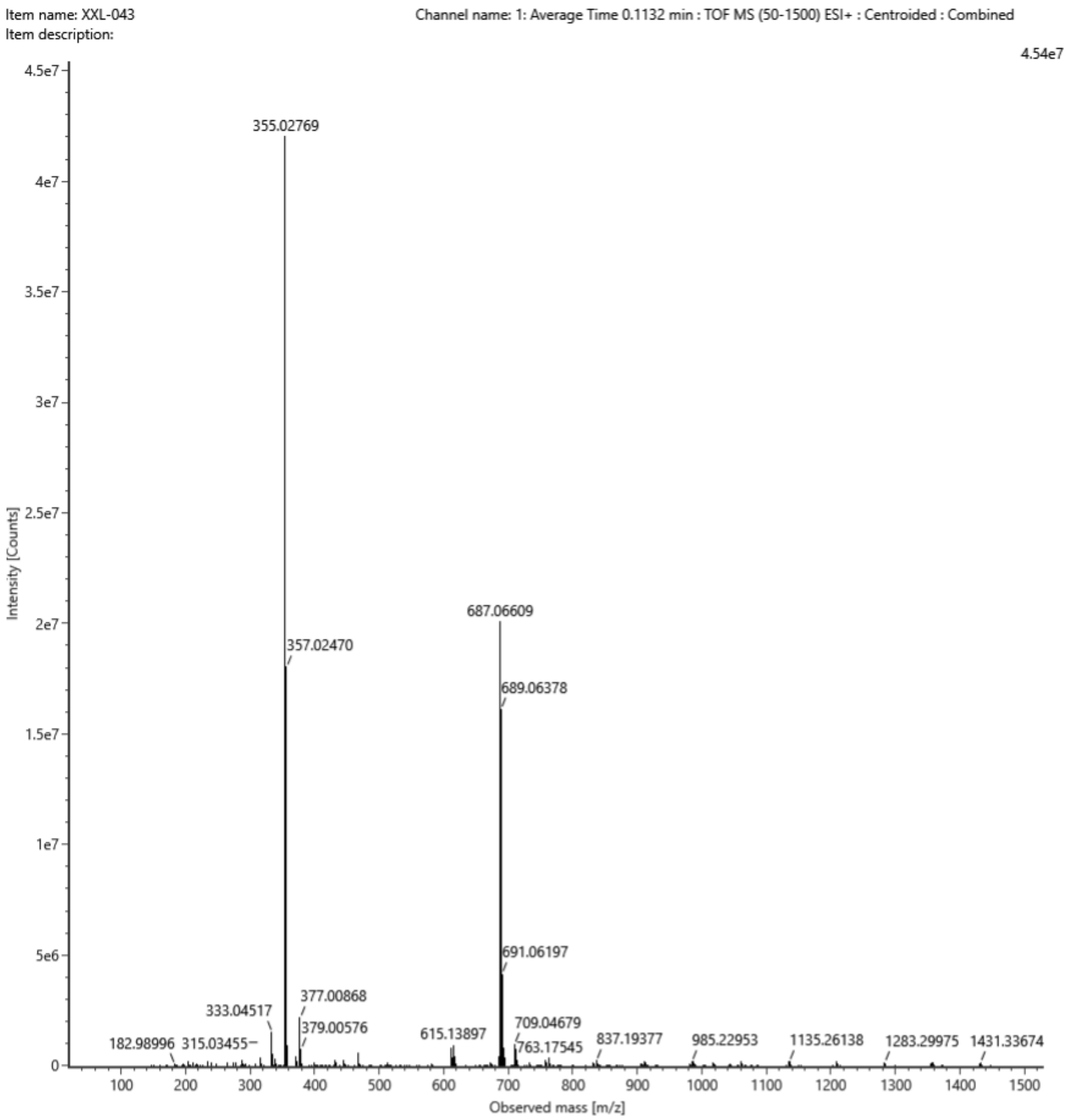

HRMS of compound 4a

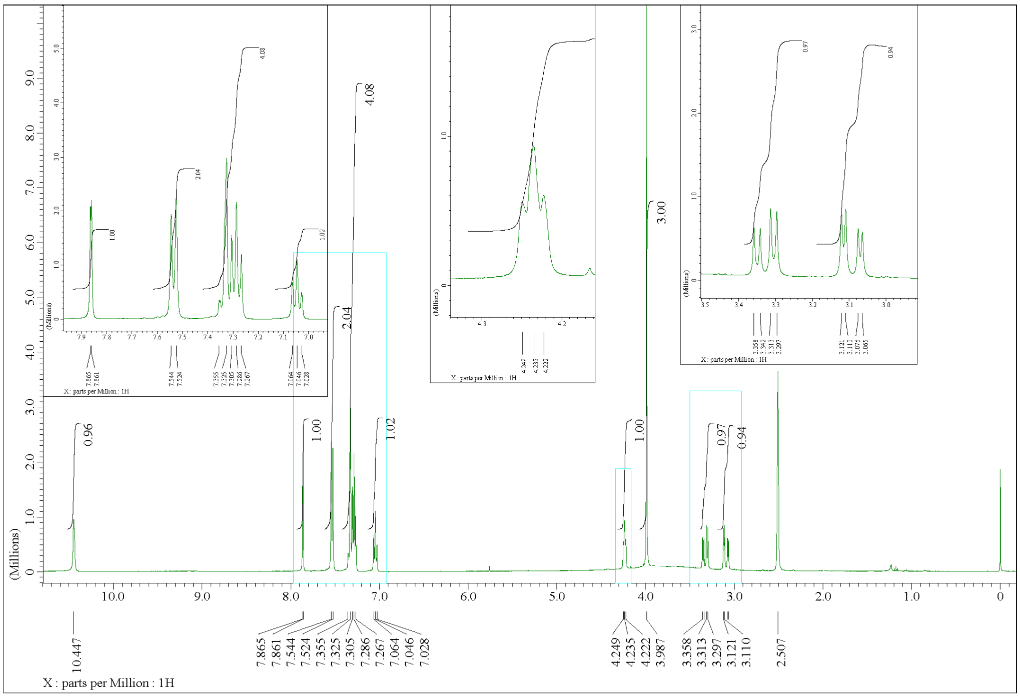

^{1}H NMR of compound 4b

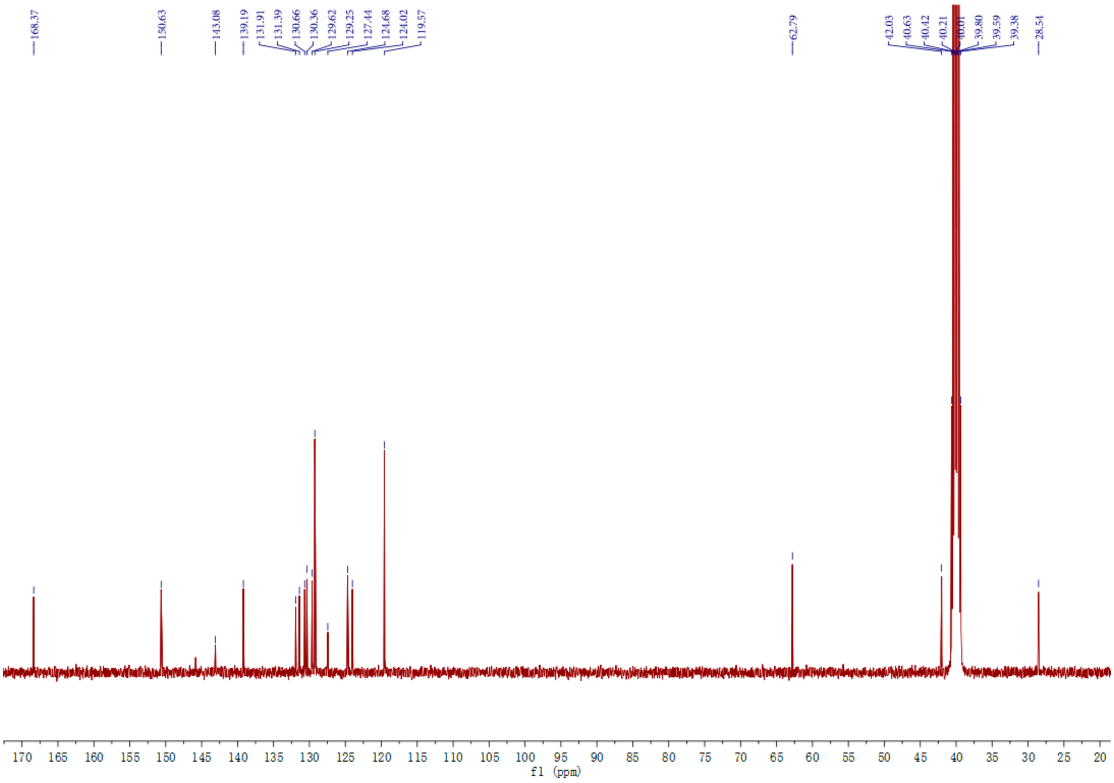

^{13}C NMR of compound 4b

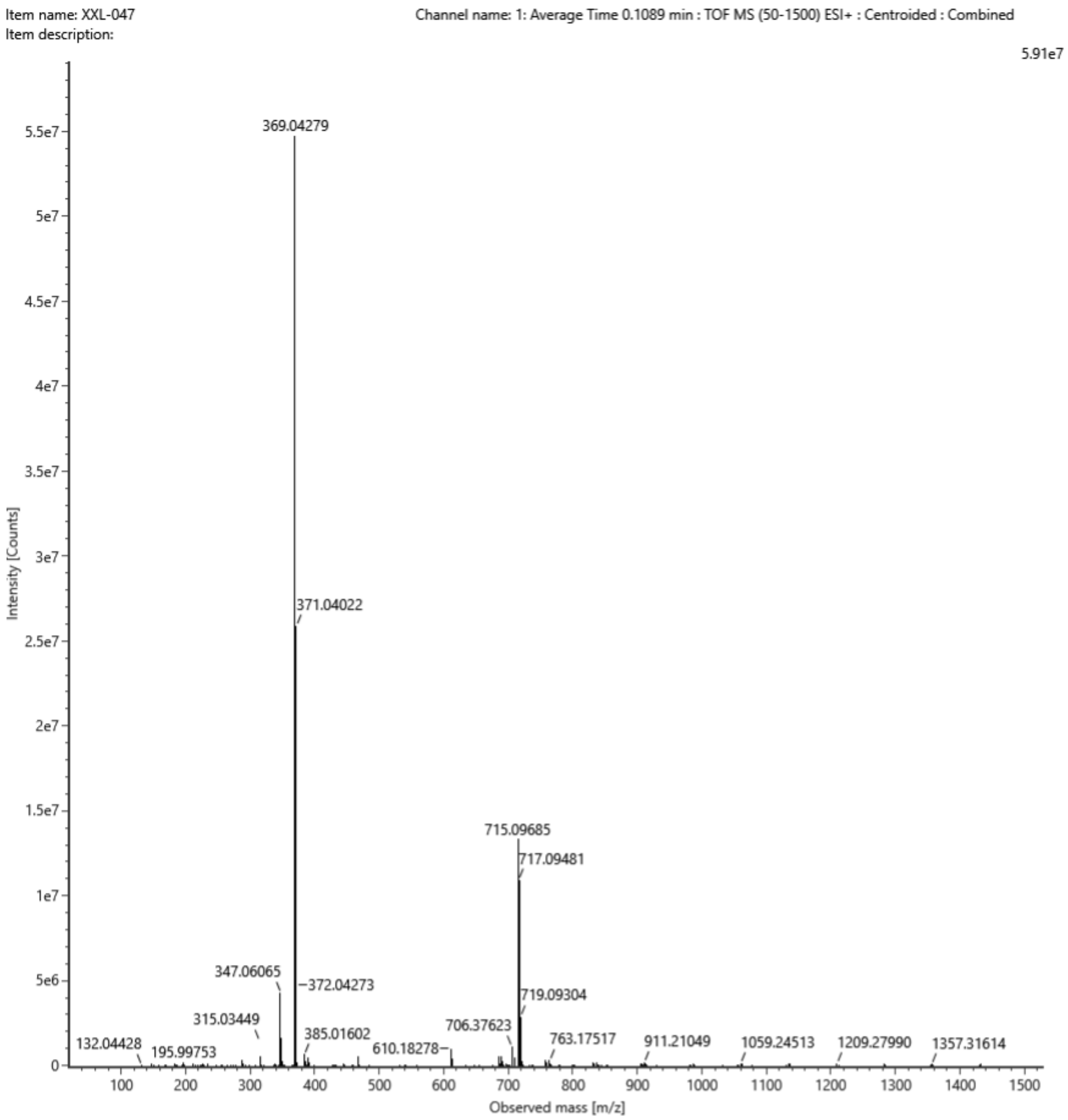

HRMS of compound 4b

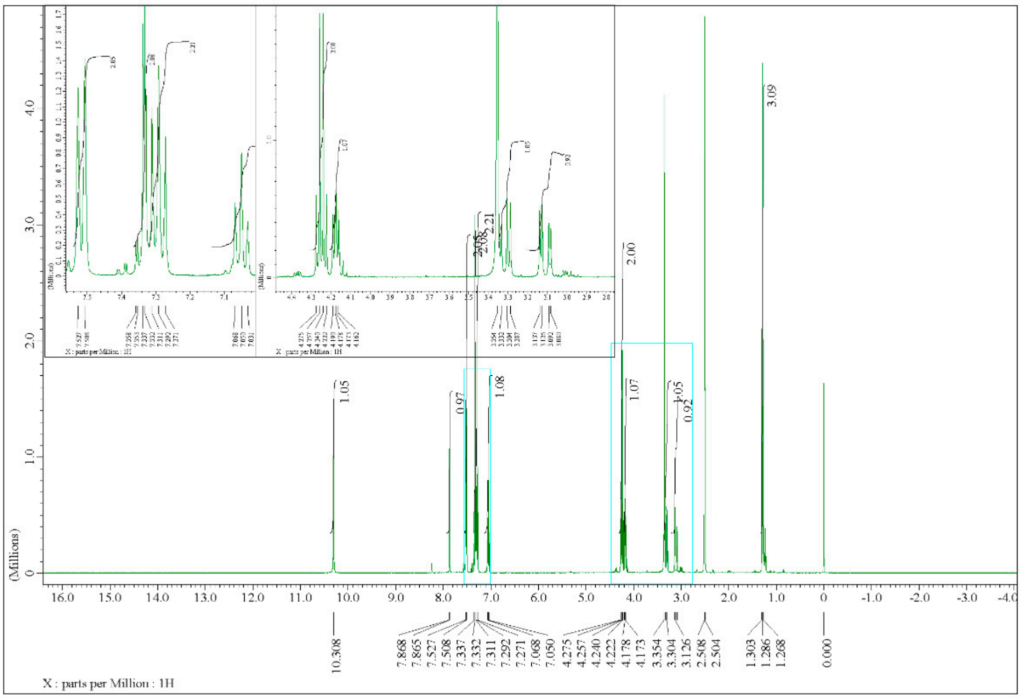

^{1}H NMR of compound 4c

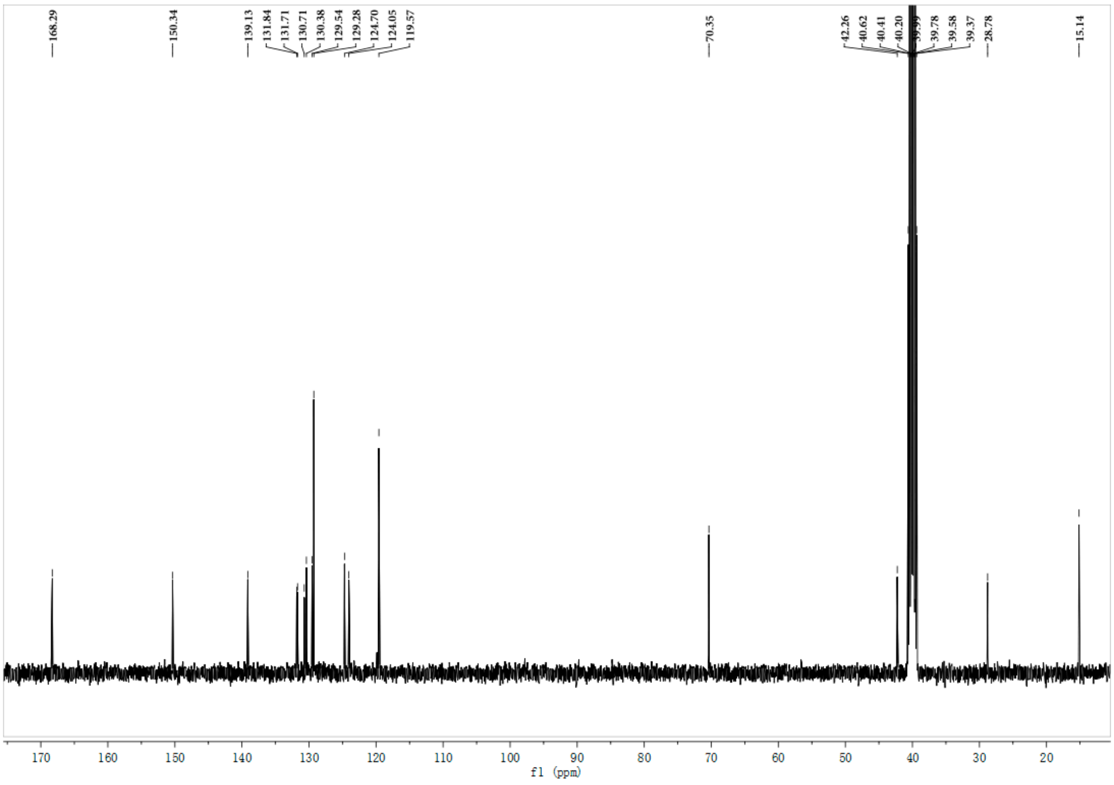

^{13}C NMR of compound 4c

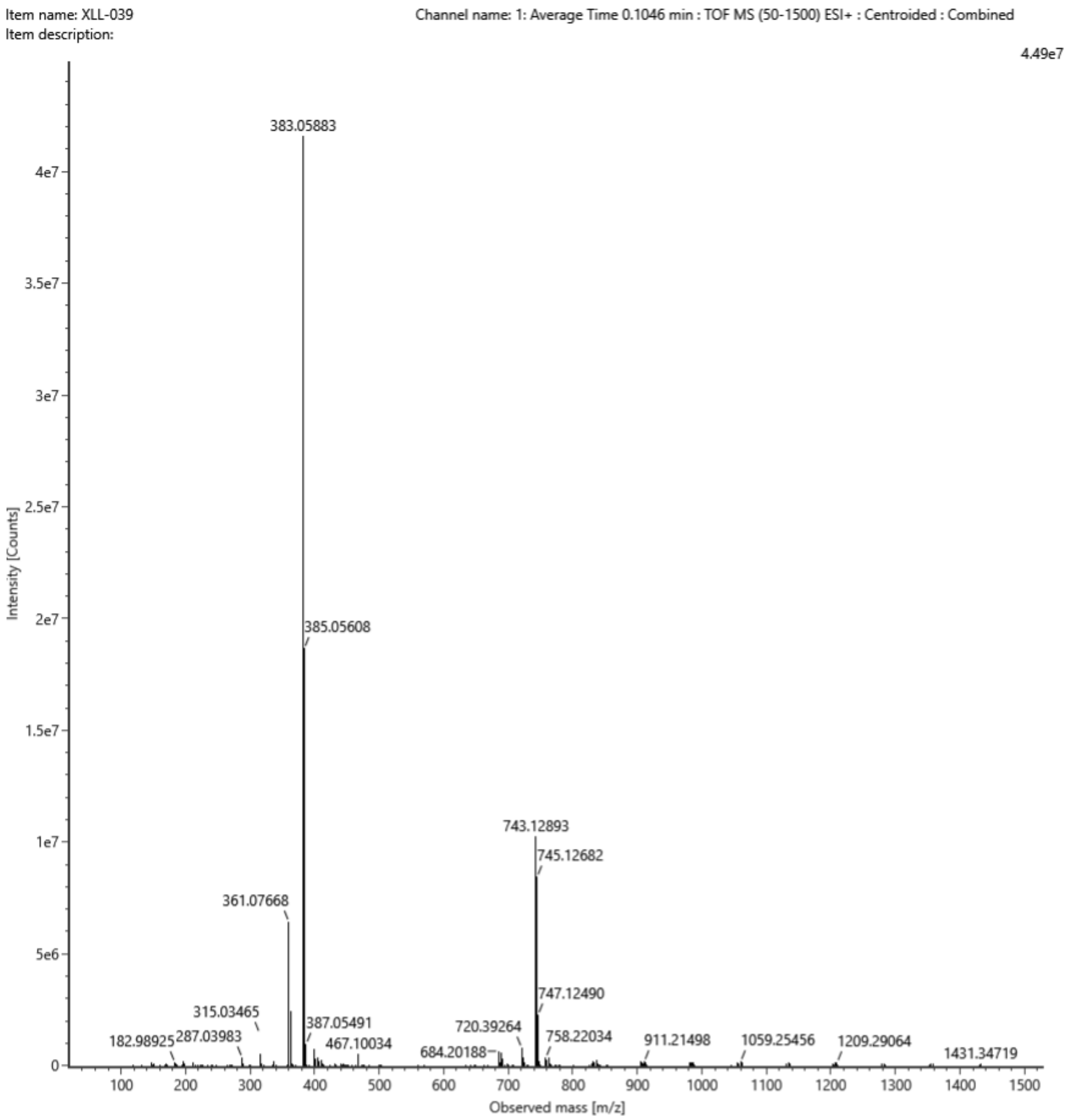

HRMS of compound 4c

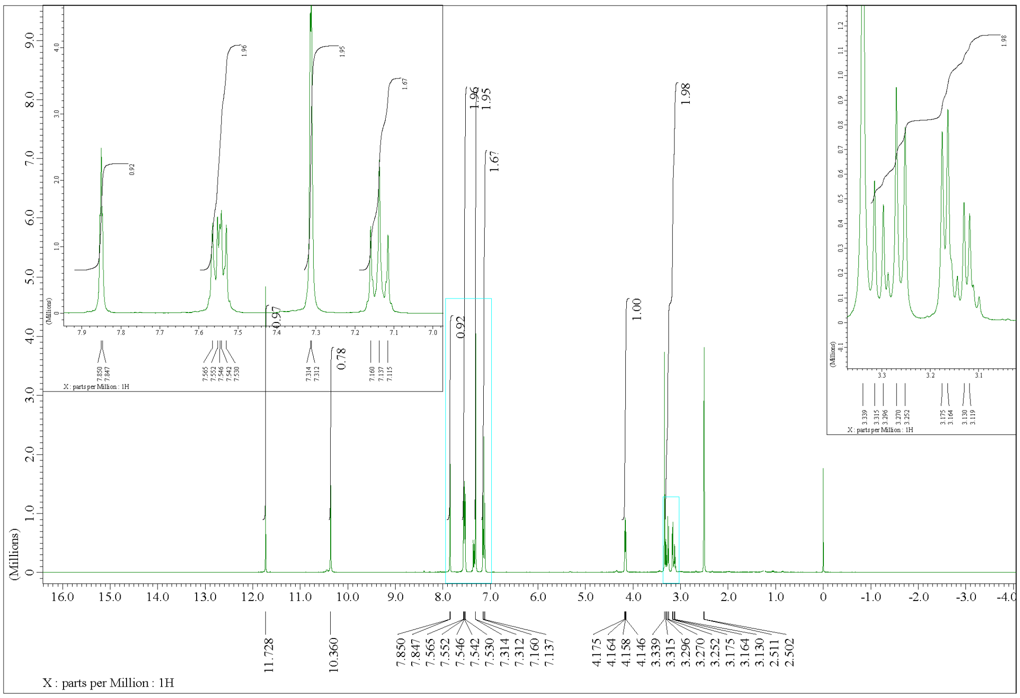

^{1}H NMR of compound 4d

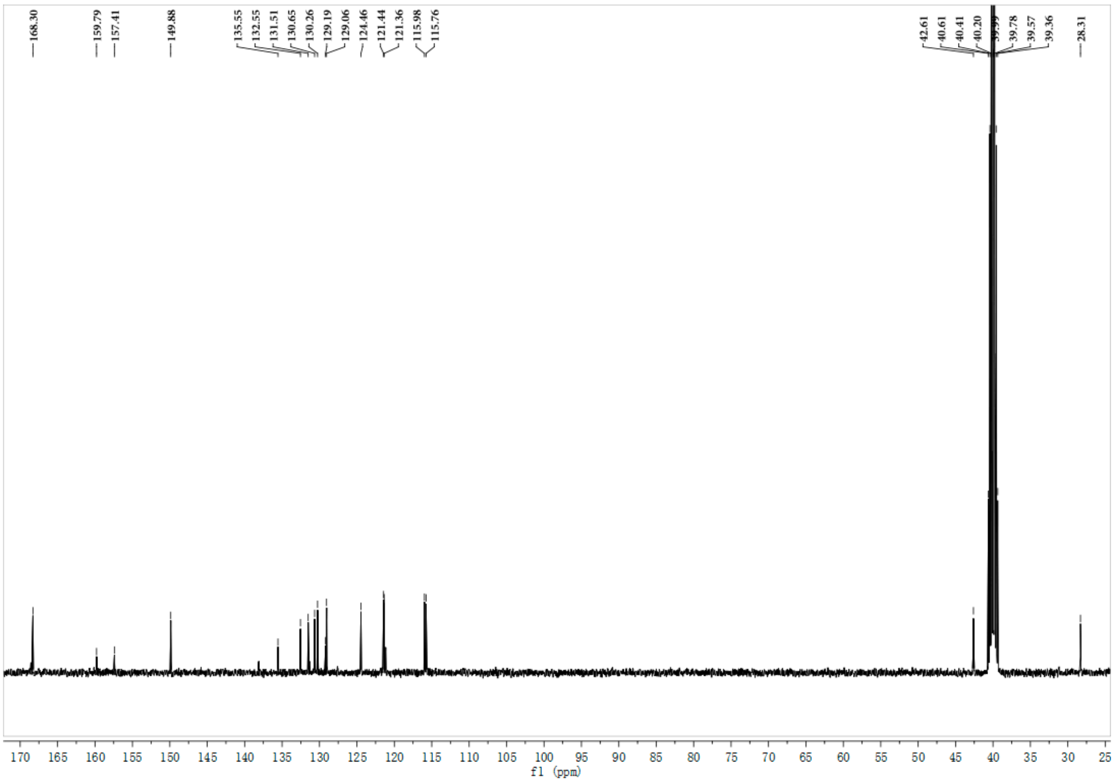

^{13}C NMR of compound 4d

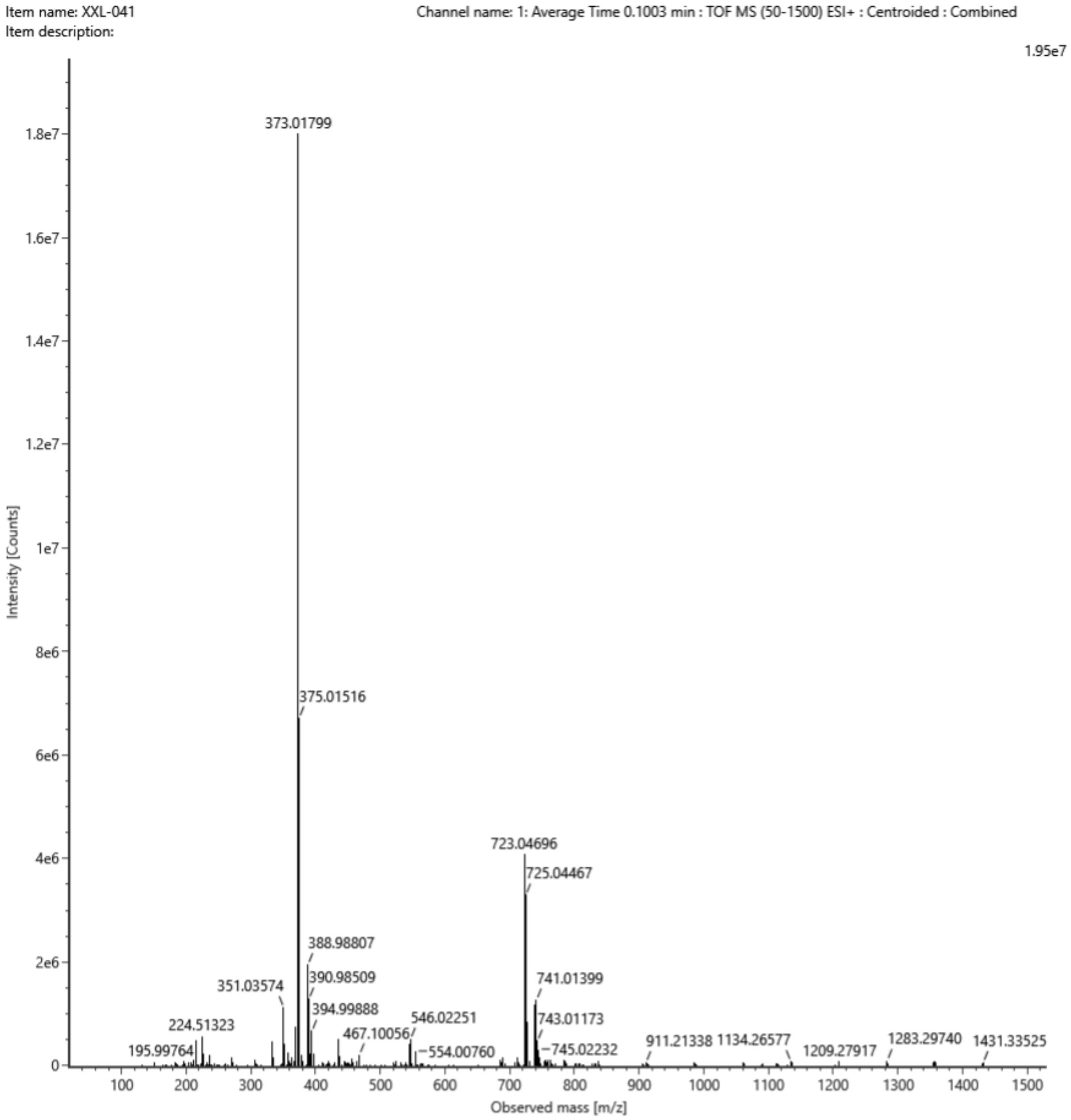

HRMS of compound 4d

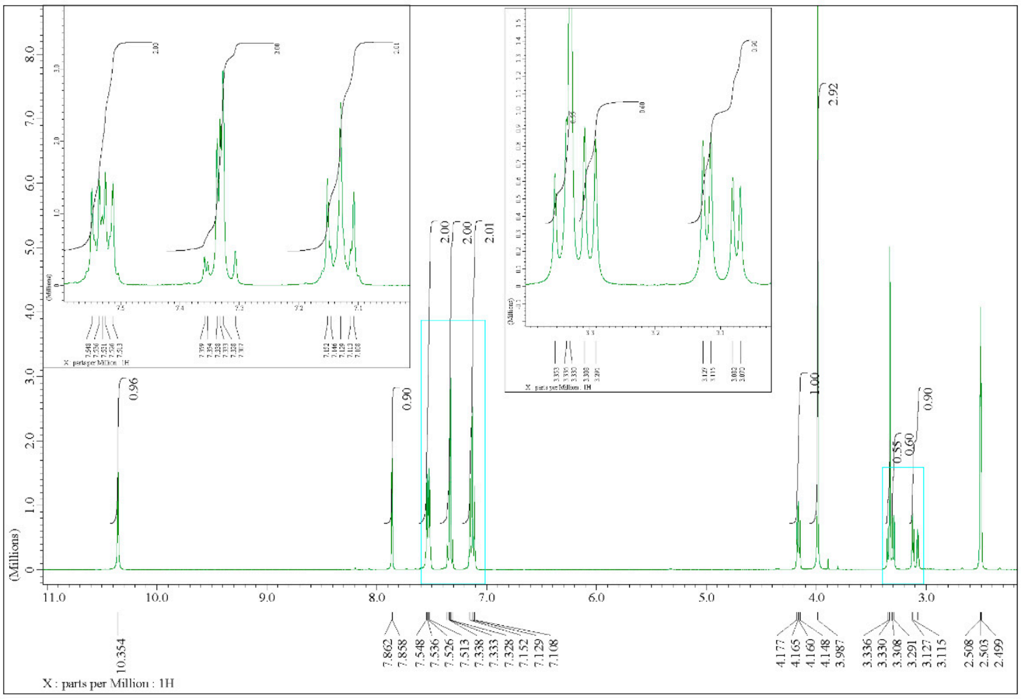

^1H NMR of compound 4e

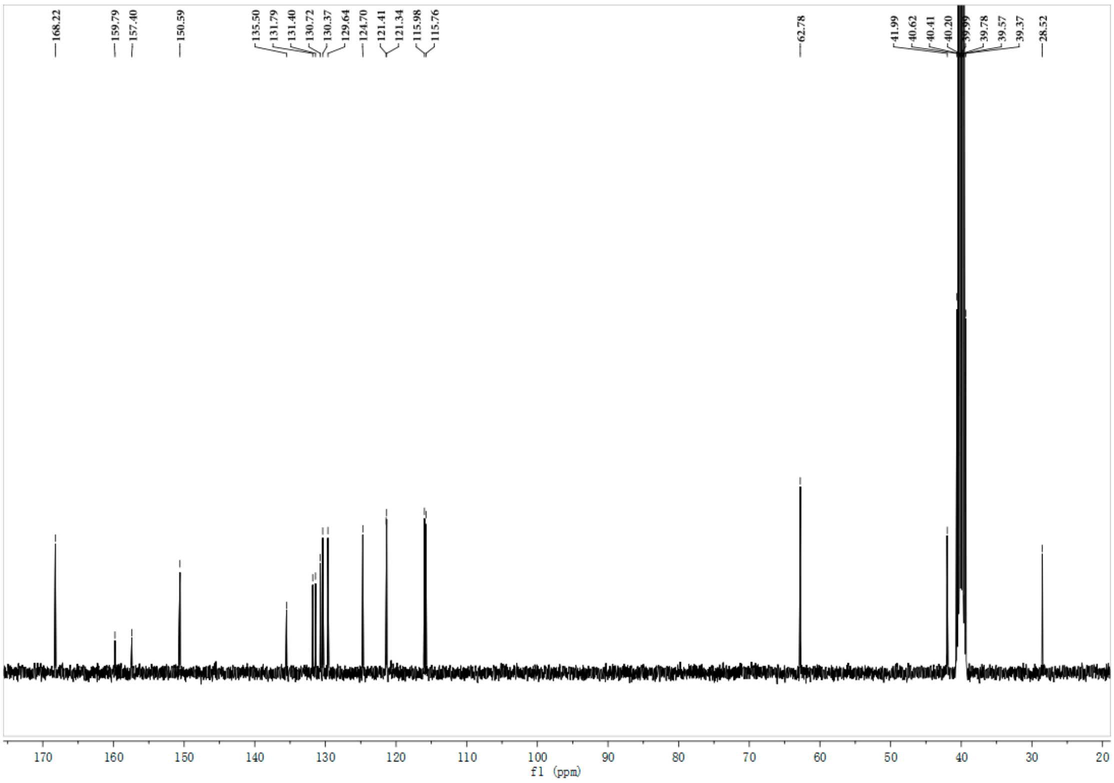

^{13}C NMR of compound 4e

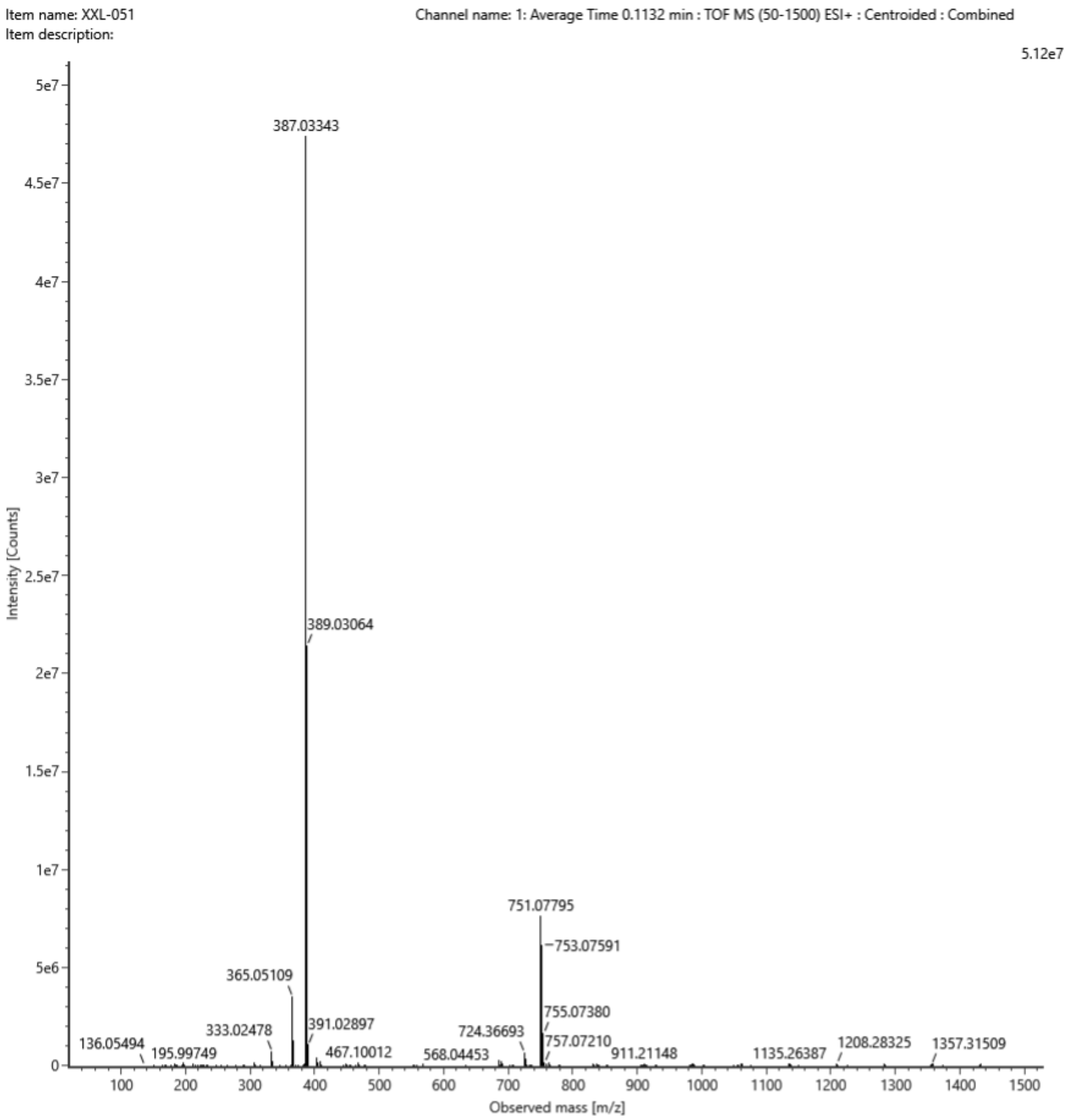

HRMS of compound 4e

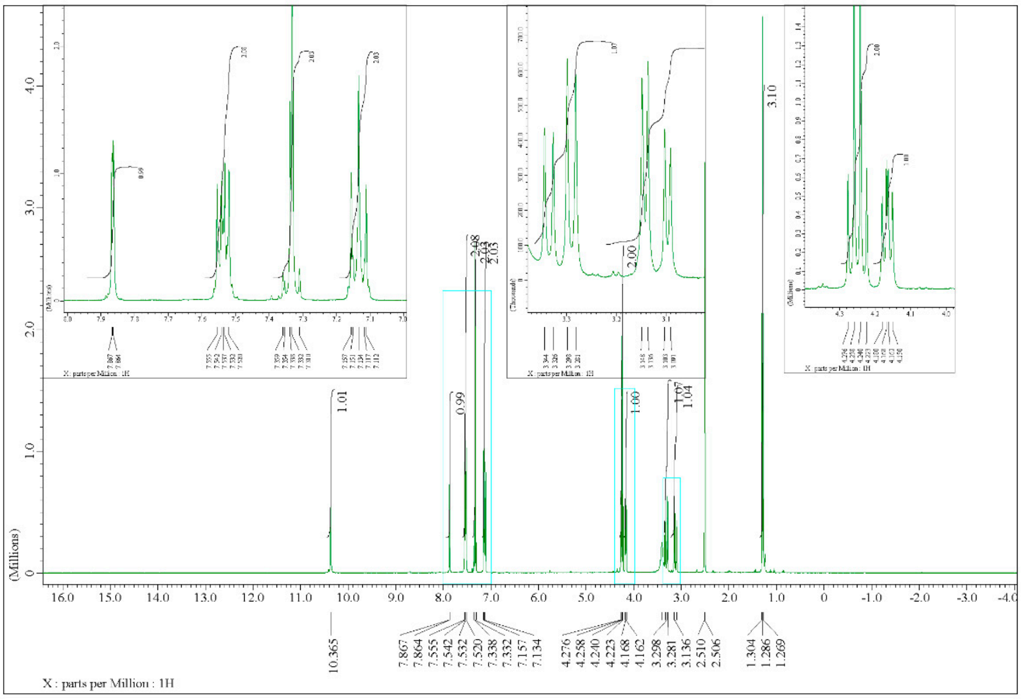

^{1}H NMR of compound 4f

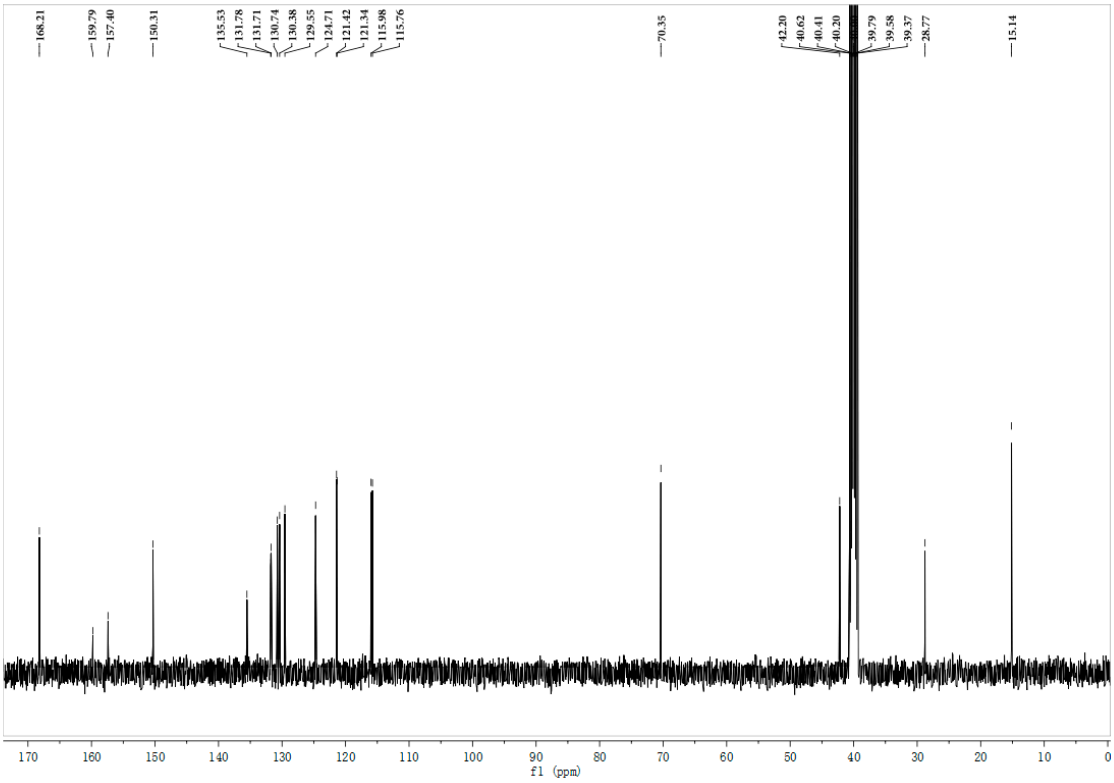

^{13}C NMR of compound 4f

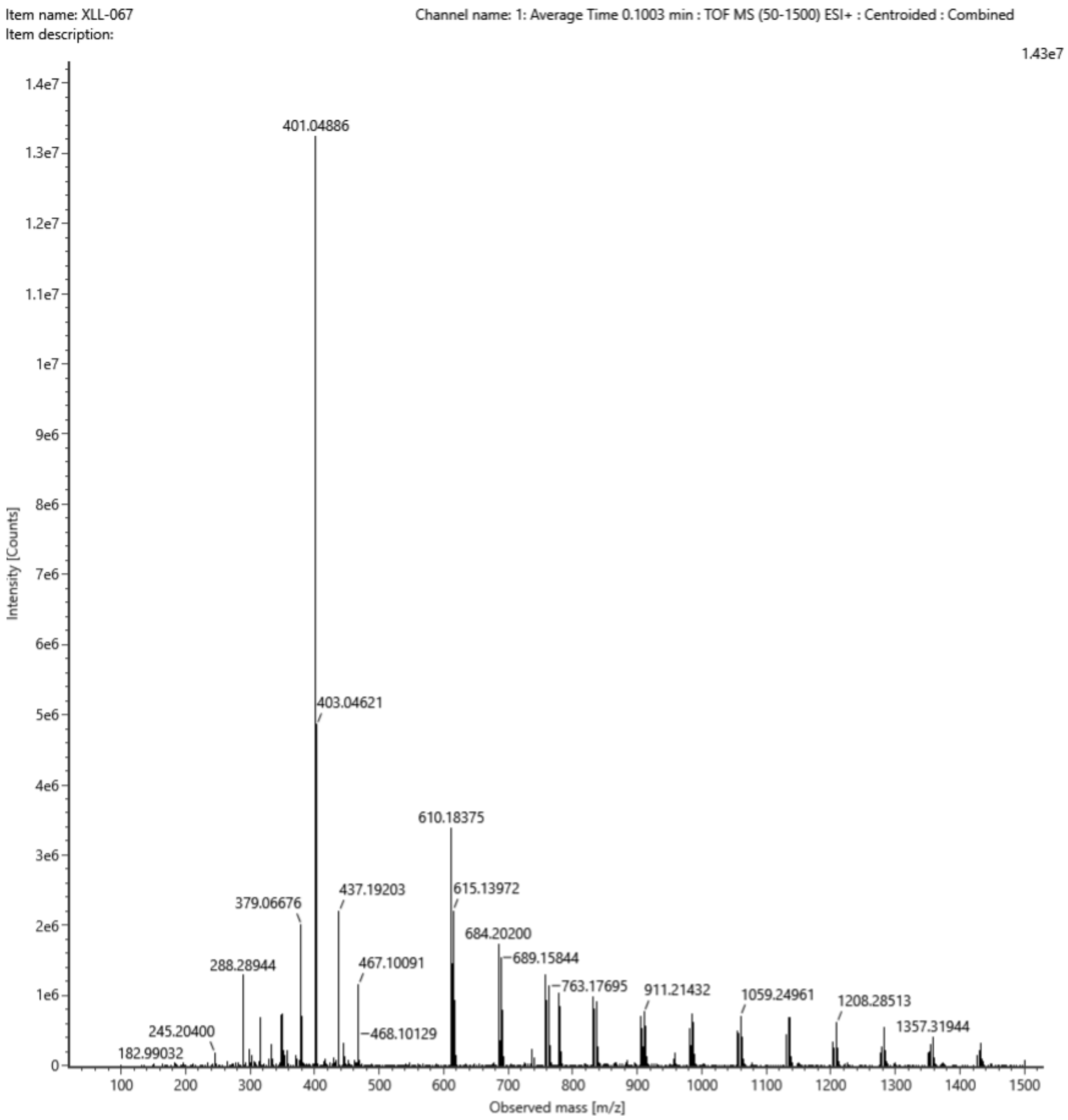

HRMS of compound 4f

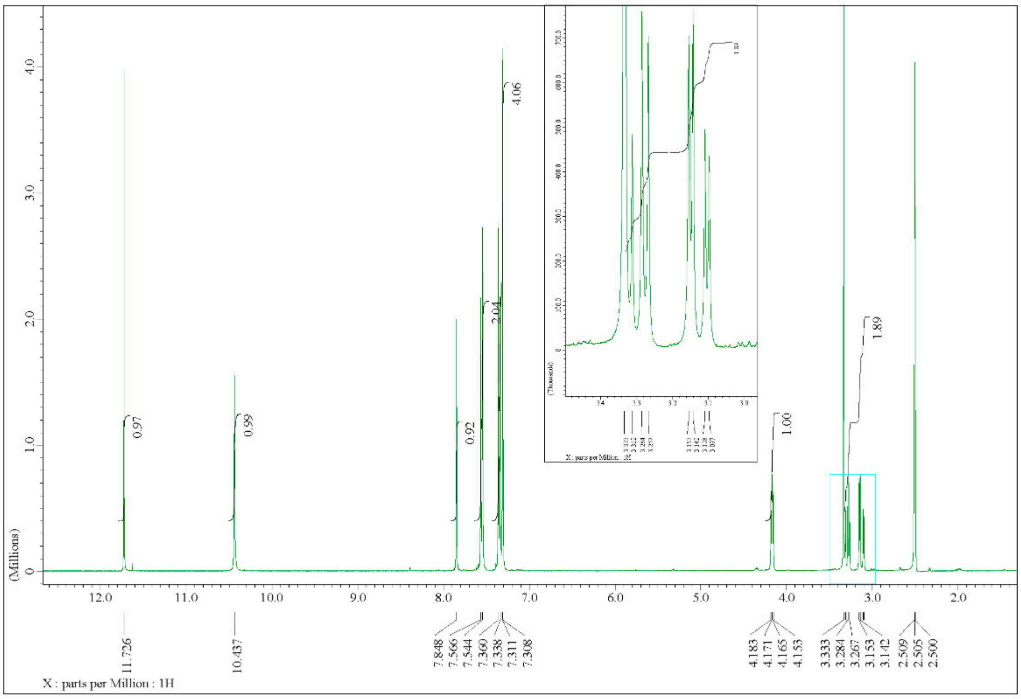

^{1}H NMR of compound 4g

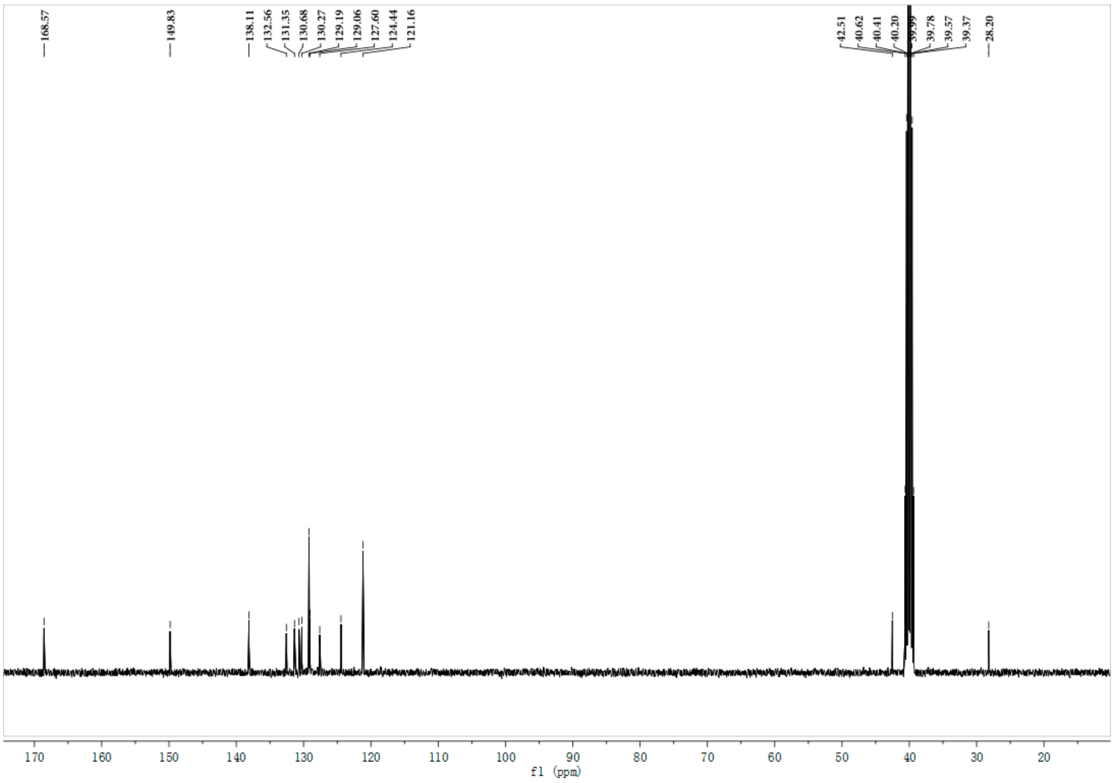

^{13}C NMR of compound 4g

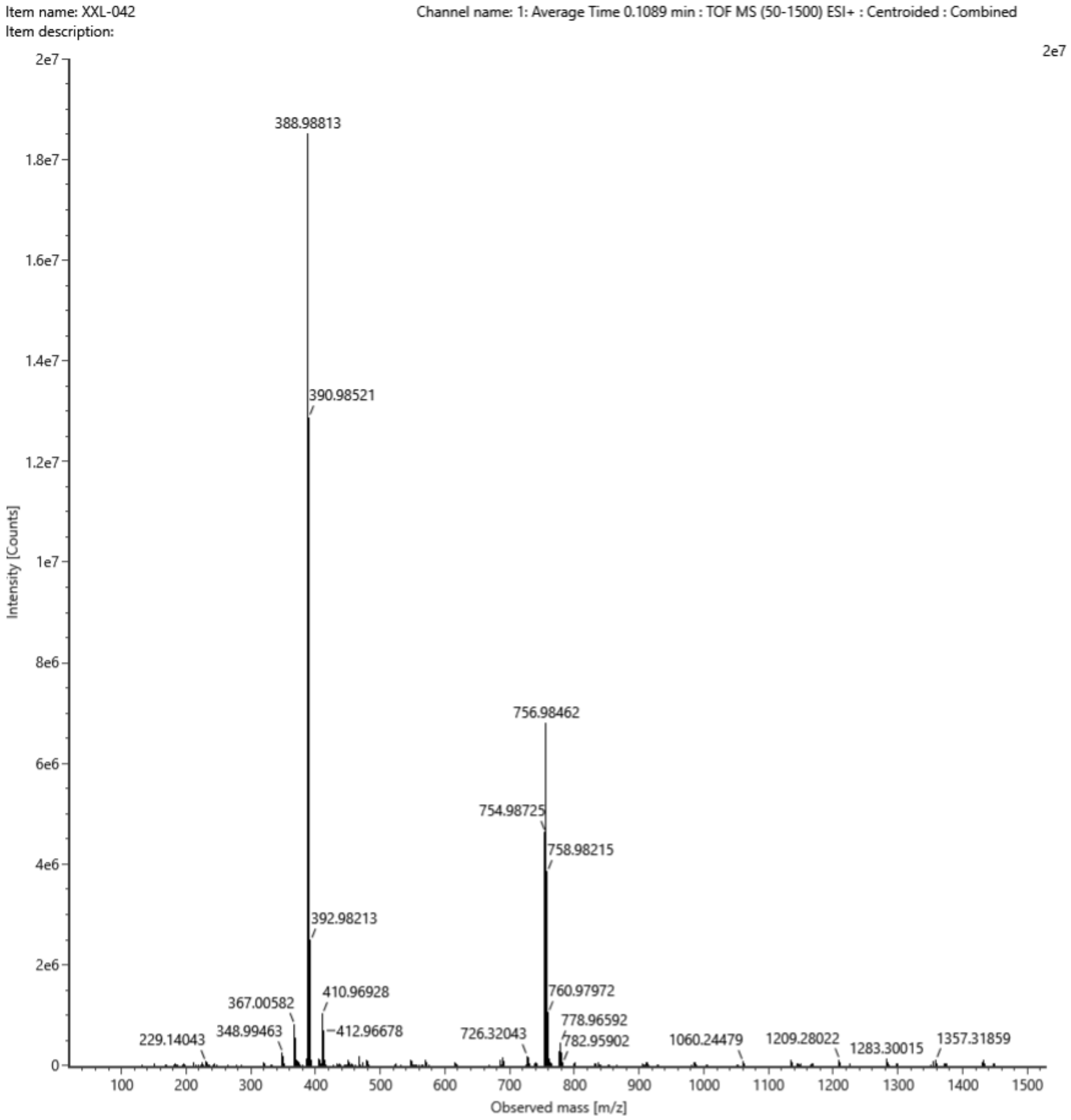

HRMS of compound 4g

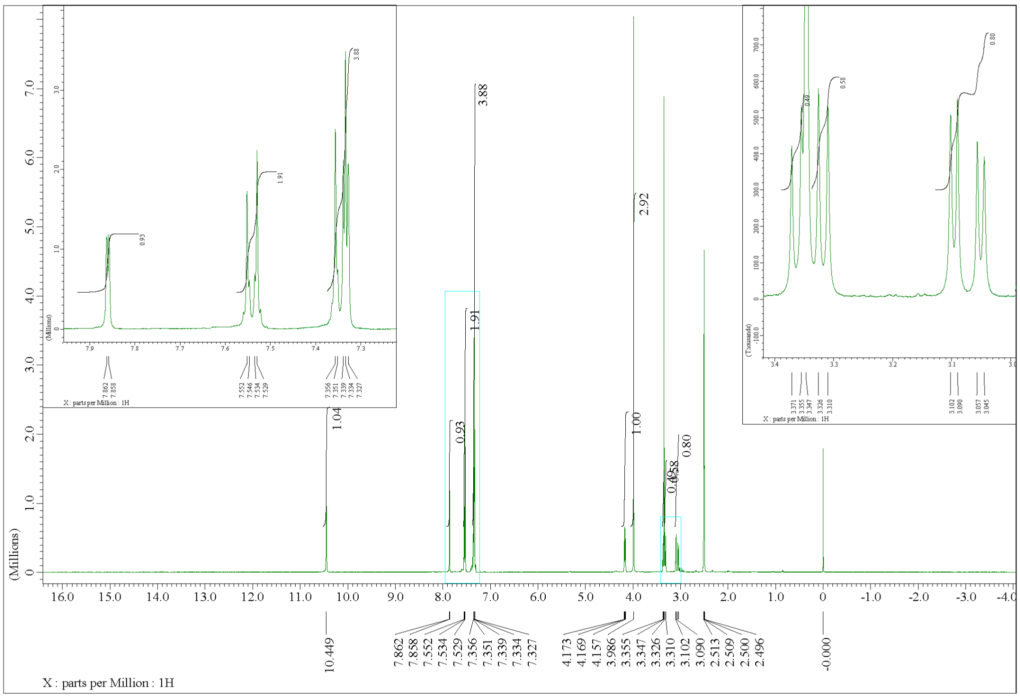

^{1}H NMR of compound 4h

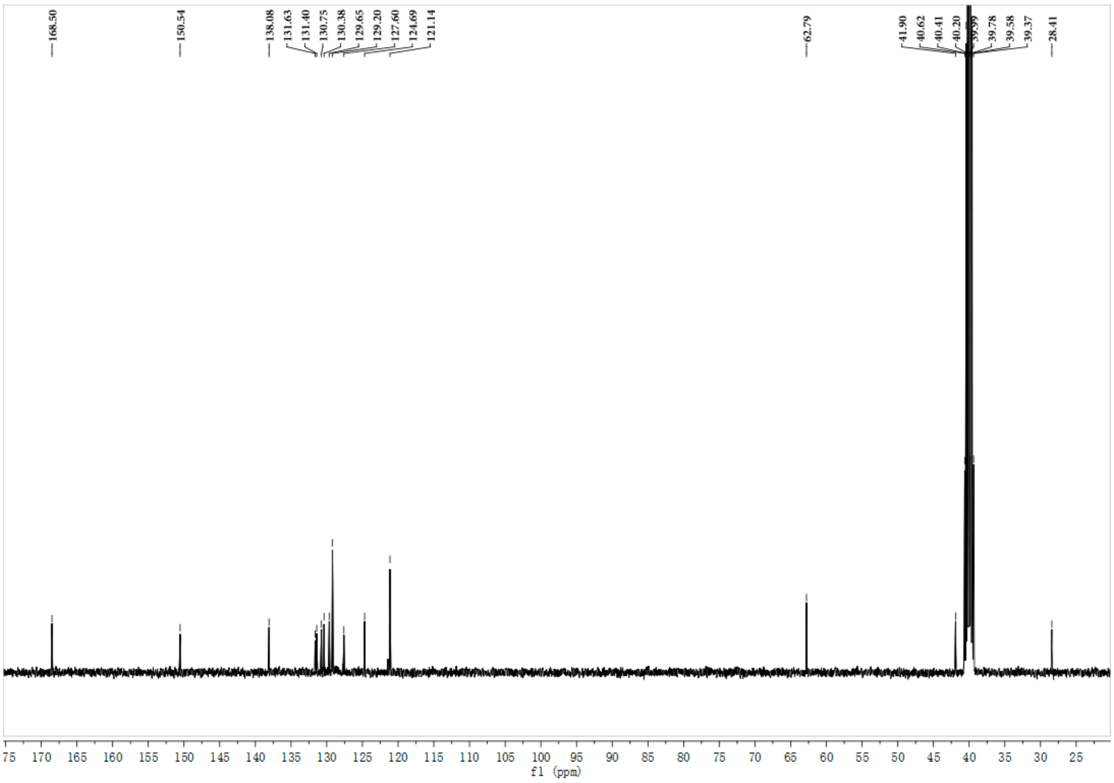

^{13}C NMR of compound 4h

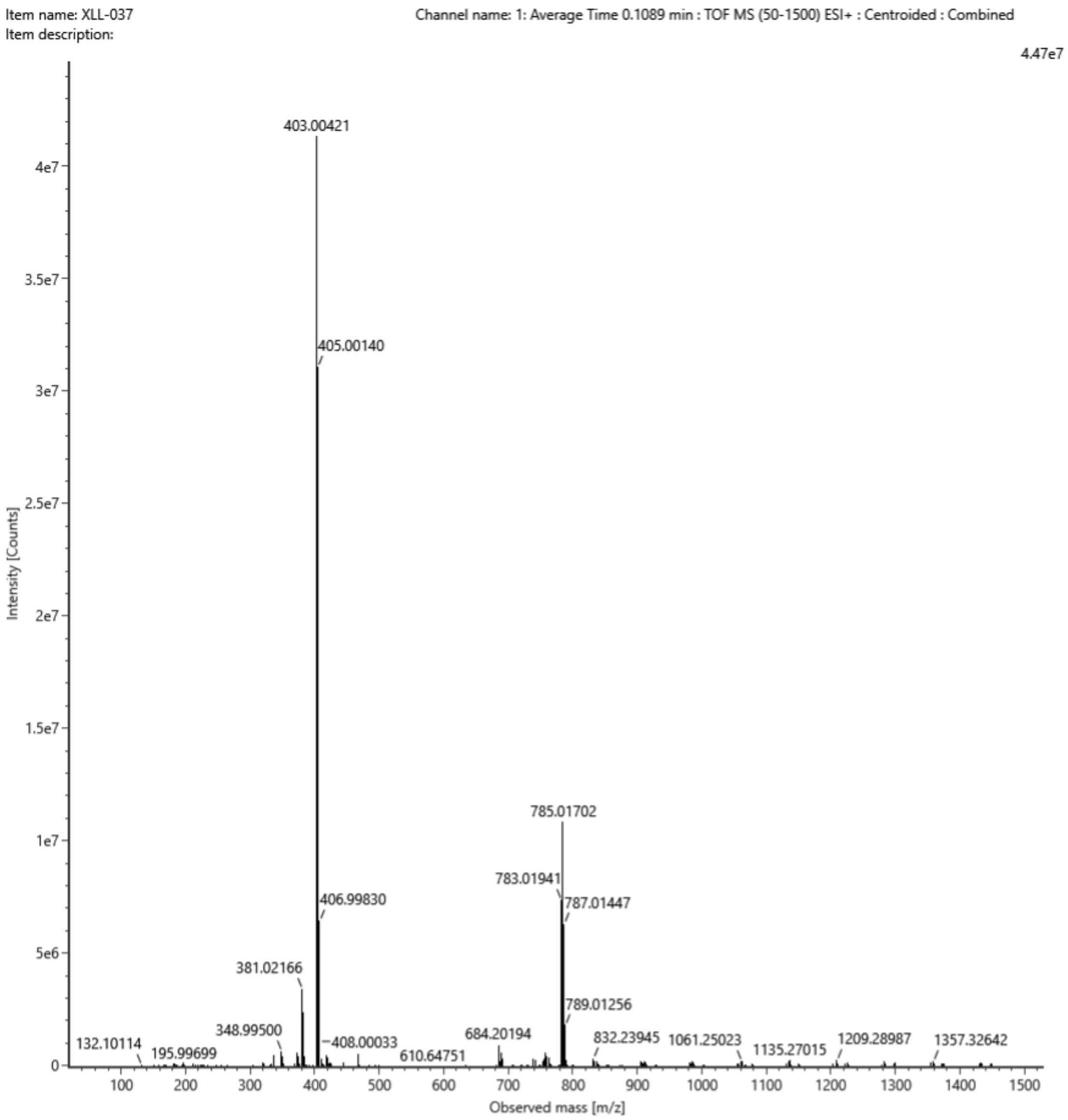

HRMS of compound 4h

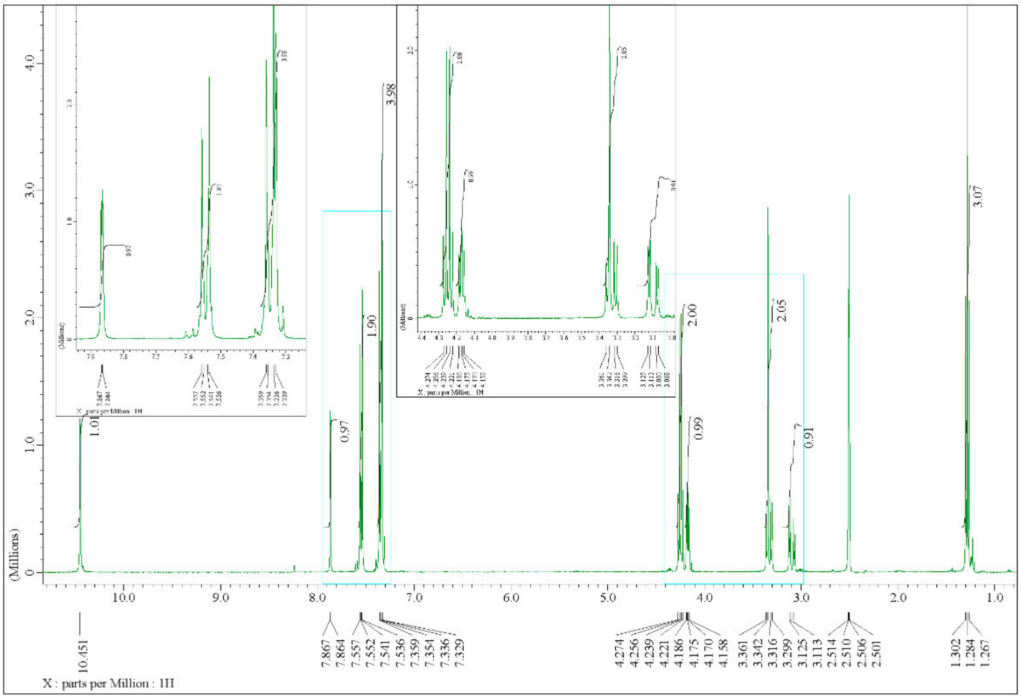

^{1}H NMR of compound 4i

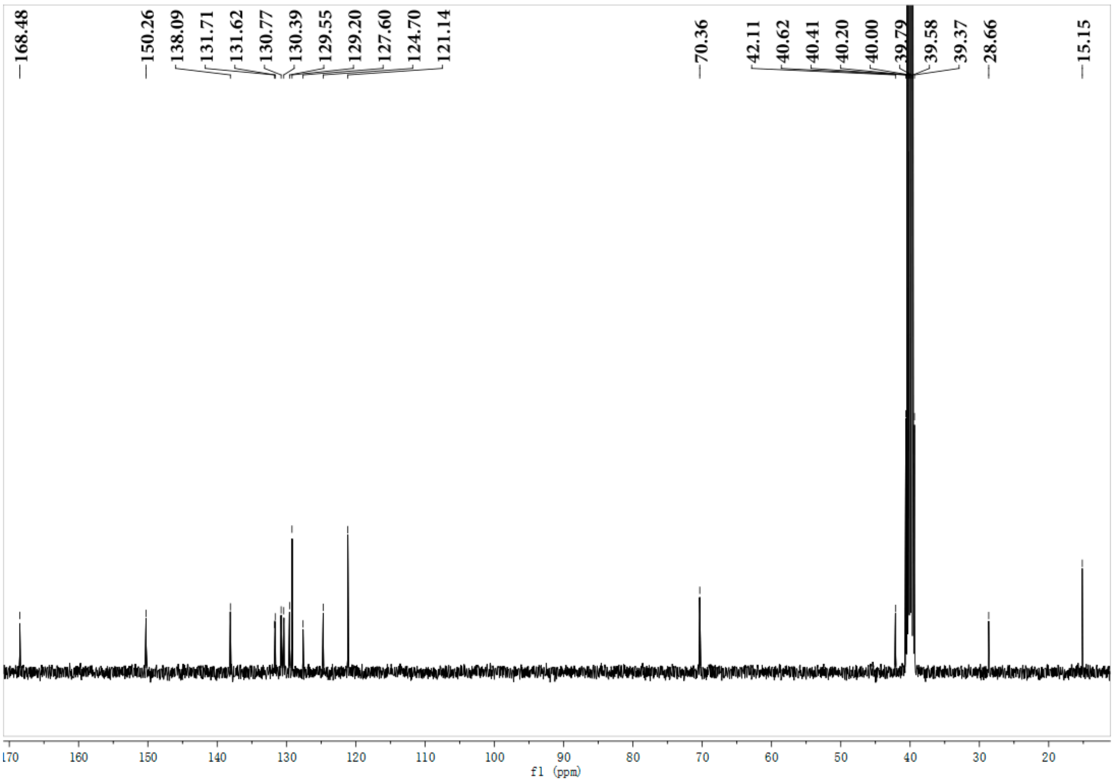

^{13}C NMR of compound 4i

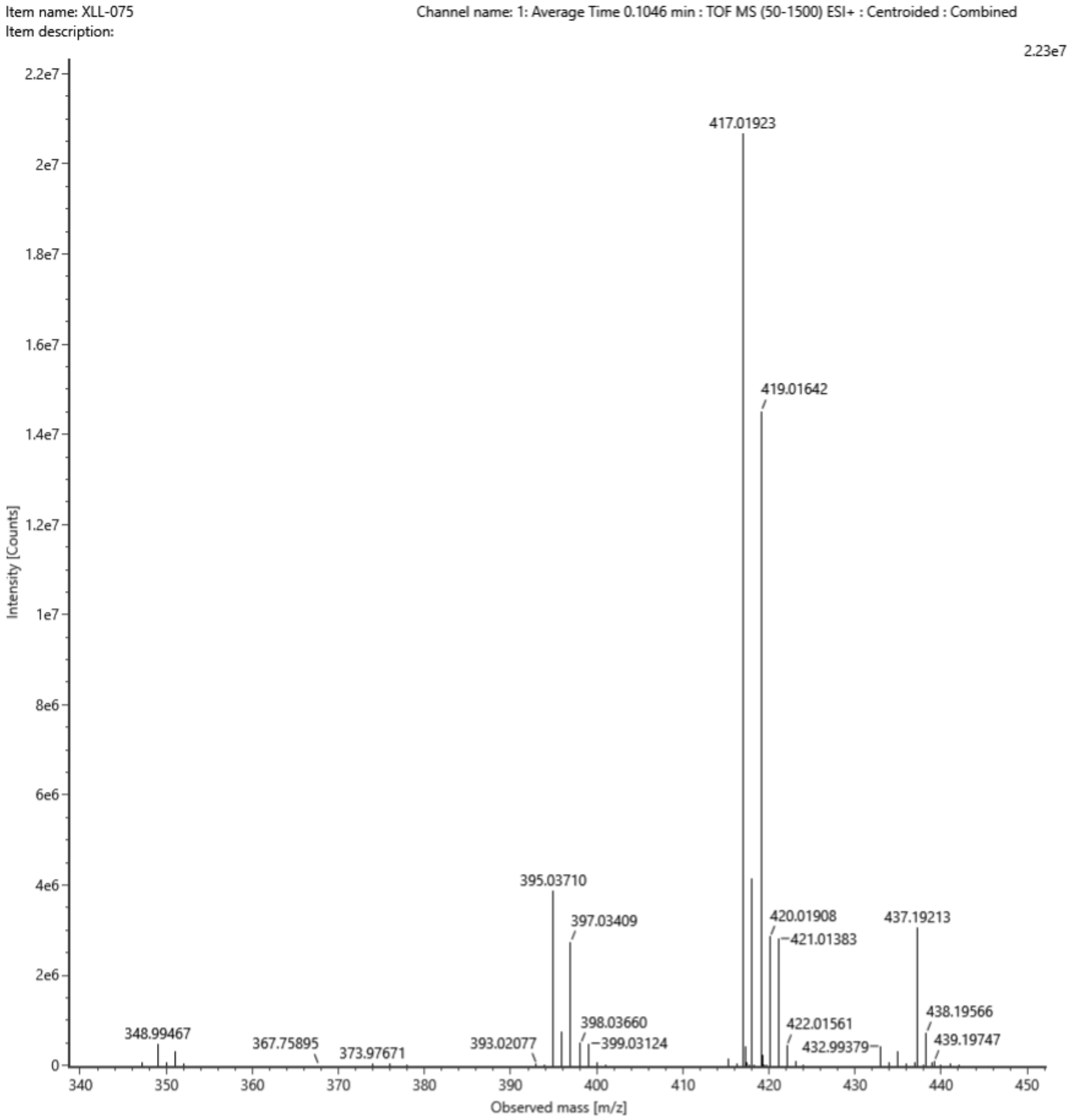

HRMS of compound 4i

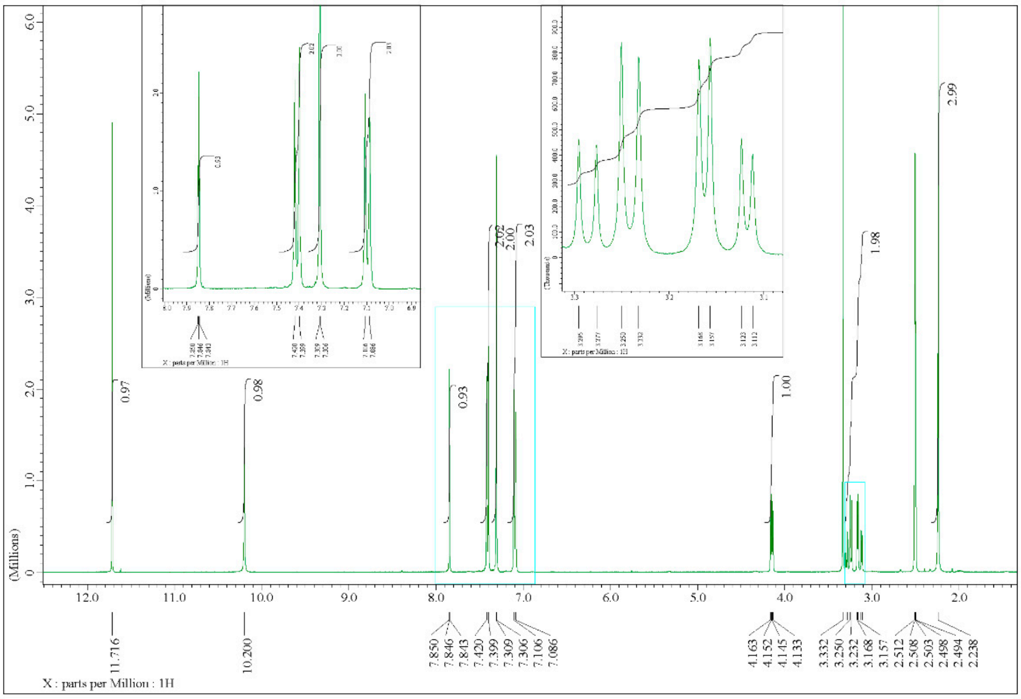

^{1}H NMR of compound 4j

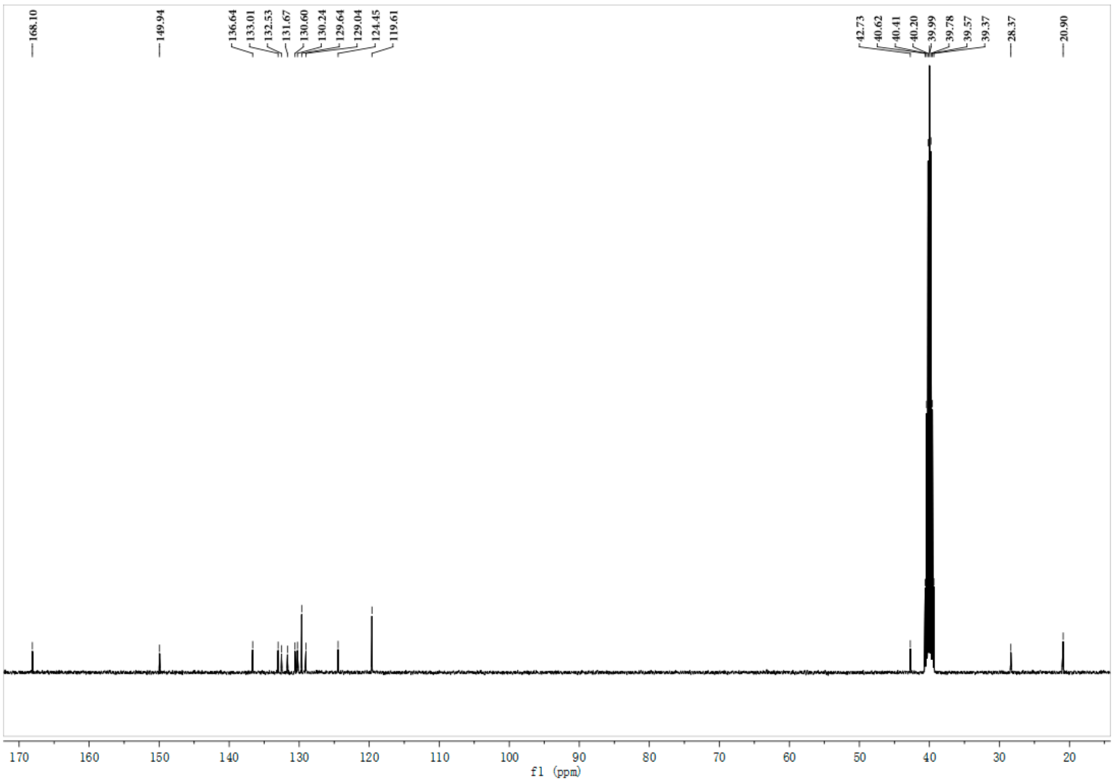

^{13}C NMR of compound 4j

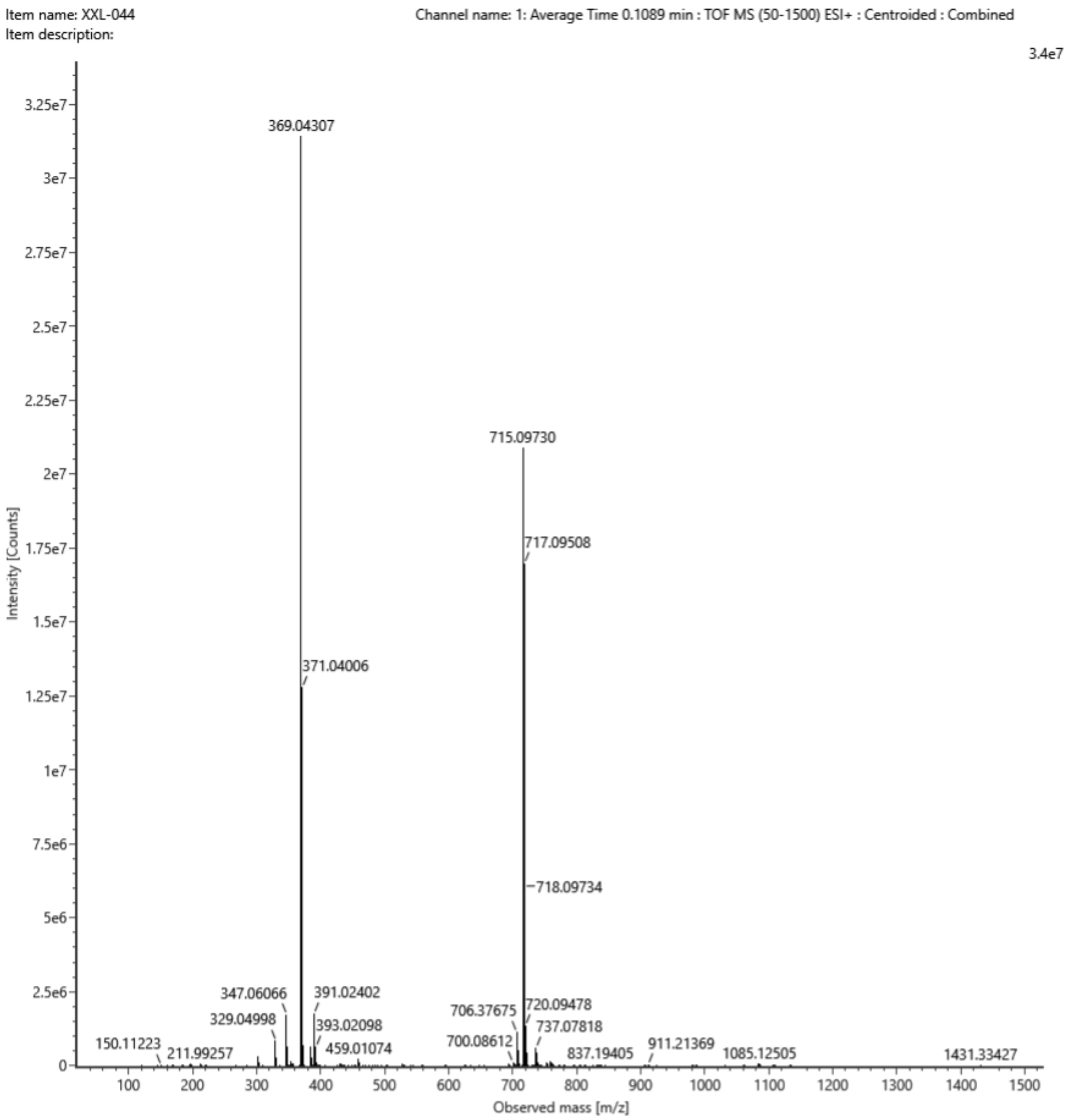

HRMS of compound 4j

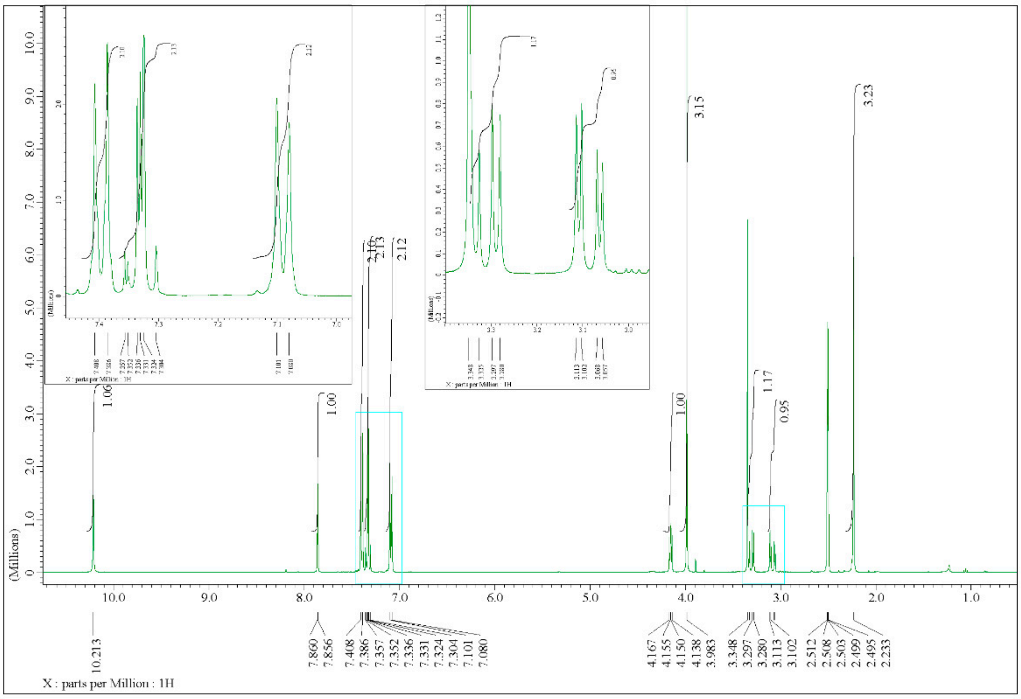

^{1}H NMR of compound 4k

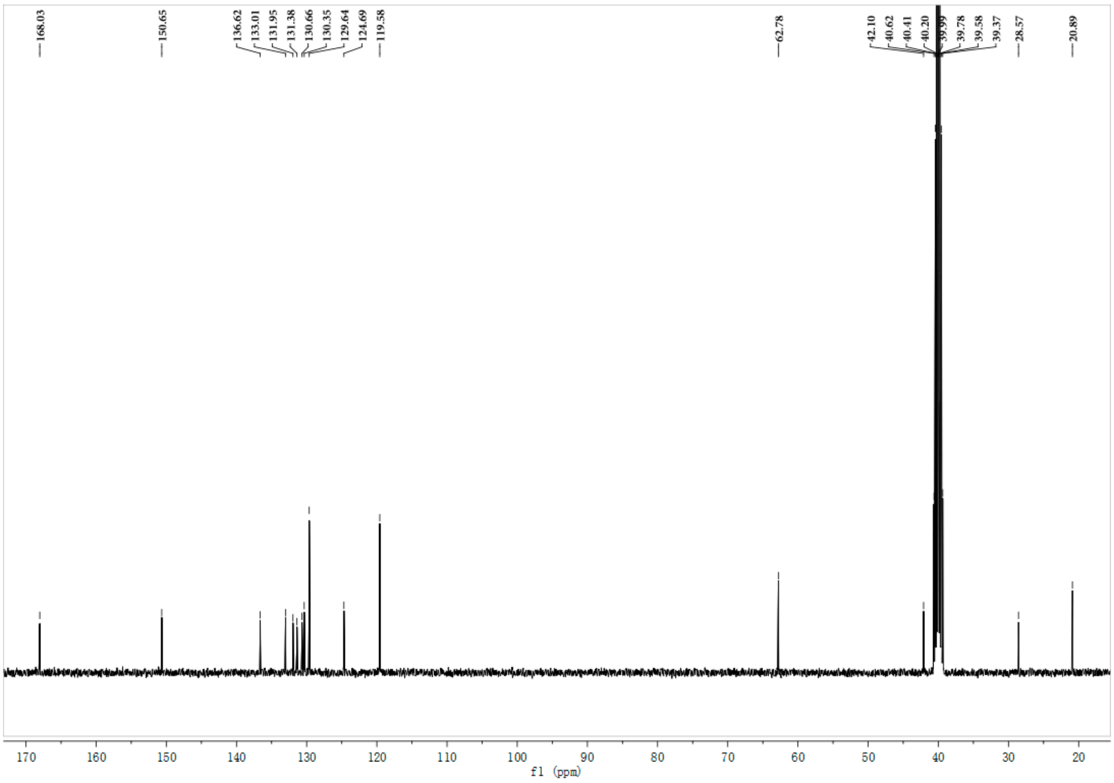

^{13}C NMR of compound 4k

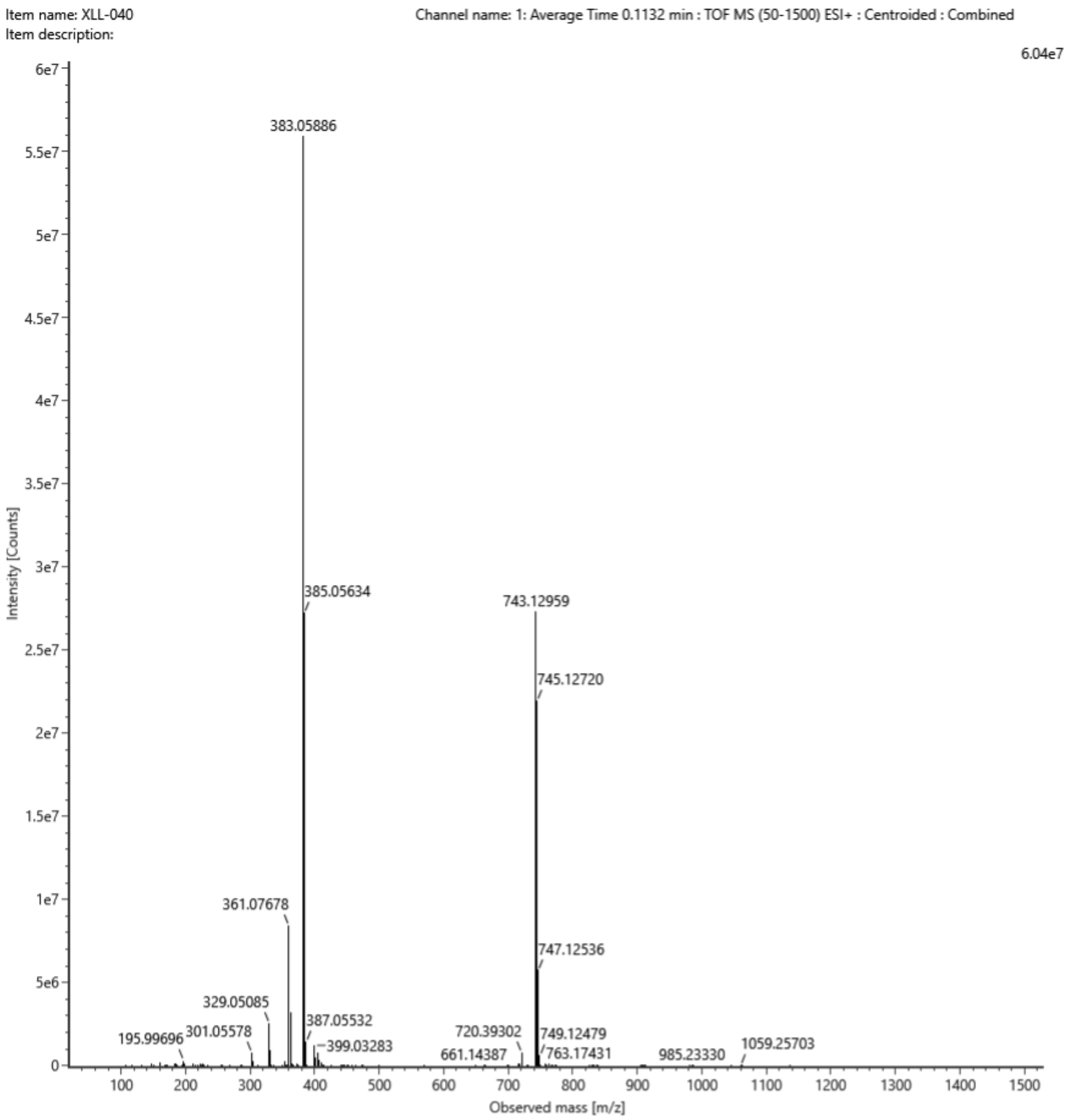

HRMS of compound 4k

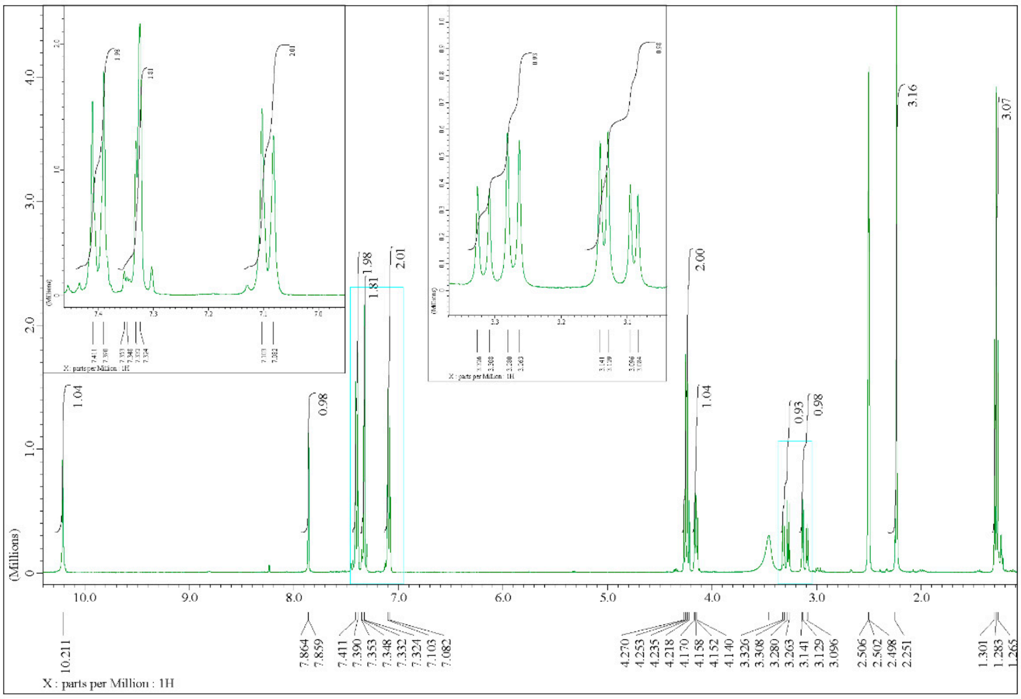

^{1}H NMR of compound 4l

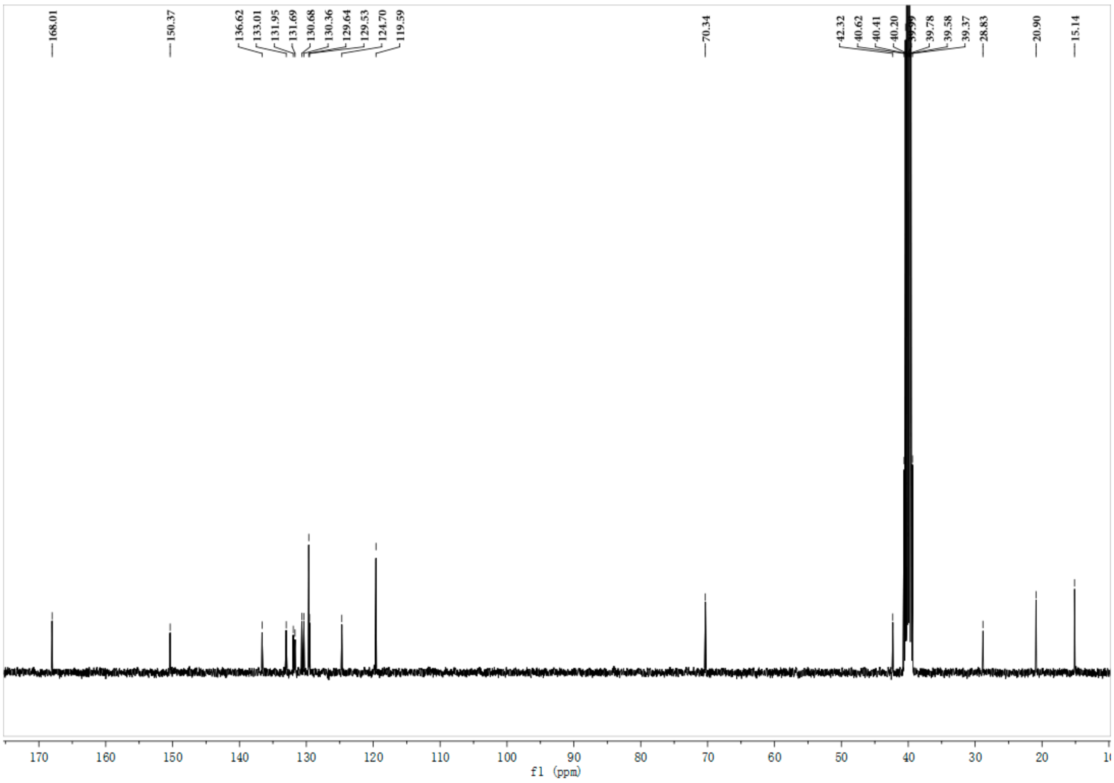

^{13}C NMR of compound 4l

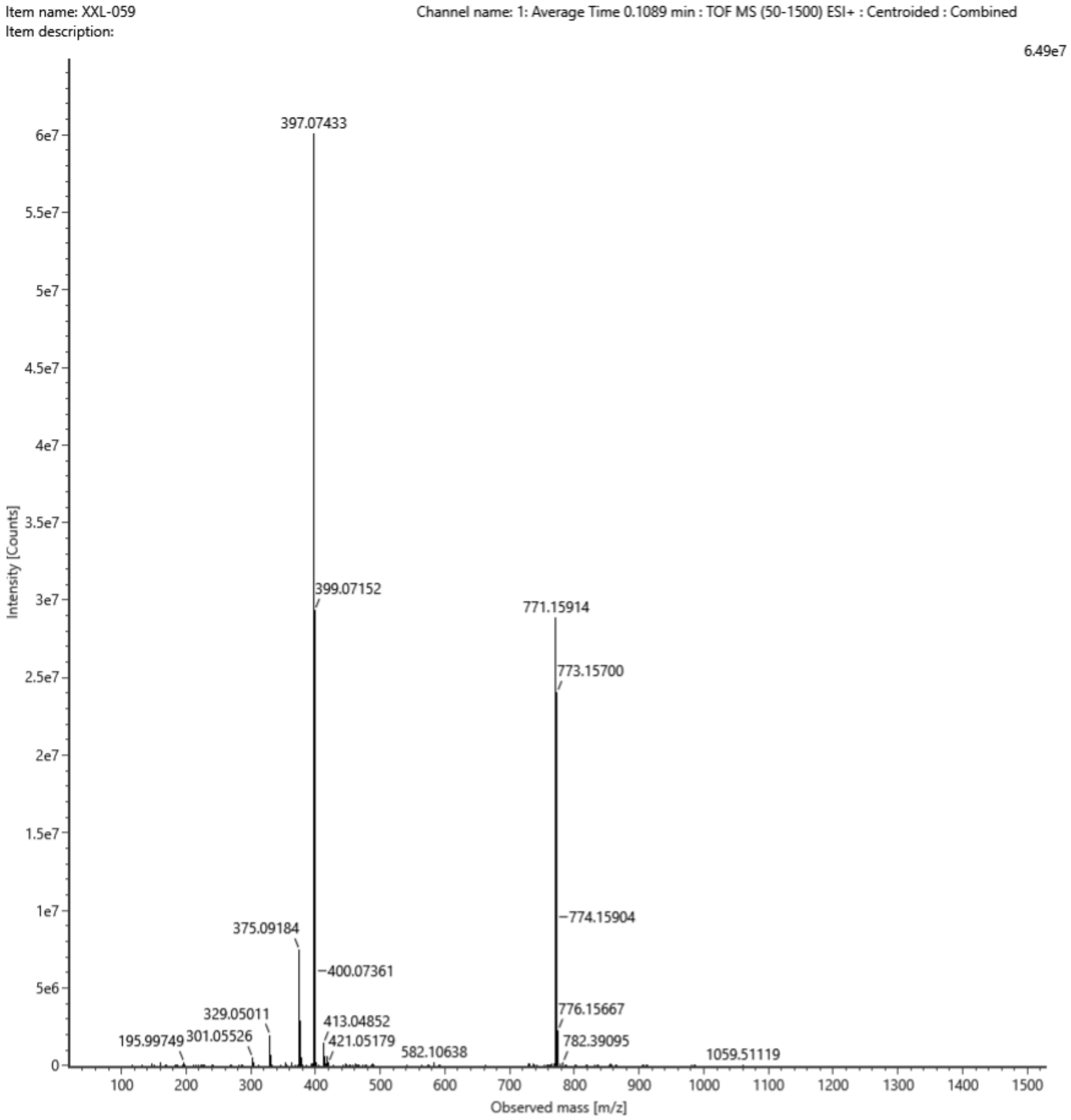

HRMS of compound 4l

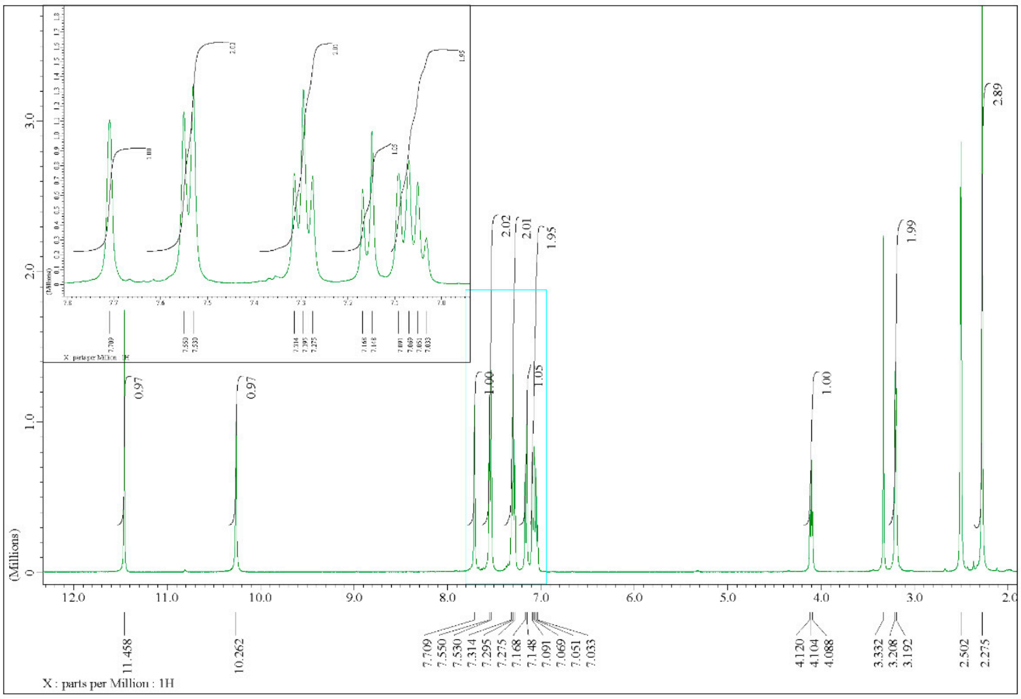

^{1}H NMR of compound 4m

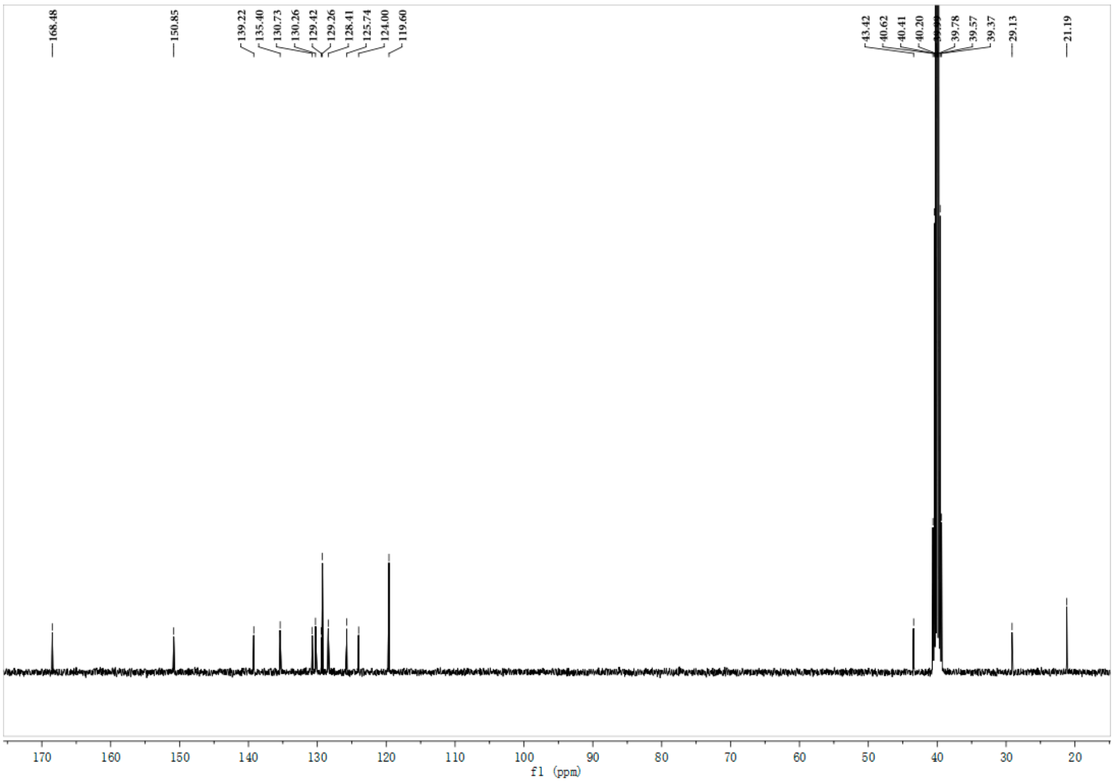

^{13}C NMR of compound 4m

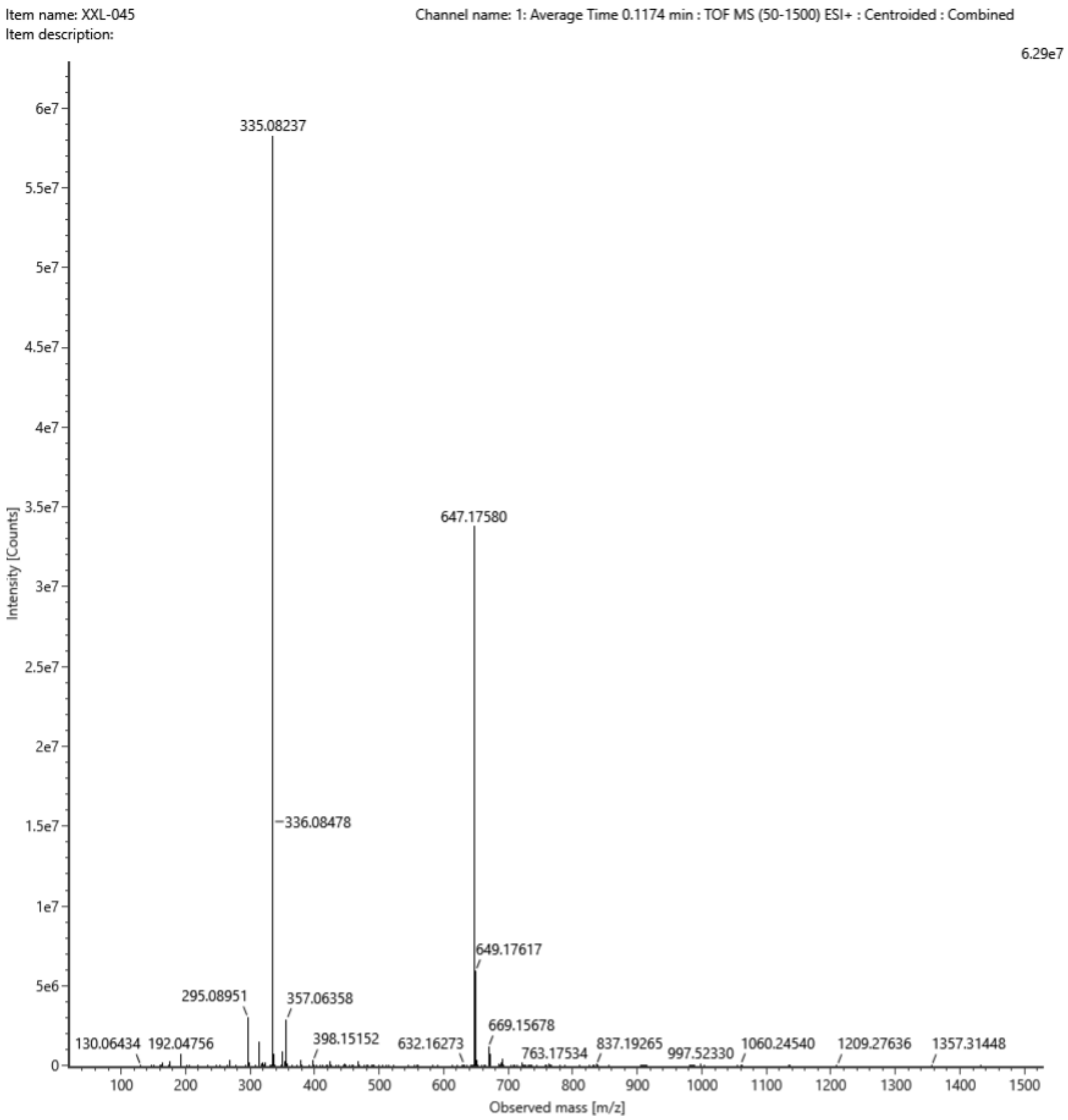

HRMS of compound 4m

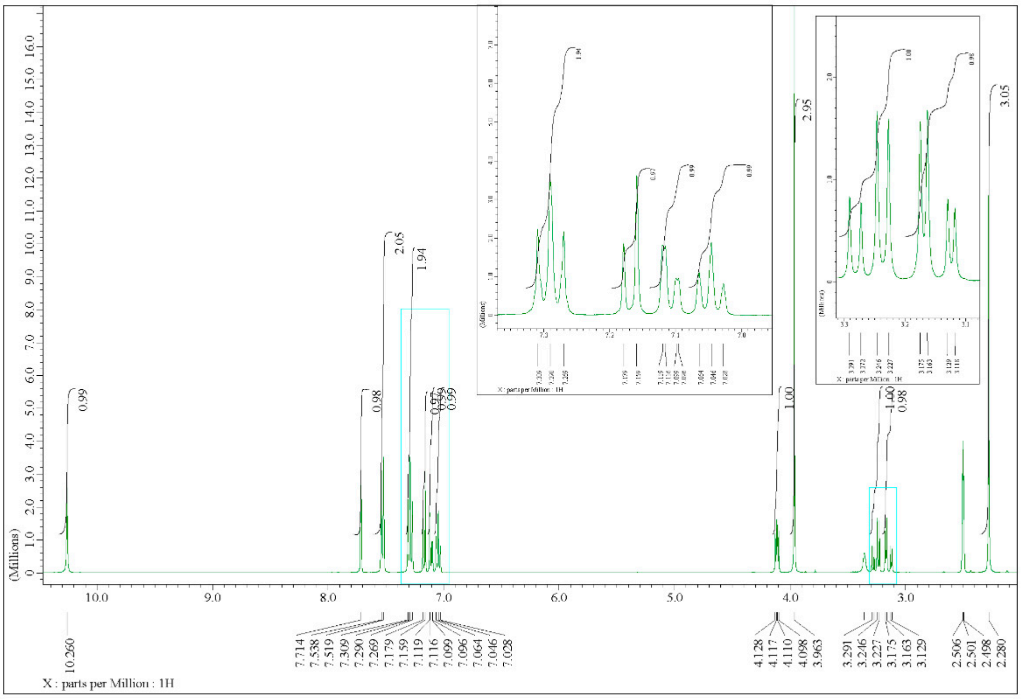

^{1}H NMR of compound 4n

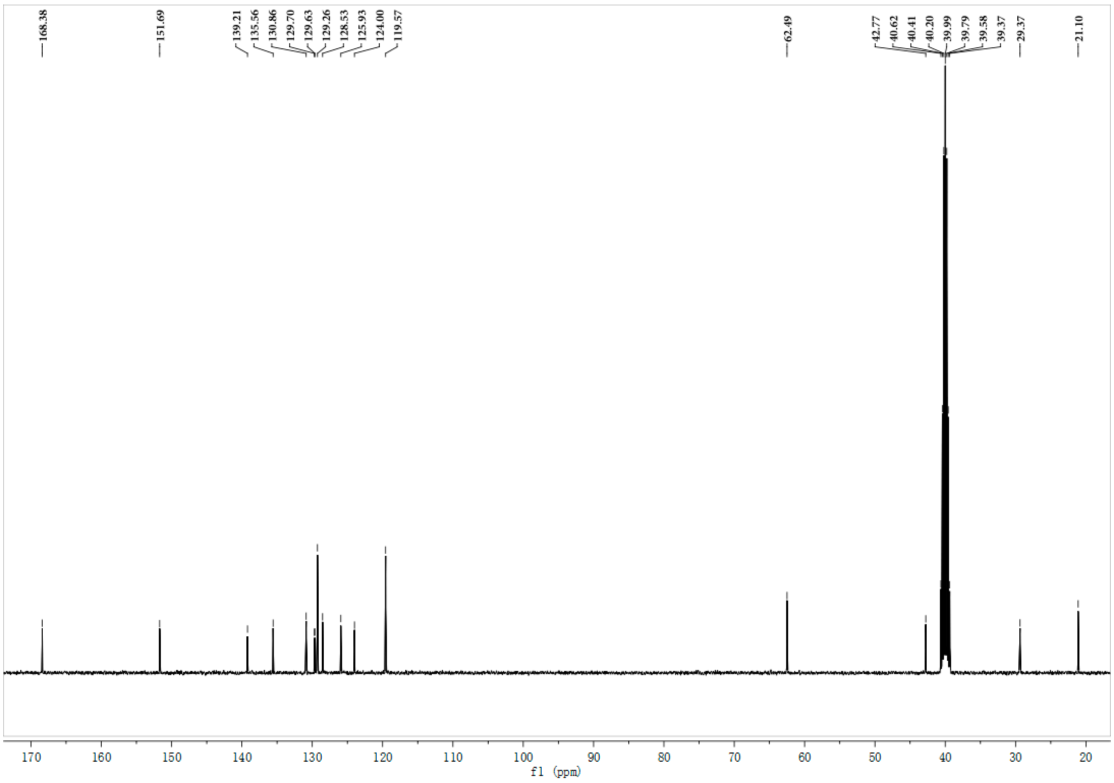

^{13}C NMR of compound 4n

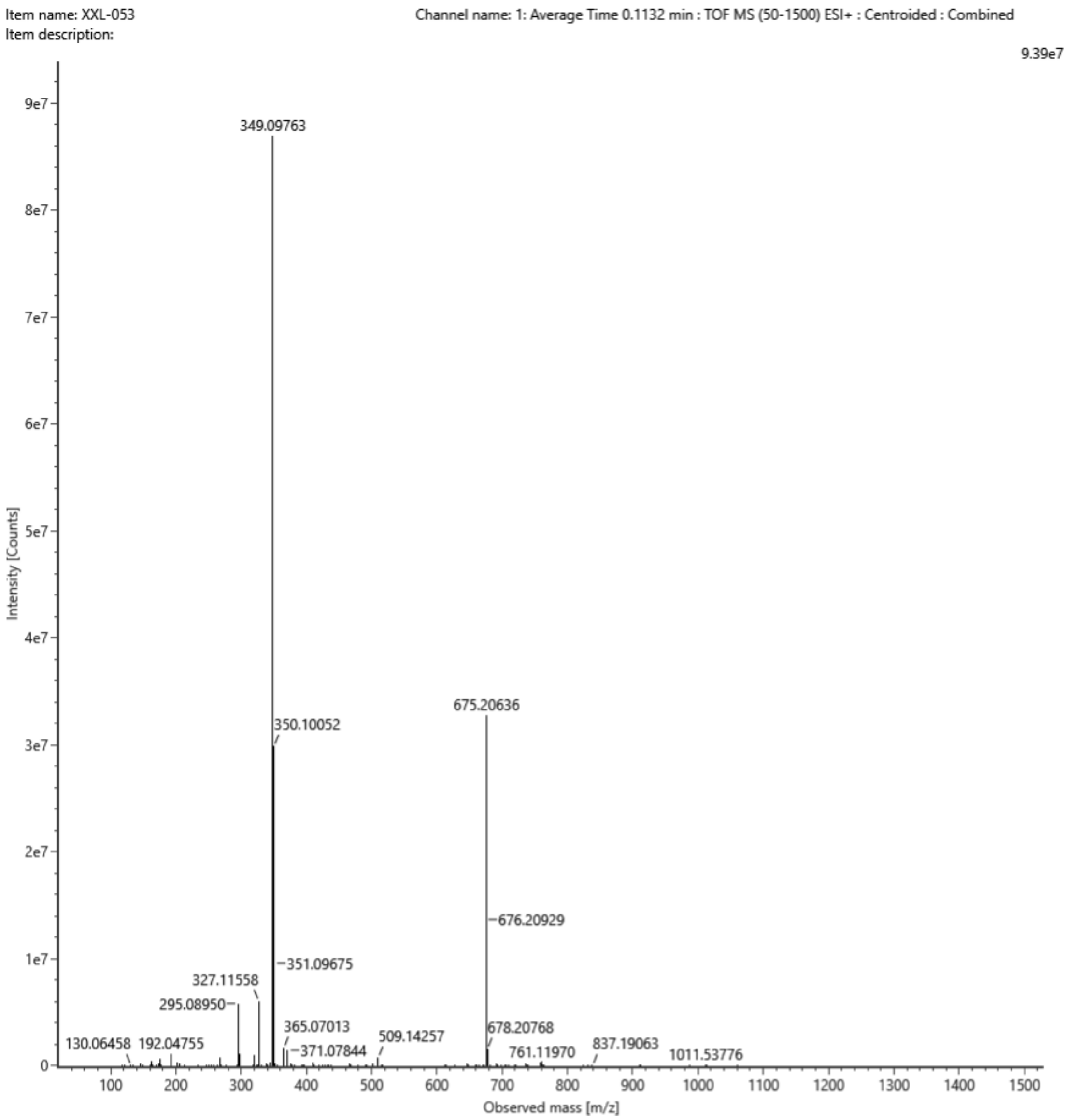

HRMS of compound 4n

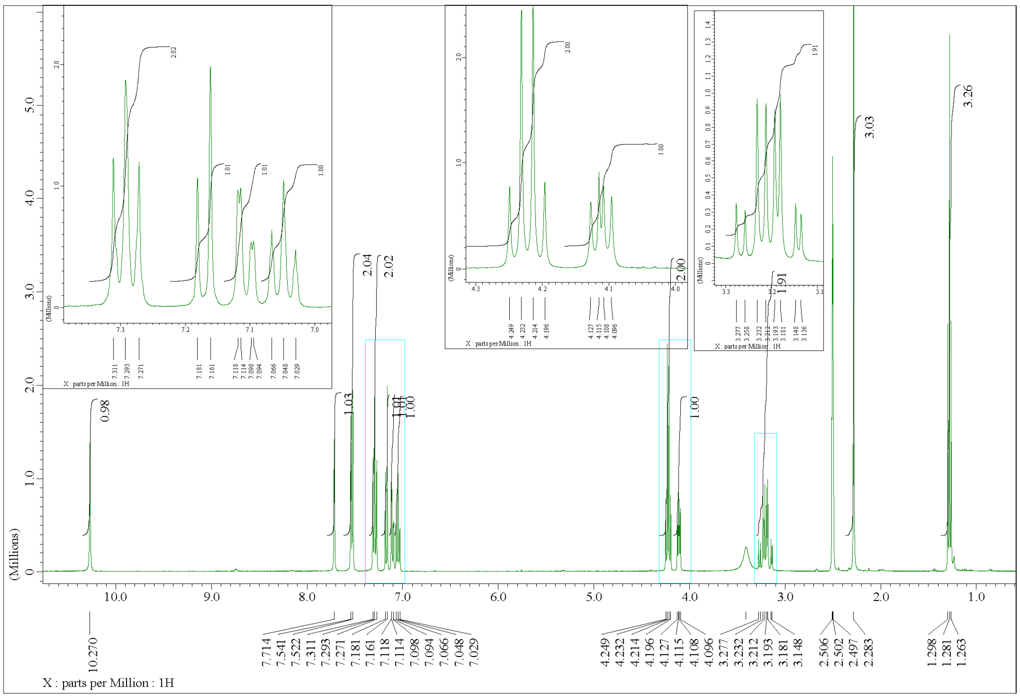

^{1}H NMR of compound 4o

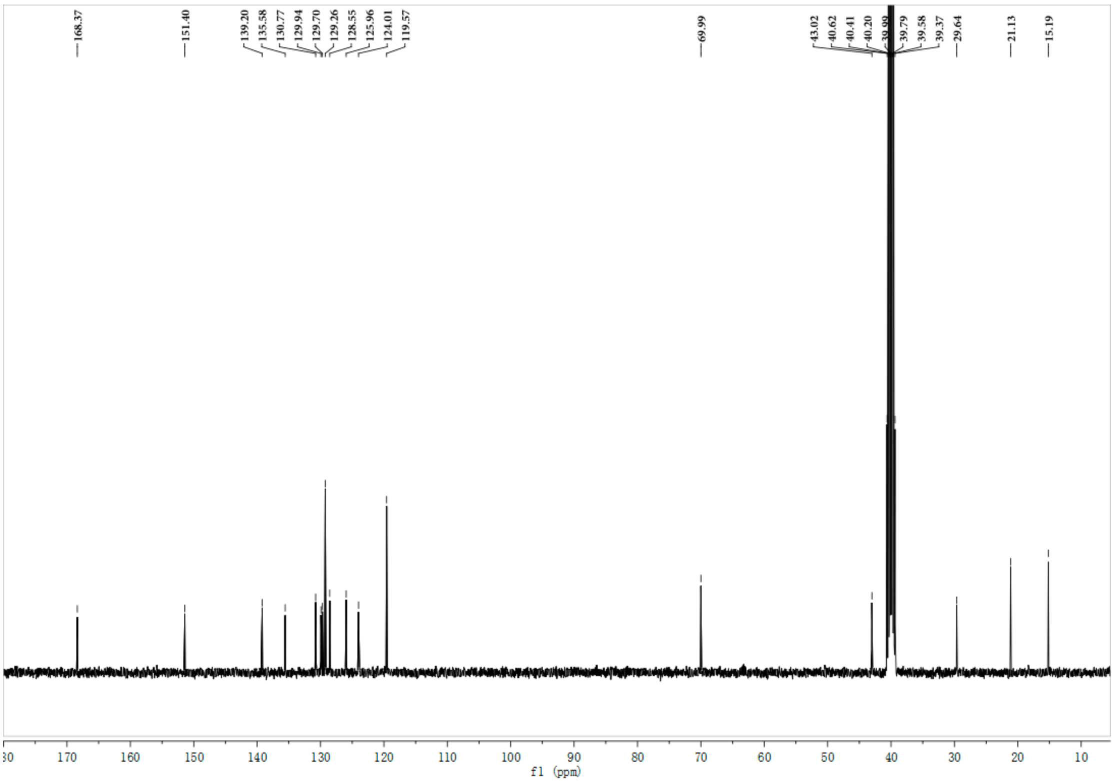

^{13}C NMR of compound 4o

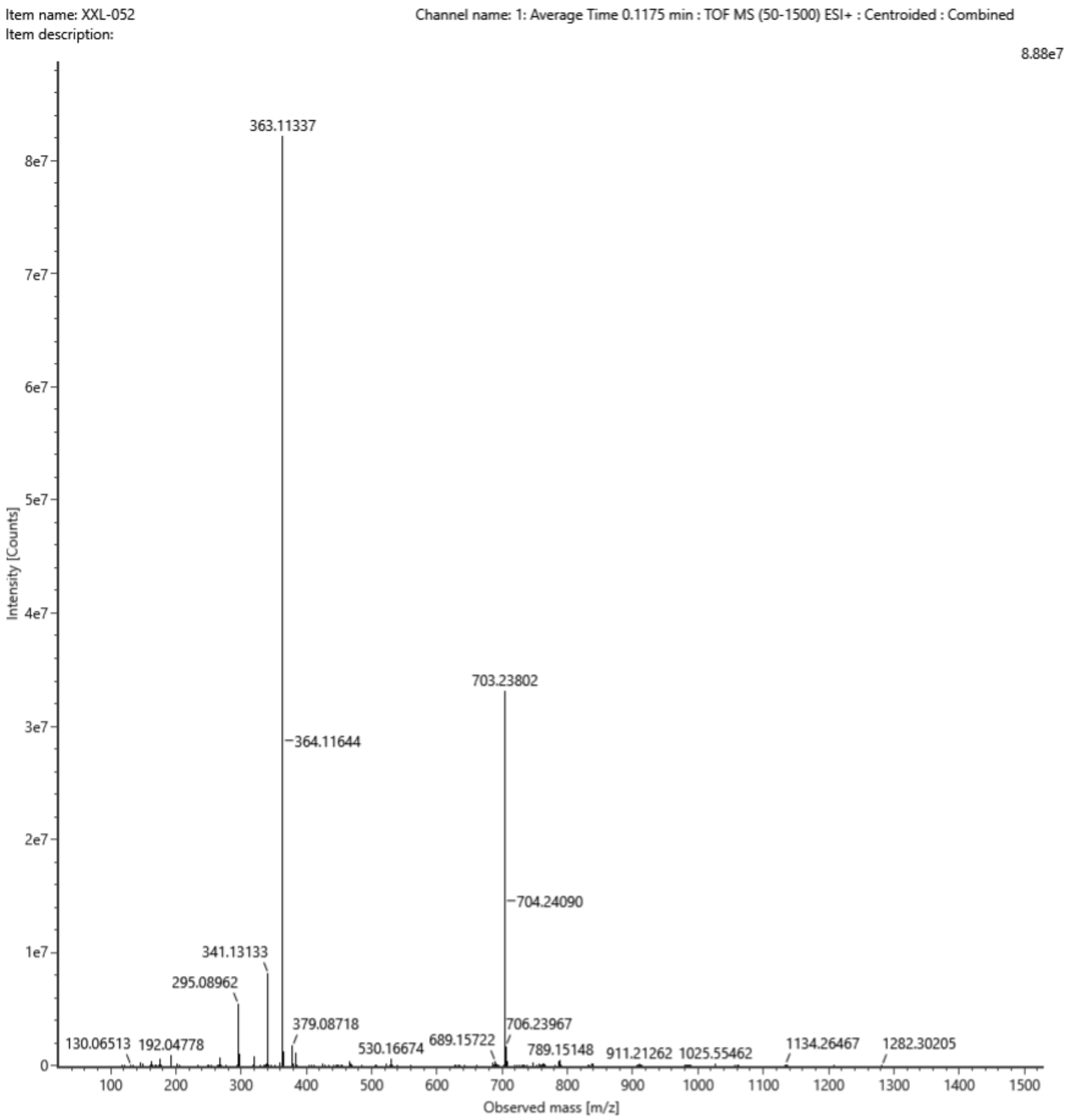

HRMS of compound 4o

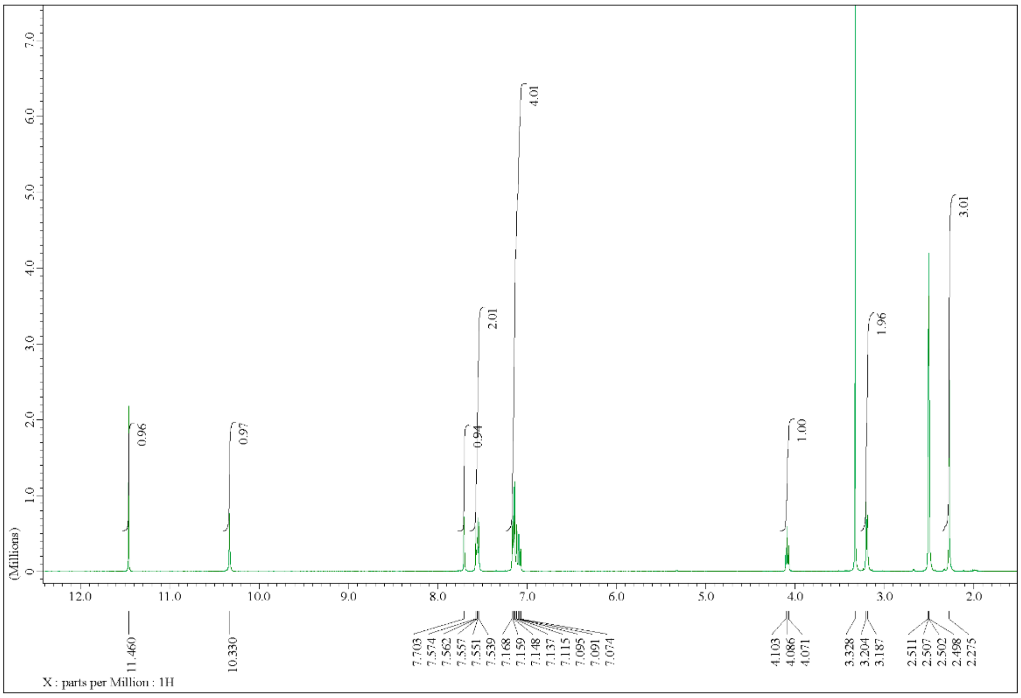

^{1}H NMR of compound 4p

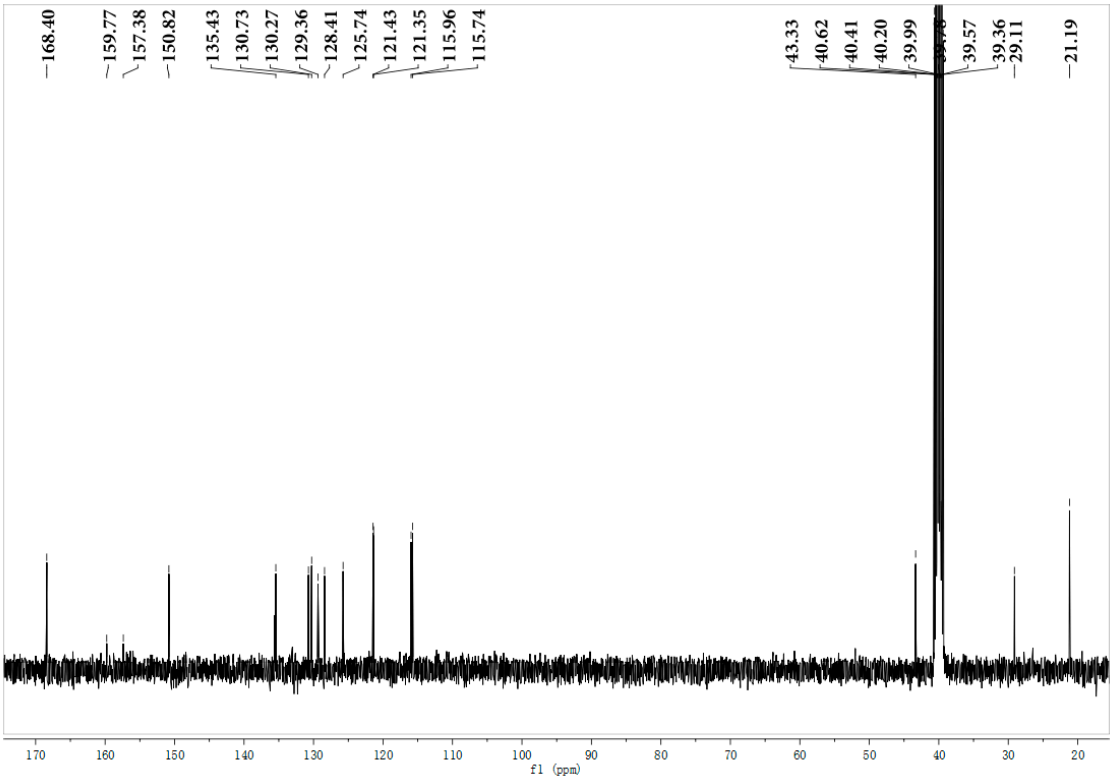

^{13}C NMR of compound 4p

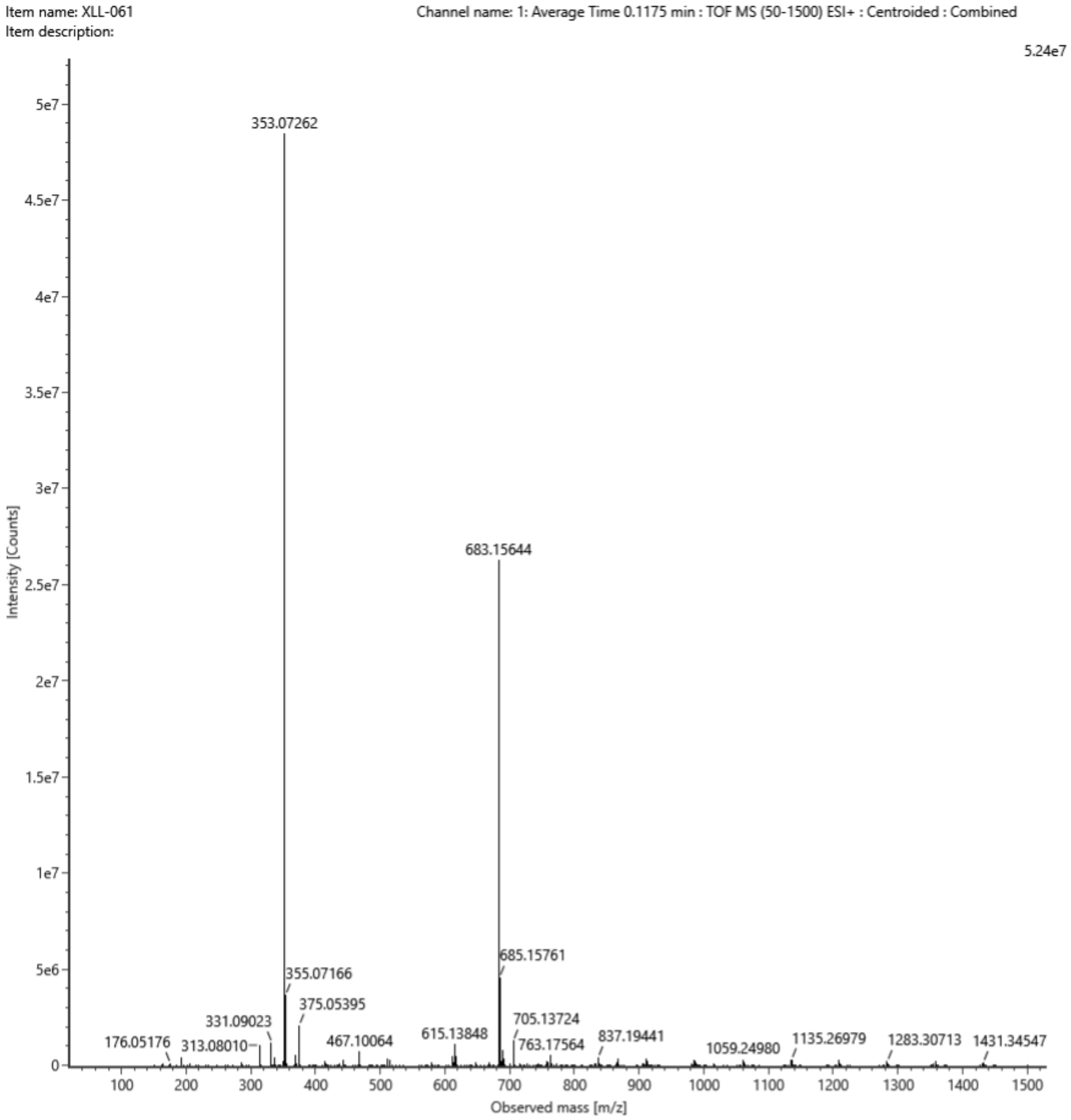

HRMS of compound 4p

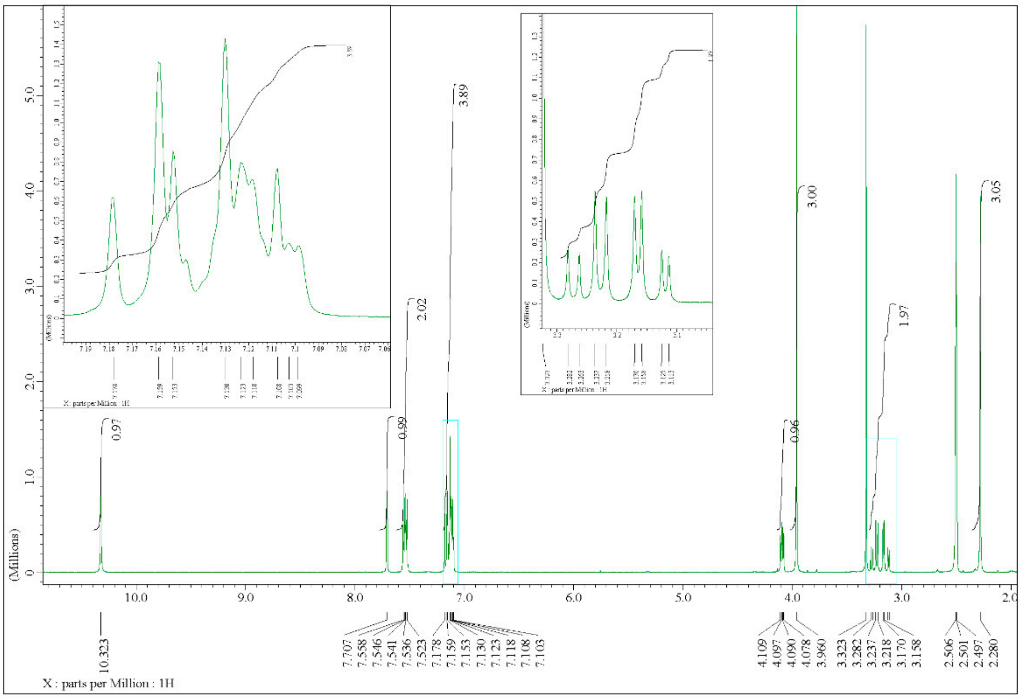

^{1}H NMR of compound 4q

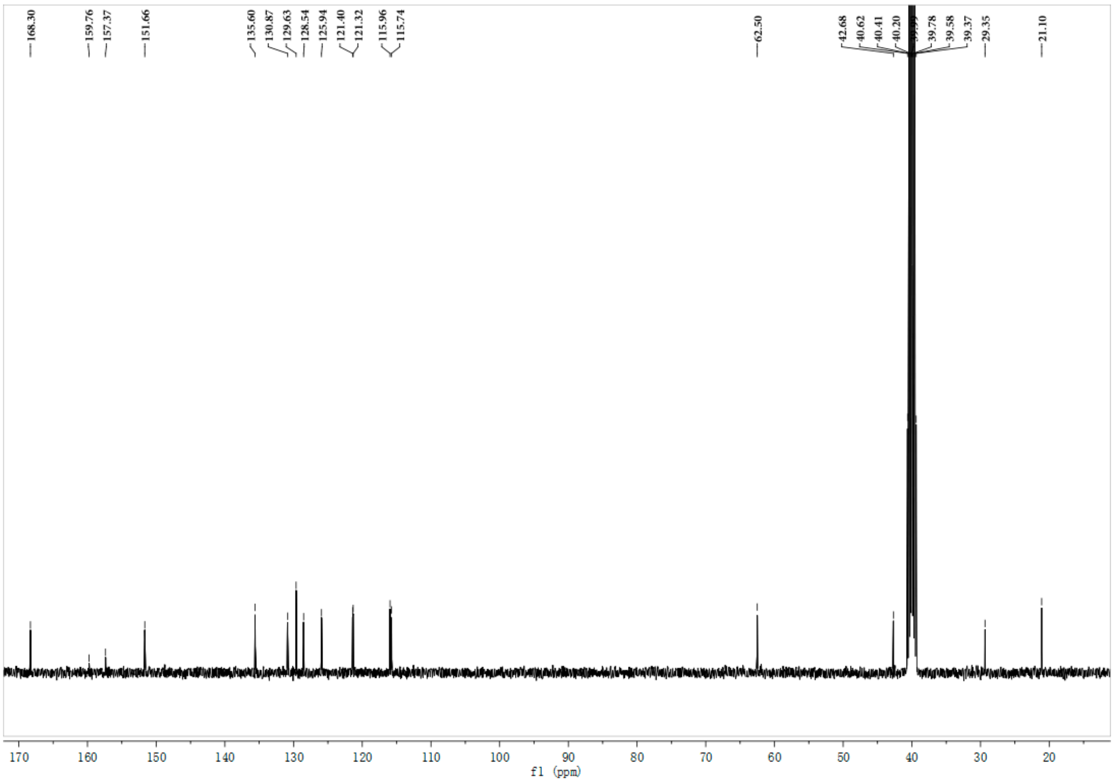

^{13}C NMR of compound 4q

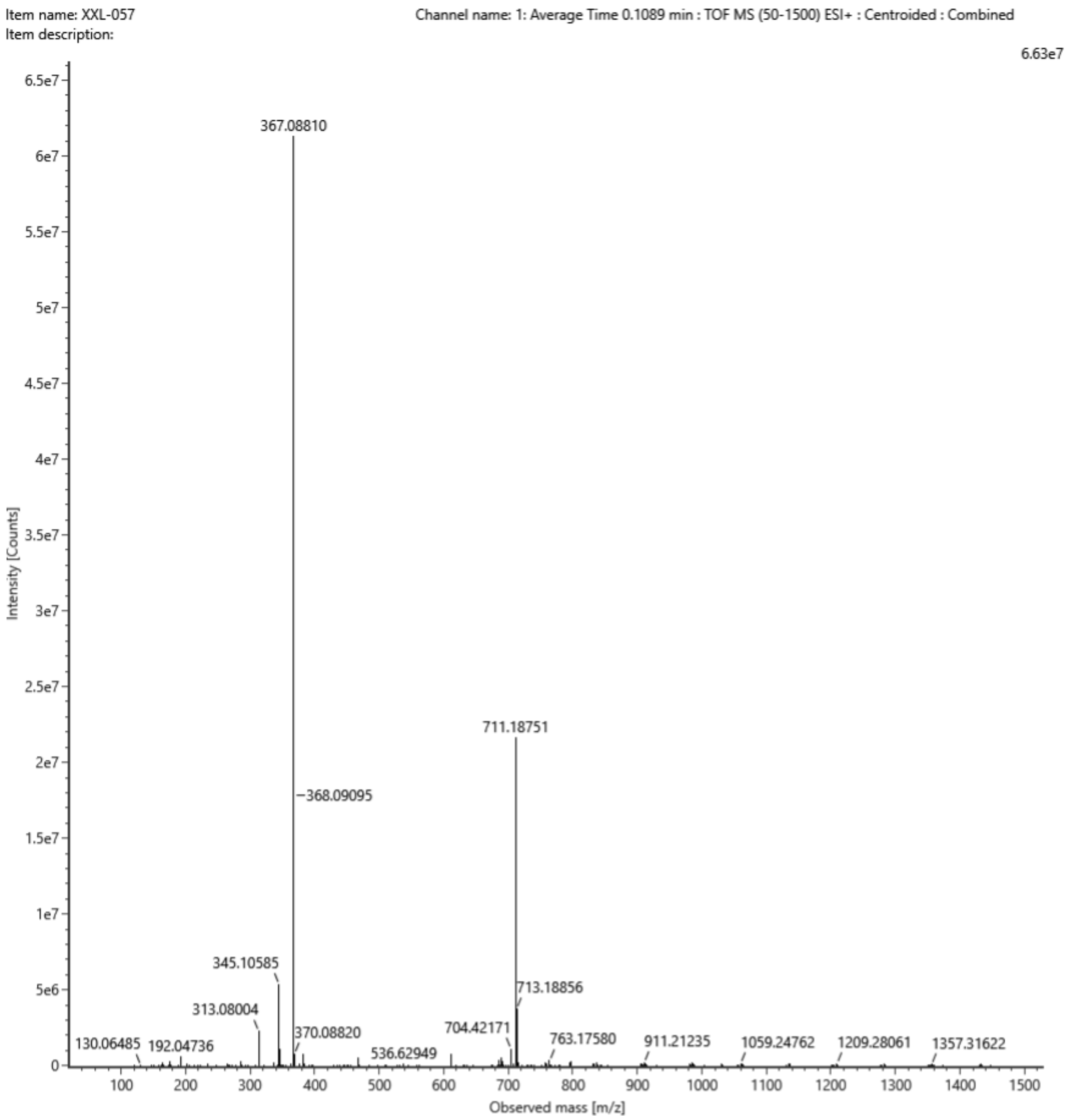

HRMS of compound 4q

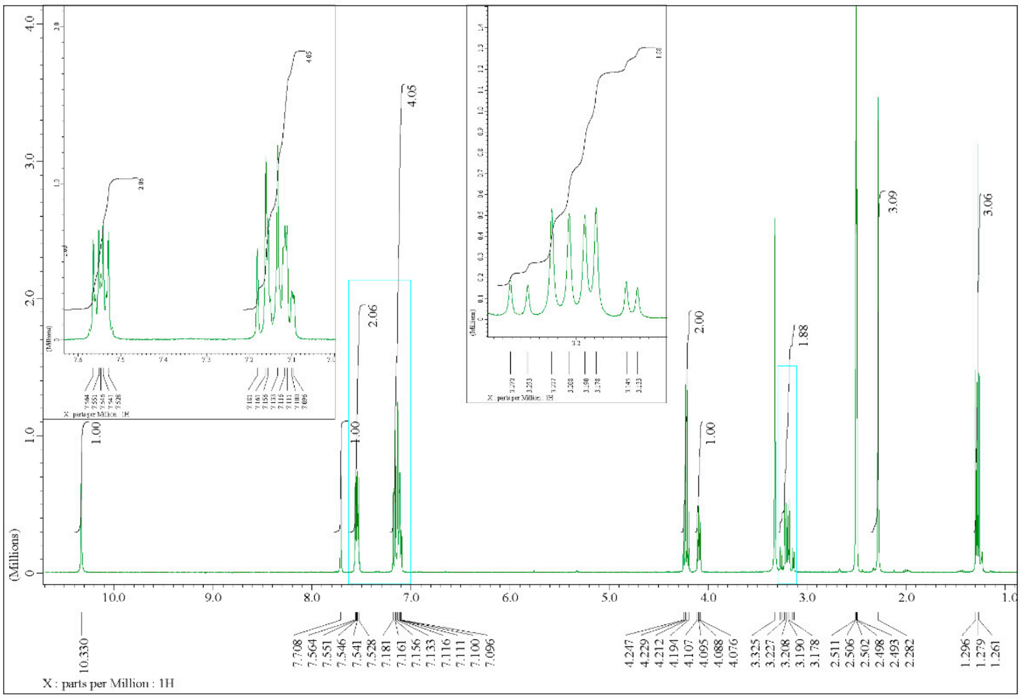

^{1}H NMR of compound 4r

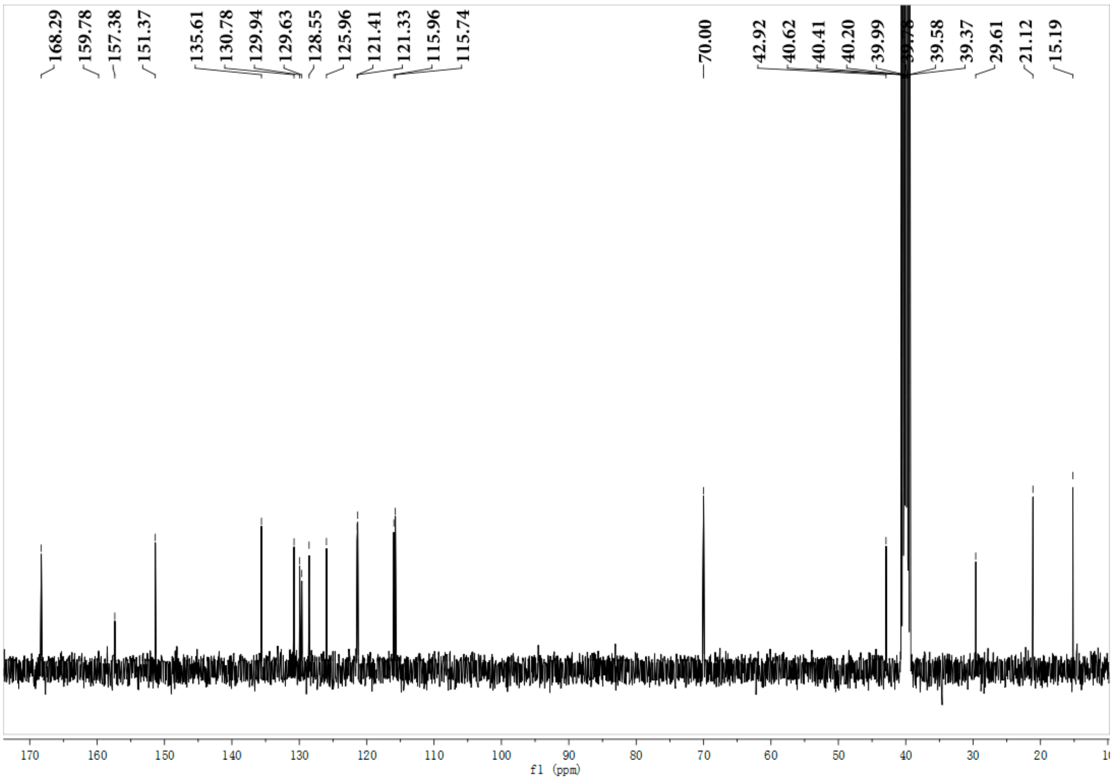

^{13}C NMR of compound 4r

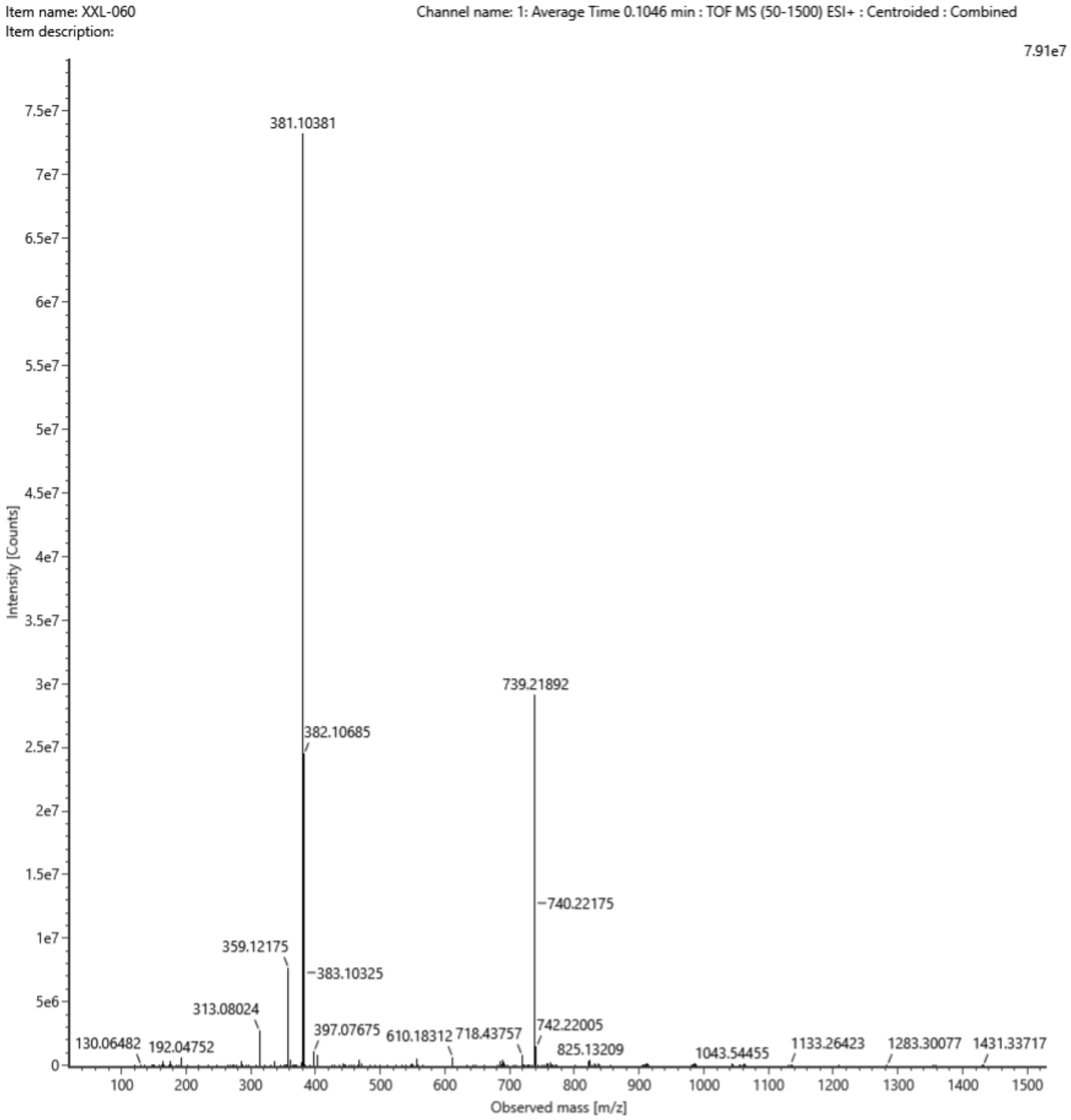

HRMS of compound 4r

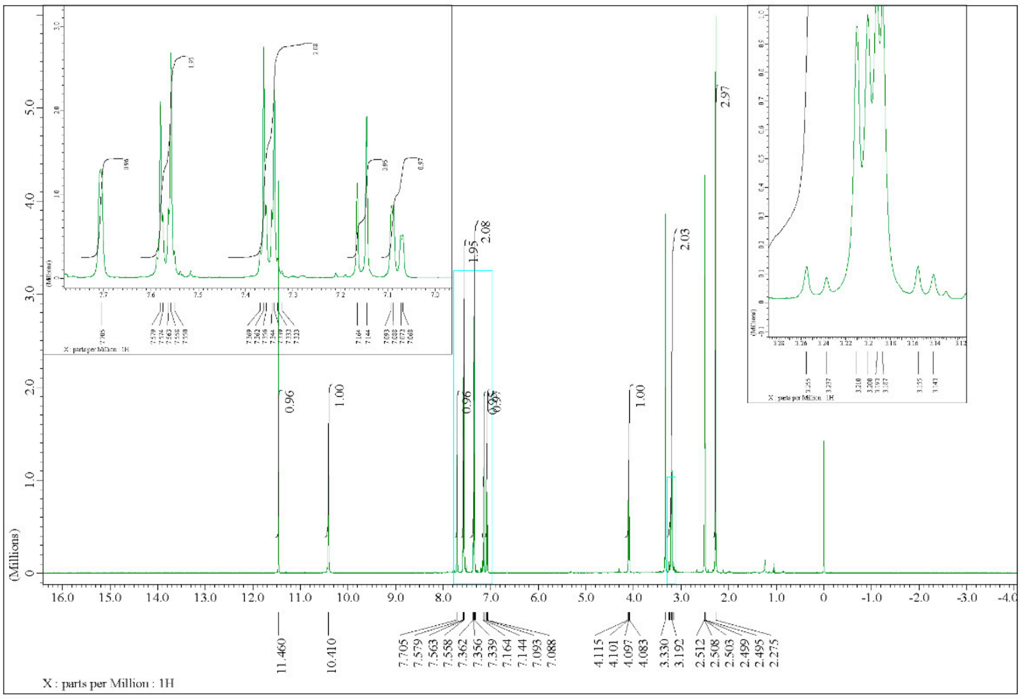

^{1}H NMR of compound 4s

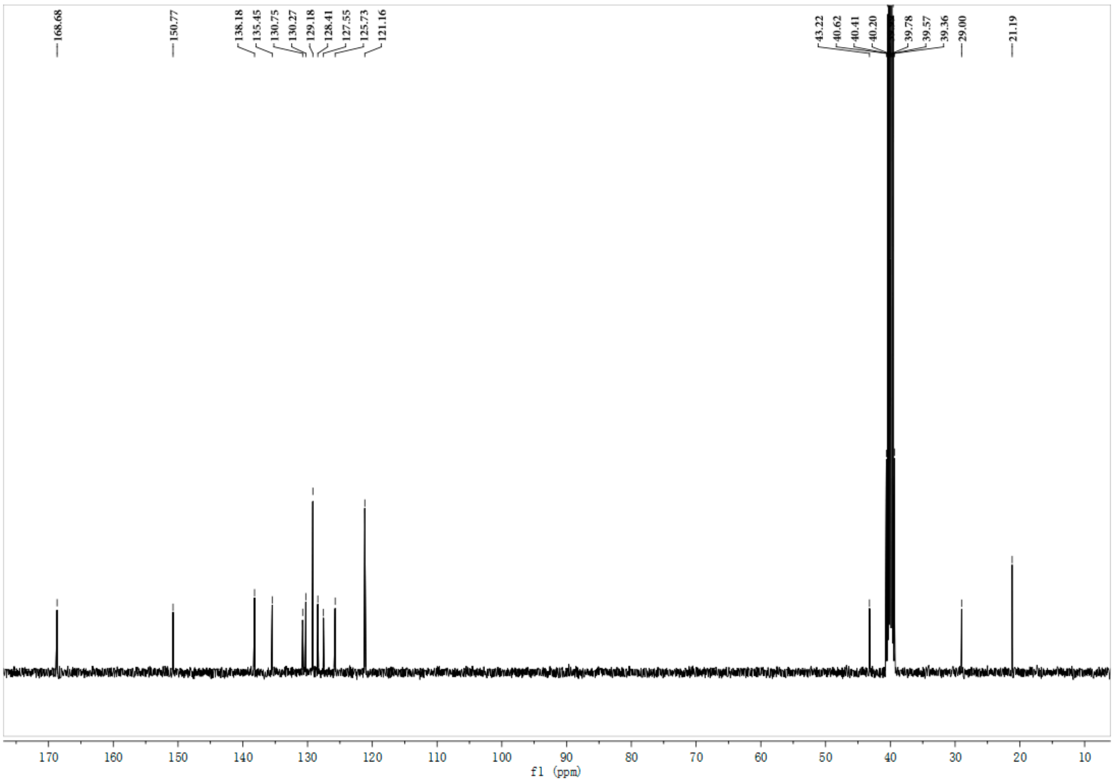

^{13}C NMR of compound 4s

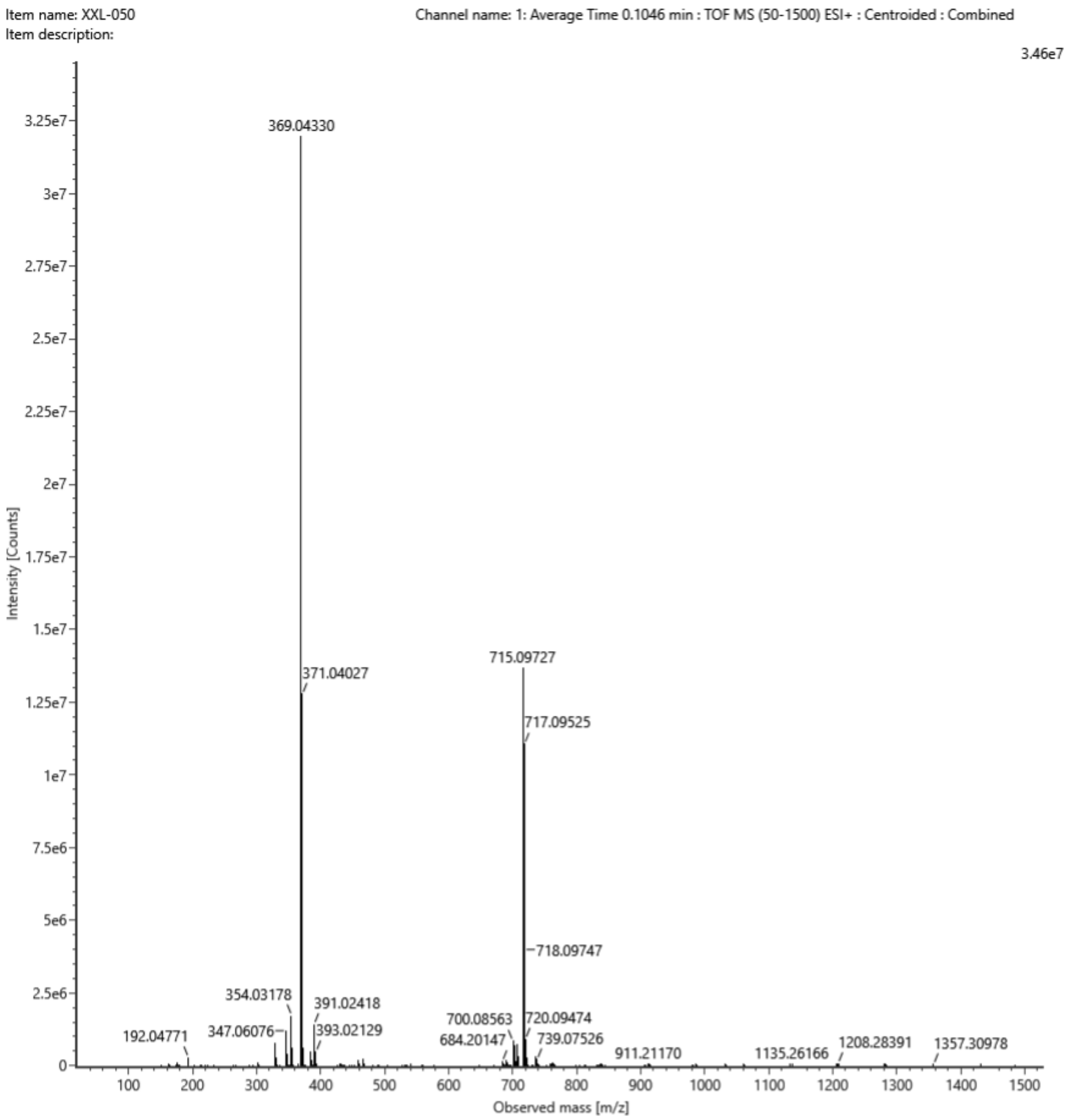

HRMS of compound 4s

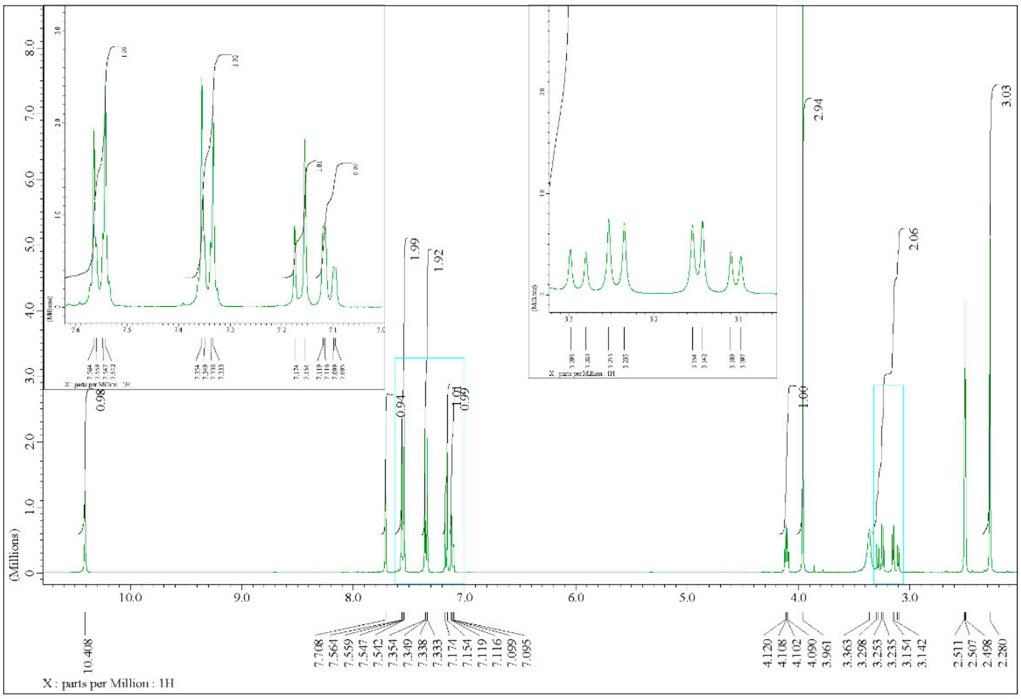

^{1}H NMR of compound 4t

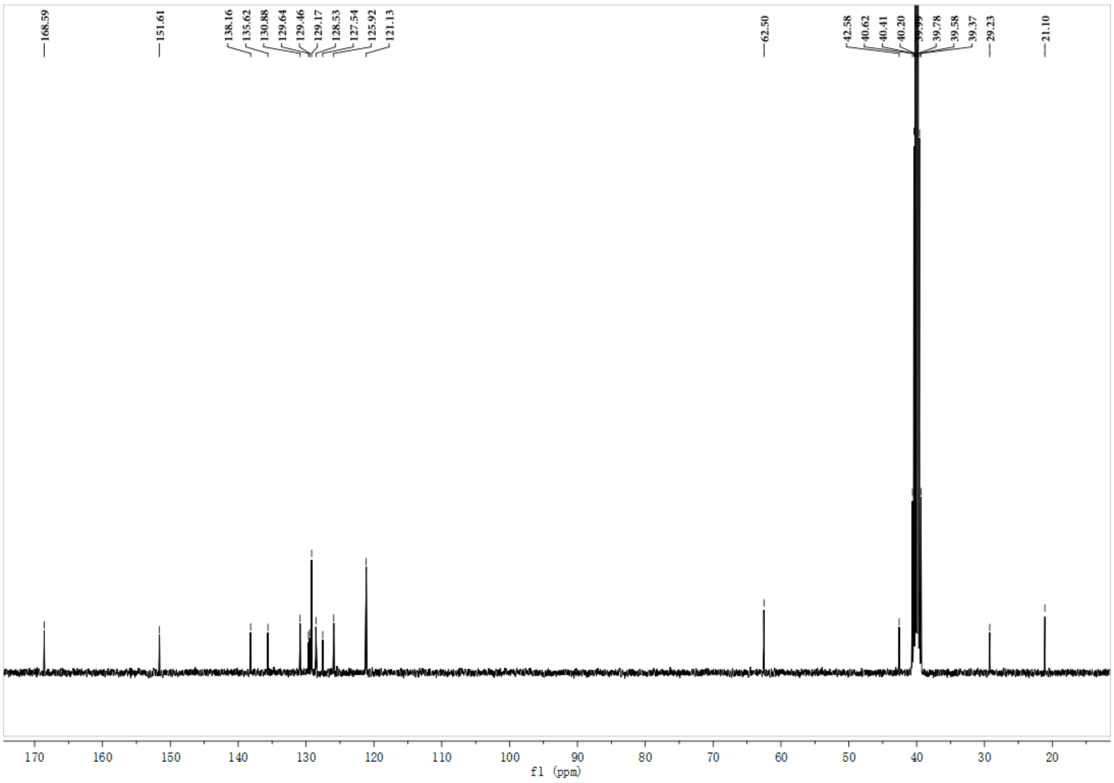

^{13}C NMR of compound 4t

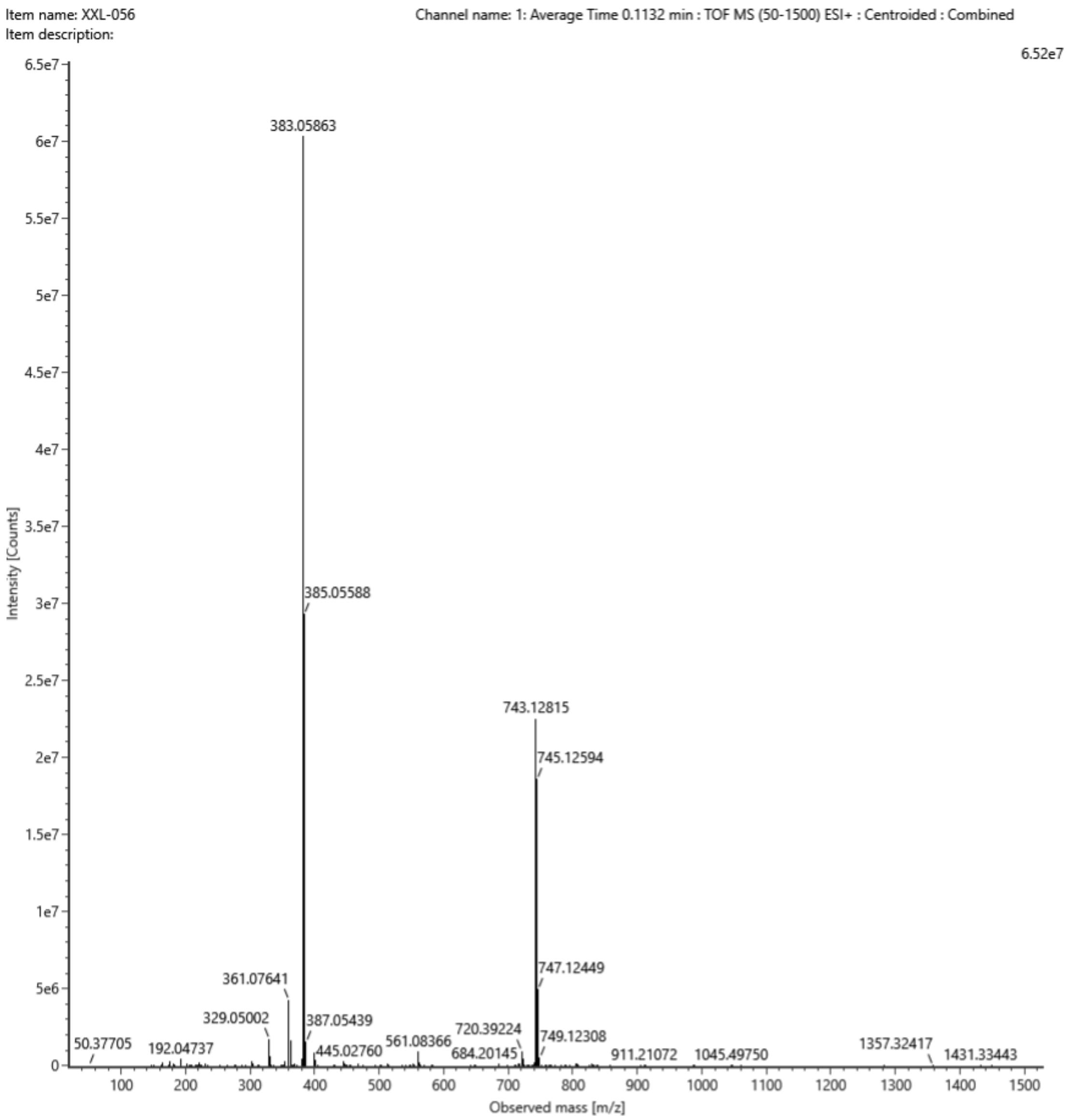

HRMS of compound 4t

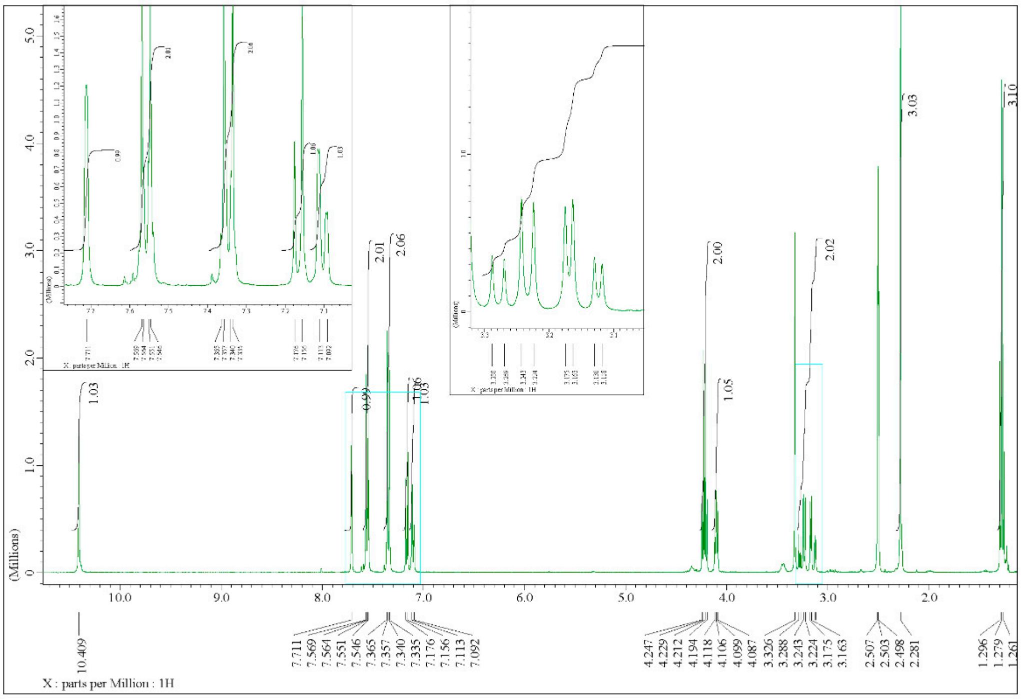

^{1}H NMR of compound 4u

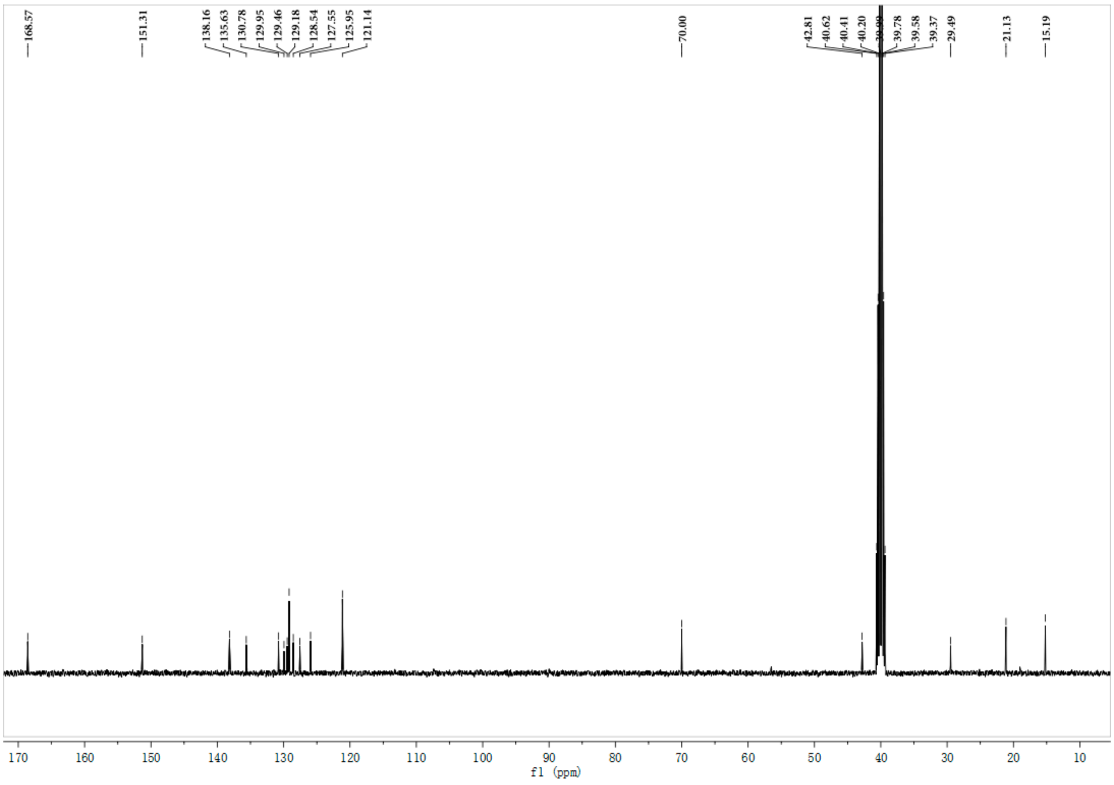

^{13}C NMR of compound 4u

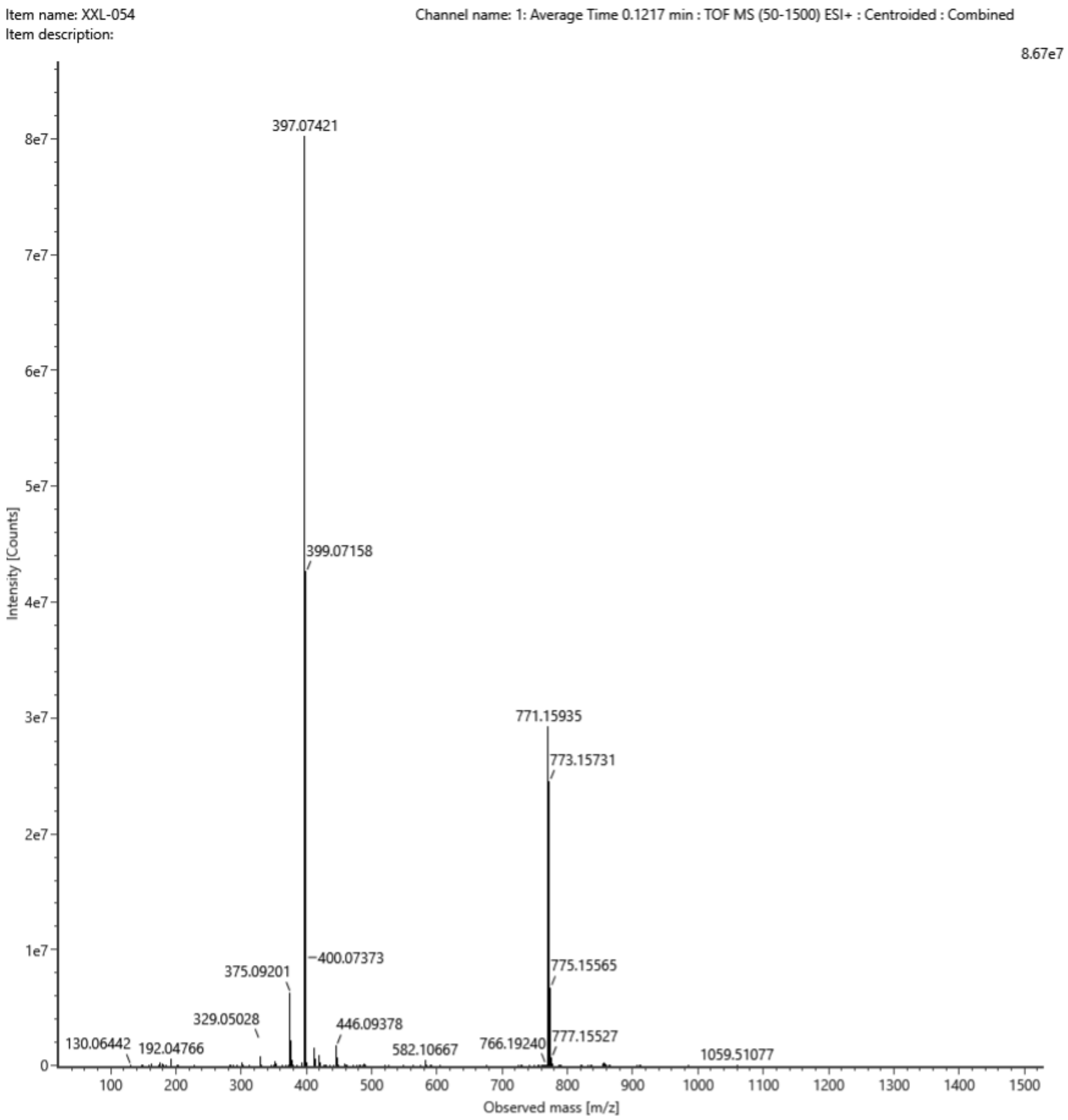

HRMS of compound 4u

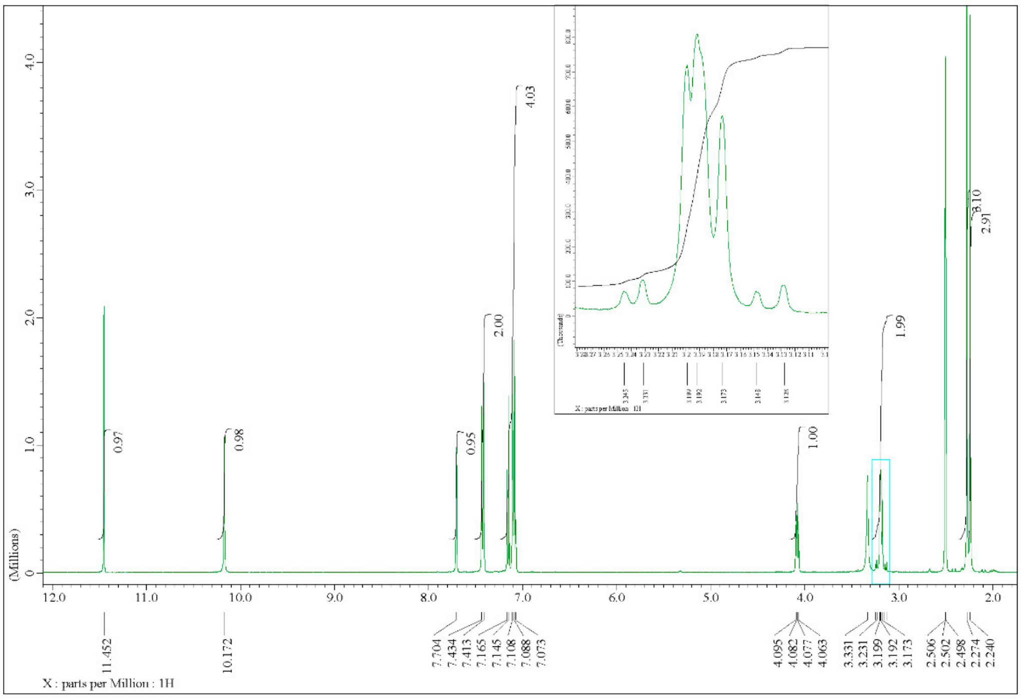

^{1}H NMR of compound 4v

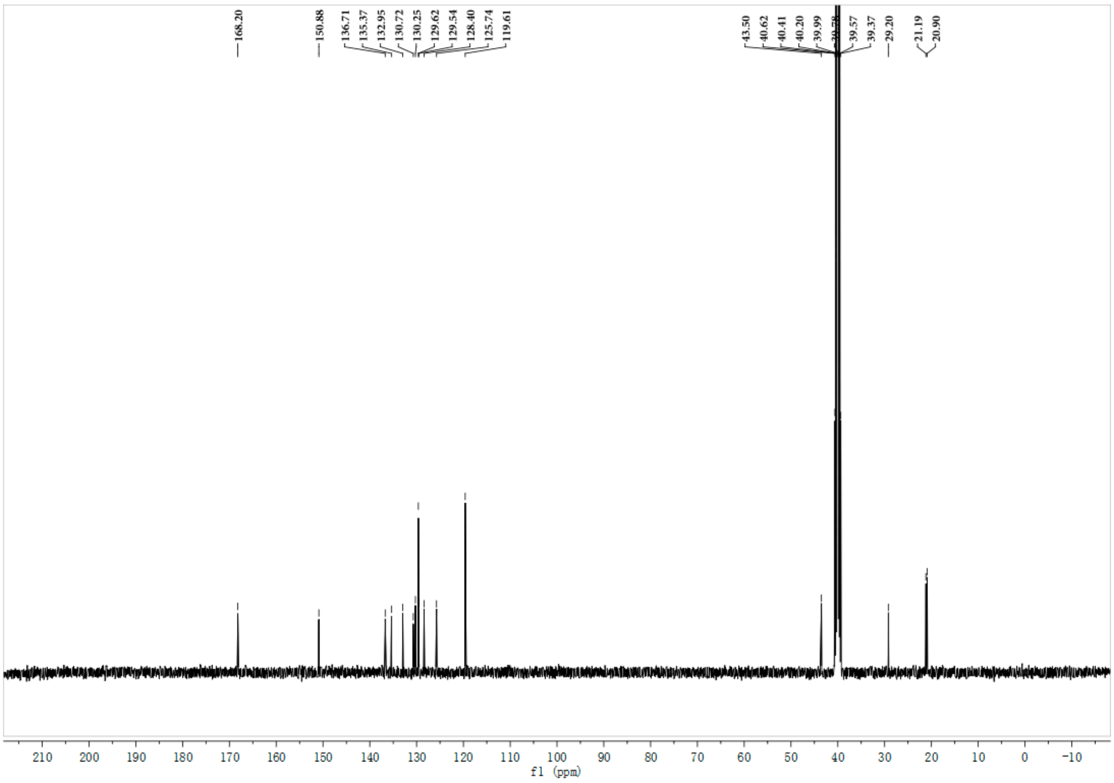

^{13}C NMR of compound 4v

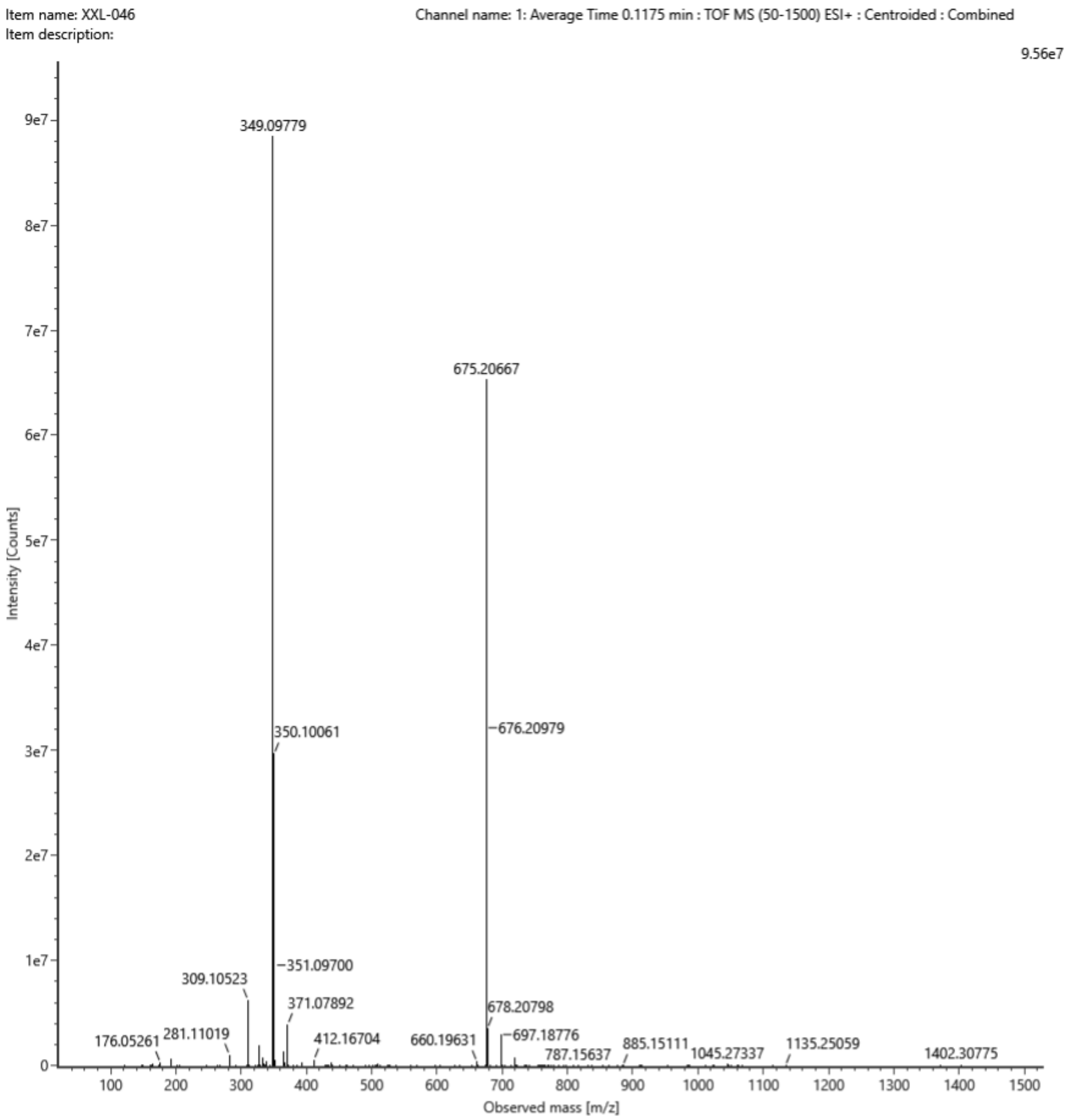

HRMS of compound 4v

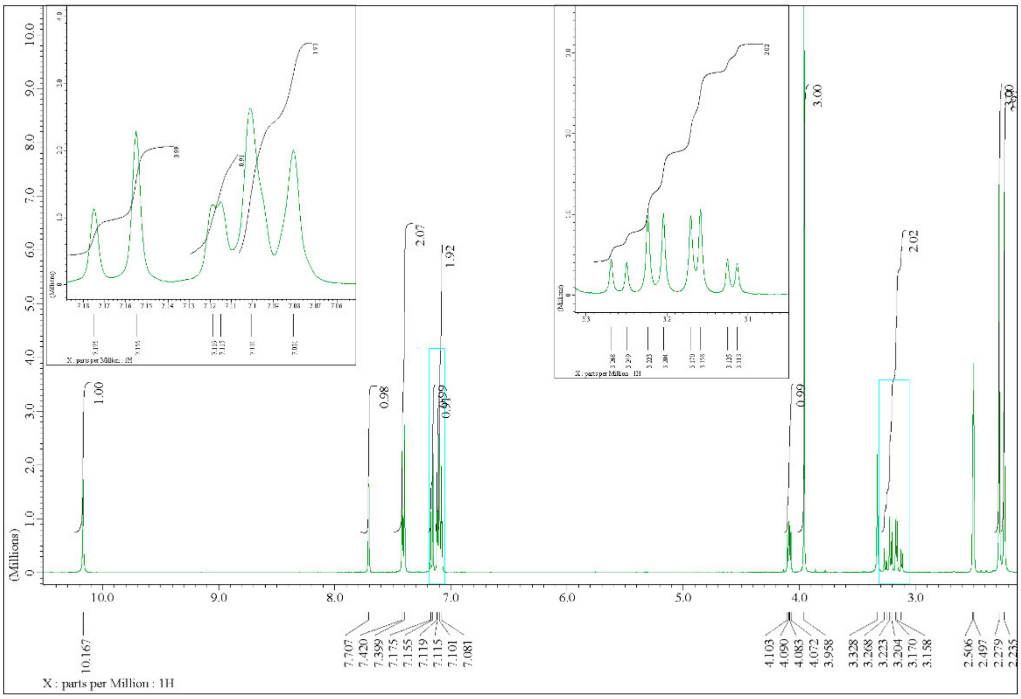

^{1}H NMR of compound 4w

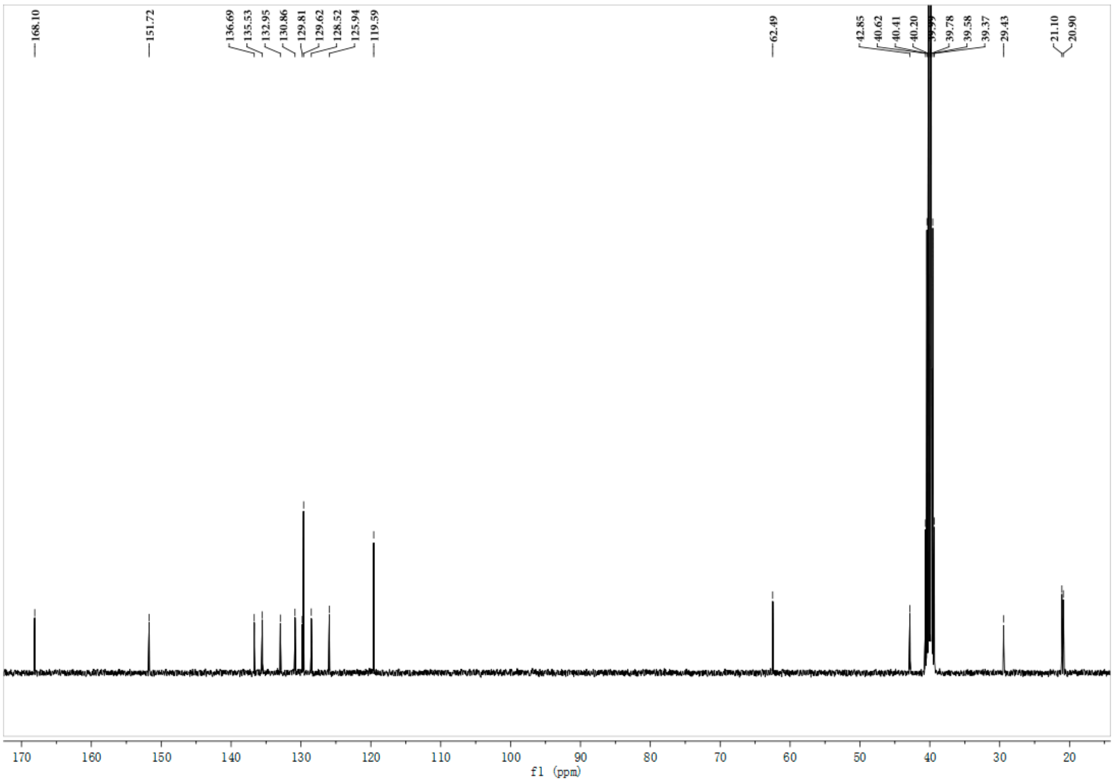

^{13}C NMR of compound 4w

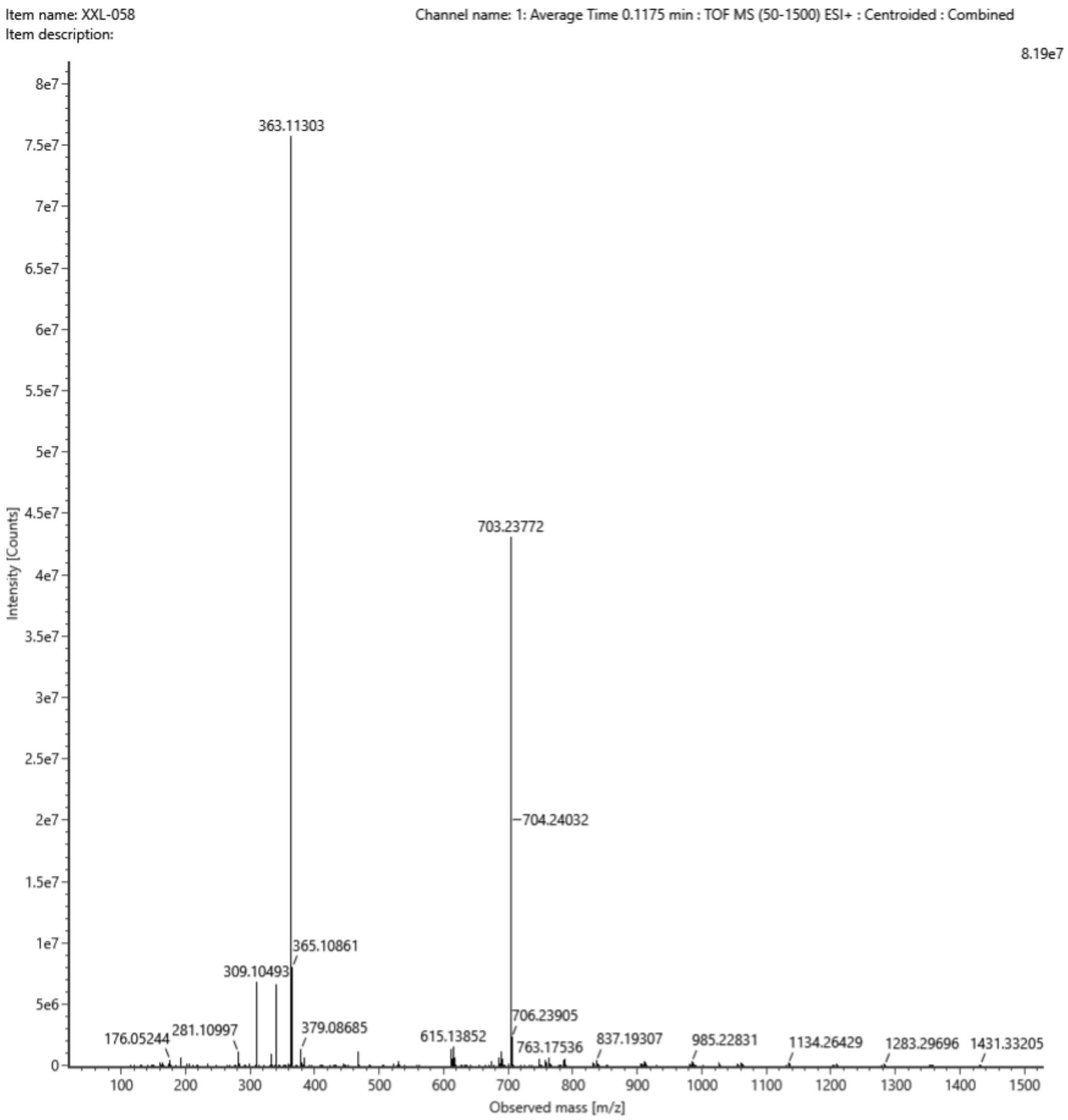

HRMS of compound 4w

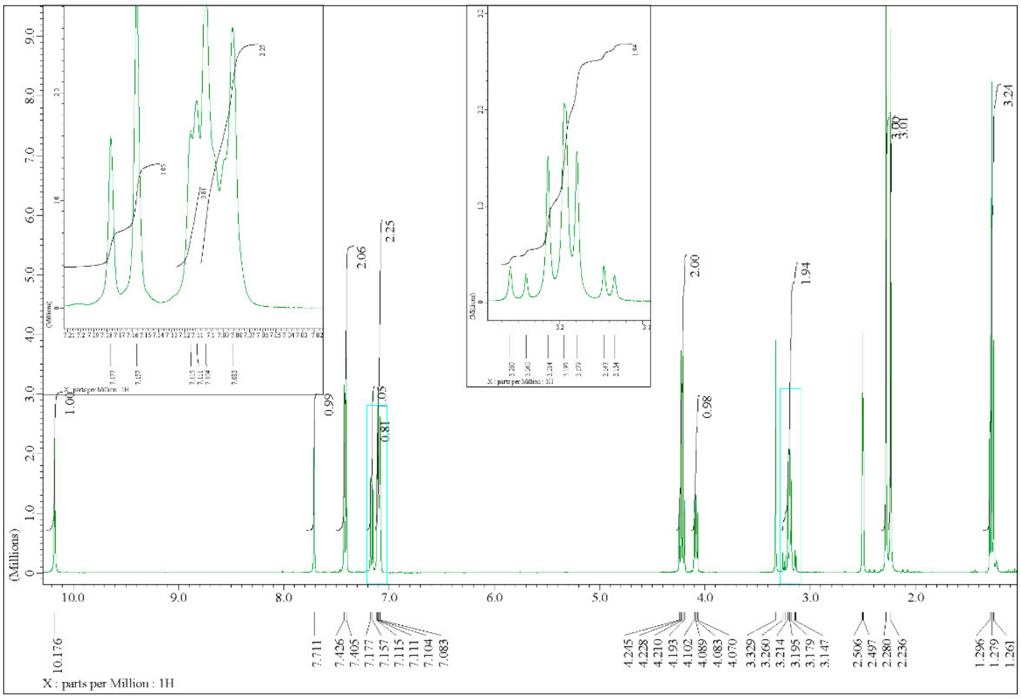

^{1}H NMR of compound 4x

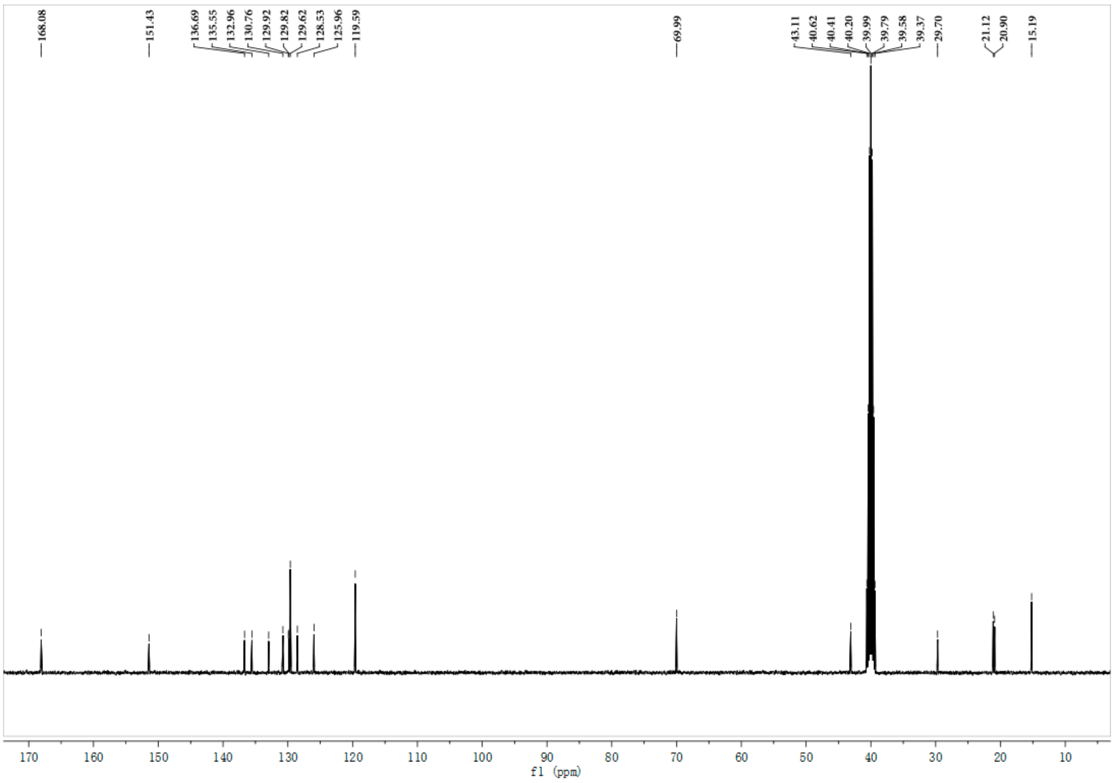

^{13}C NMR of compound 4x

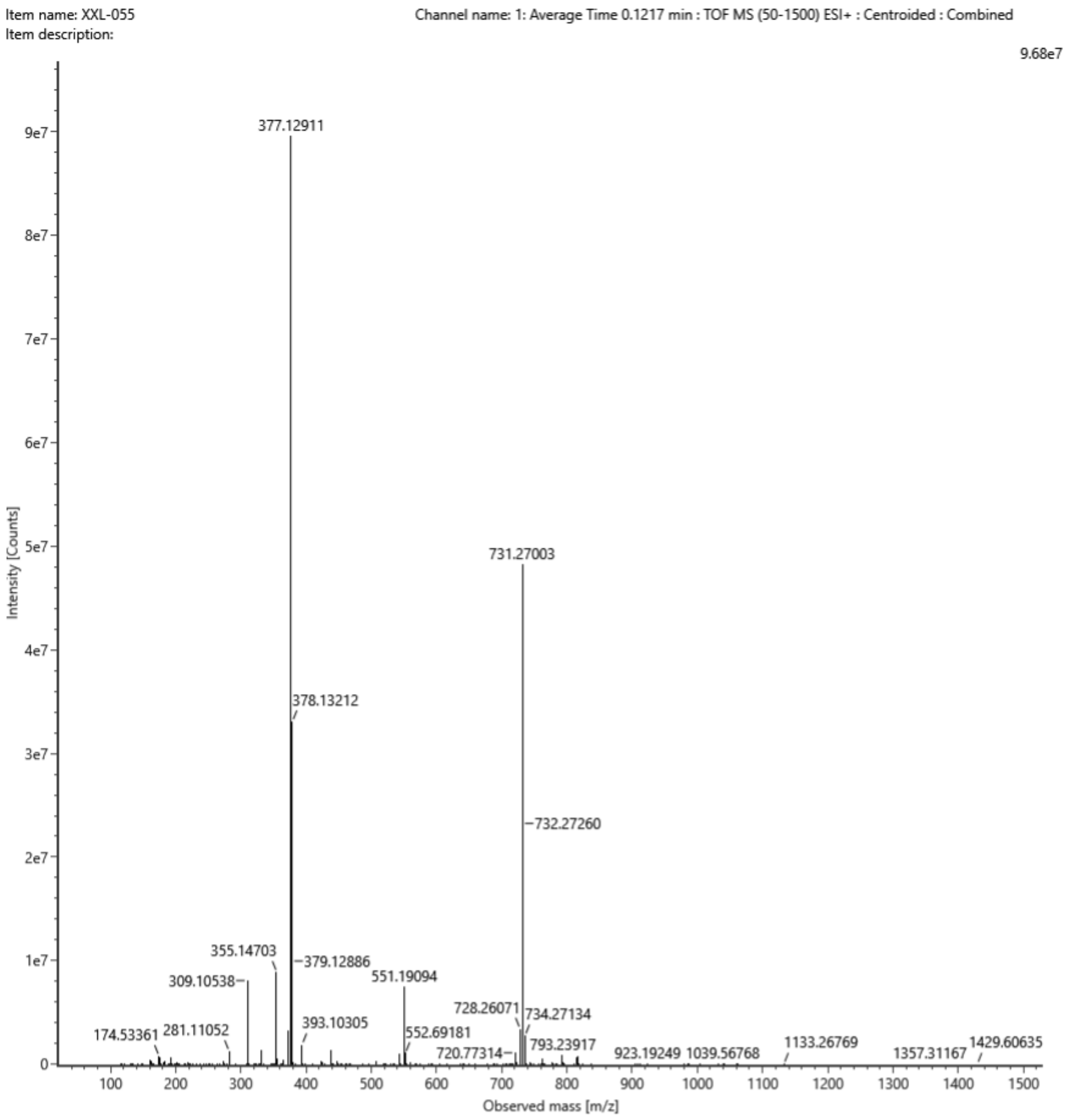

HRMS of compound 4x
